# **CSA:** A high-throughput **c**hromosome-**s**cale **a**ssembly pipeline for vertebrate genomes

**DOI:** 10.1093/gigascience/giaa034

**Published:** 2020-05-25

**Authors:** Heiner Kuhl, Ling Li, Sven Wuertz, Matthias Stöck, Xu-Fang Liang, Christophe Klopp

**Affiliations:** 1 Department of Ecophysiology and Aquaculture, Leibniz-Institute of Freshwater Ecology and Inland Fisheries (IGB), Müggelseedamm 310, 12587 Berlin, Germany; 2 College of Fisheries, Chinese Perch Research Center, Huazhong Agricultural University; Innovation Base for Chinese Perch Breeding, Key Lab of Freshwater Animal Breeding, Ministry of Agriculture, No.1 Shizishan Street, Hongshan District, 430070 Wuhan, Hubei Province, P.R. China; 3 Sigenae, Bioinfo Genotoul, Mathématiques et Informatique Appliquées de Toulouse, INRAe, 24 Chemin de Borde Rouge, 31320 Auzeville-Tolosane, Castanet Tolosan, France

**Keywords:** genome assembly, genome scaffolding, long-read, comparative genomics, genome evolution, chromosomes, vertebrates

## Abstract

**Background:**

Easy-to-use and fast bioinformatics pipelines for long-read assembly that go beyond the contig level to generate highly continuous chromosome-scale genomes from raw data remain scarce.

**Result:**

Chromosome-Scale Assembler (CSA) is a novel computationally highly efficient bioinformatics pipeline that fills this gap. CSA integrates information from scaffolded assemblies (e.g., Hi-C or 10X Genomics) or even from diverged reference genomes into the assembly process. As CSA performs automated assembly of chromosome-sized scaffolds, we benchmark its performance against state-of-the-art reference genomes, i.e., conventionally built in a laborious fashion using multiple separate assembly tools and manual curation. CSA increases the contig lengths using scaffolding, local re-assembly, and gap closing. On certain datasets, initial contig N50 may be increased up to 4.5-fold. For smaller vertebrate genomes, chromosome-scale assemblies can be achieved within 12 h using low-cost, high-end desktop computers. Mammalian genomes can be processed within 16 h on compute-servers. Using diverged reference genomes for fish, birds, and mammals, we demonstrate that CSA calculates chromosome-scale assemblies from long-read data and genome comparisons alone. Even contig-level draft assemblies of diverged genomes are helpful for reconstructing chromosome-scale sequences. CSA is also capable of assembling ultra-long reads.

**Conclusions:**

CSA can speed up and simplify chromosome-level assembly and significantly lower costs of large-scale family-level vertebrate genome projects.

## Background

### Whole-genome shotgun assembly in vertebrates—state of the art

Whole-genome shotgun (WGS) assembly of large vertebrate genomes has been an important topic of bioinformatics research over the past 2 decades, but obtaining completely assembled chromosomes through a single bioinformatics tool has not yet been achieved for large vertebrate genomes. Despite the ongoing replacement of short- by long-read sequencing in *de novo* genome projects, chromosome-level assemblies for vertebrates still require great bioinformatics expertise, especially in projects where cutting-edge genome maps or scaffolding data are not available.

Today, most vertebrate genomes can be assembled using noisy long reads [1–3] and the results—in terms of assembly contiguity, measured as contig N50—can outperform results obtained by short-read sequencing >100×. The contig N50 of today's noisy long-read assemblies reaches lengths similar to the scaffold N50 of high-quality short-read genome assemblies obtained some years ago. Still, current assembly tools can profit from their ancestors [4–9]. So far, most of them produce only contigs [10–15] and do not incorporate additional information to order these contigs into scaffolds, which would enable further gap closing and lead to chromosomal-level assemblies.

Chromosomal-level genome assembly, as the final goal of genome projects, still requires additional scaffolding or mapping data (Hi-C [16–18] or optical mapping [19], high-density genetic linkage map [20]), resulting in additional efforts that may add significant human, time, and financial resources to sequencing projects. For many, especially rare species, DNA resources for *de novo* genome sequencing come from archival tissues (e.g., frozen or ethanol-fixed or preserved in other storage media), preventing the application of Hi-C, which often requires living cells, and thus mapping panels can hardly be established. In such and similar cases, synteny and gene order analysis between evolutionary related genomes may be the only option to improve the genome assembly process.

### Synteny as a common feature of vertebrate genomes

All vertebrates, with currently ∼71,000 scientifically described species (August 2019), experienced 2 ancestral whole-genome duplications (WGDs), leading to ∼38,000 extant tetrapods, while most of the ~33,000 teleosts have gone through a third WGD [21, 22]. Beyond different ancestral WGD “substrates” that influenced the evolution of deletions, silencing and/or pseudogenization, sub- and neofunctionalization, genome size in vertebrates differs strongly (typical size range 0.4–4 Gb; in this regard the huge genome sizes of amphibians pose strong exceptions: C-value (haploid genome size) 3.3–57 pg [23]). While associated with neither morphological complexity nor gene numbers, genome size differences are caused by quantities of various repetitive elements and other non-coding DNA, composing up to 98% of vertebrate genomes [24].

Despite these WGD and size differences, “structural conservation” of vertebrate genomes as inferred from the distribution and positioning of genes on chromosomes, known as synteny, is a major feature of their evolution [25], with a pattern of conserved syntenic associations dating back 360 million years (My) [26], and even 600 My in other metazoans [27, 28]. Locations and order of genes (also referred to as “blocs”) in genomes depend on phylogenetic relatedness and on the “substrates” evolved after the ancient WGDs. Despite synteny, the various classes of vertebrates show different speed of chromosomal and sequence, and thus genome, evolution. Teleost fishes exhibit an accelerated evolutionary rate of protein-coding and other sequences, a higher rate of intron turnover, loss of many potential *cis*-regulatory elements, and shorter conserved syntenic blocks [29, 30]. Owing to their mostly enormous genome size with huge repetitive fractions, only 26 amphibian genomes have been sequenced and few reached chromosomal-scale quality [31], with deep divergences (often >100 My) between systematic amphibian families posing additional challenges. Nevertheless, ortholog genes that exhibit distinct order in bird chromosomes are also discretely ordered in the 2 assembled urodelans (*Ambystoma mexicanum, Notophthalmus viridescens*) [32–34] and the few anuran genomes [31, 35, 36], suggesting that ancestral chromosome segments and structures also remained conserved during amphibian phylogenesis [25]. Conservation of chromosomes, syntenic with avian autosomes, has been demonstrated in squamate reptiles [37], in which numerous microchromosomes pose special challenges for genomics [38]. Whole-genome comparisons among birds and mammals point to genomic regions where the orthologous gene order has been maintained for tens of millions of years [39, 40].

In summary, despite specific genomic properties of various vertebrate classes, synteny and conserved gene order present common and long-known inherent features of vertebrate genomes [41] that deserve to be better considered during genome assembly and that can be exploited by current bioinformatics.

### Exploiting synteny information for new approaches in vertebrate genomics

Indeed, evolutionary relationships such as highly conserved chromosome structure (synteny and gene order) in related vertebrate species [42], in some taxa even between several taxonomic levels [43], can enable low-cost approximations of chromosomal-scale assembly by comparative genomics [44–47]. An example for a successful short-read application is the genome assembly pipeline IMAP [48]. Of course, such approaches require the existence of ≥1 suitable high-quality reference genome, which has become a dwindling problem because for each vertebrate order ≥1 “platinum grade” reference genome will soon be created in Phase I of the international Vertebrate Genome Project (VGP, an offspring of the Genome10K Project [49]) and more will follow in the course of other large-scale genomics projects, such as the Earth BioGenome Project [50].

Here, we present a novel bioinformatics pipeline, which we call “Chromosome-Scale Assembler” (CSA). CSA overcomes limitations of current long-read assemblers by integrating comparisons between diverged reference genomes and/or scaffolds, derived from optical mapping, Hi-C, or 10X Genomics into the *de novo* assembly process. CSA runs computationally highly efficient tools for long-read genome assembly, whole-genome alignments, and reference-assisted chromosomal assembly in an iterative fashion. We show that CSA is able to produce chromosomal-level assemblies for smaller vertebrate genomes (fishes, birds) within 12 h on low-cost computing equipment ($1,000–2,000, Intel i7, 128 GB RAM), using just long-read data and a diverged reference genome (divergence time ∼65 million years ago [Mya]) as input. Larger mammalian genomes, such as human, can be assembled within 16 h on server equipment (Intel Xeon, 1 TB RAM). Depending on the type and coverage of the input data, CSA is able to improve contig N50 length up to 4.5-fold from initial to final contig assembly.

## Results and Discussion

### Implementation of the CSA pipeline

**Figure 1:**
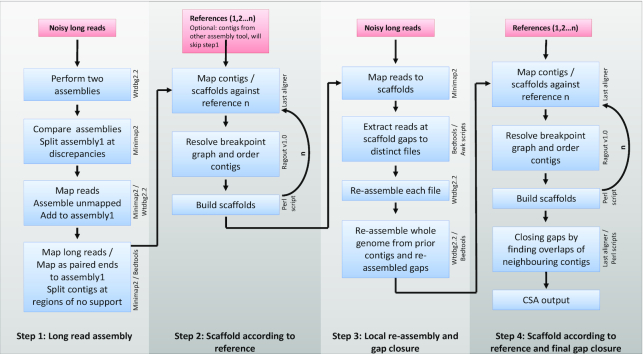
Flow chart of the 4-step CSA pipeline.

The first step of CSA (Fig. 1) is a *de novo* assembly of noisy long-read data (either Pacific Biosciences [PacBio] or Oxford Nanopore data). It uses the WTDBG2 (version 2.2, 11 December 2018) assembler because it is among the most computationally efficient *de novo* genome assemblers to date and according to Ruan and Li [14] is 2–17 times as fast as its closest competitor. CSA runs 2 WTDBG assemblies with slightly varying parameters and splits the contigs at discrepancies between both assemblies to get rid of rarely occurring misassemblies. Additionally, we re-assemble long reads that can only be partially mapped to the WTDBG2 assembly (<10% of read length); this step may recover up to 1–2% of genomic sequence (large contigs) that is missing in current WTDBG2 primary assemblies. A final assembly curation is performed by re-mapping (MINIMAP2) [51] long reads and paired ends of long reads (500 bp from each end) to the assembly and split contigs at regions of zero coverage. If needed, the WTDBG2 assembly can be omitted and a contig file from another genome assembly tool can be used by CSA; thus, CSA can also be used to update existing assemblies.

In Step 2, the resulting curated contigs are mapped by LAST aligner [52] to 1 or more references. These references may be scaffolds from the same species that have been built using various methods (e.g., 10X Genomics, optical mapping, or Hi-C). An outstanding feature of CSA is that even diverged reference and draft genomes are a suitable input. The LAST alignments are used by RAGOUT [53] to order the curated contigs (from step 1) into scaffolds, which then already may reach chromosomal size.

During Step 3 all noisy long reads are mapped to the scaffolds by MINIMAP2. Reads that map in 20-kb windows around scaffold gaps or contig ends are extracted into distinct fasta files. These files are submitted to the WTDBG2 assembler and are locally re-assembled in parallel. The resulting local re-assemblies for each gap/contig end are then assembled with the primary WTDBG2 contigs that have been split into overlapping pseudo-reads to meet the read length limits of WTDBG2 (256 kb in version 2.2; version 2.4 has no limits but showed lower performance in tests). Because WTDBG2 now assembles pre-assembled reads with higher accuracy (consensus accuracy 98–99%), more stringent parameters are set. This iterative assembly step can typically double N50 contig sizes as shown in different tests hereafter. Alignment of the improved contigs to the prior scaffolds is used to remove the few intra- and inter-scaffold misassemblies.

Step 4, again, maps the improved contigs against the references used in Step 2 by LAST aligner and runs RAGOUT to order the contigs into scaffolds. Finally, some gaps with overlapping neighbouring contig-ends are identified by LAST and closed.

The tools selected to build the CSA pipeline have been mainly chosen on the basis of performance and sensitivity when using diverged reference genomes. Relatively simple procedures split primary *de novo* assemblies at regions with low support (e.g., non-continuously covered regions in comparisons of 2 *de novo* assemblies or regions of low read coverage), which is relatively fast, even when dealing with very large genomes. For assembly reconciliation, more sophisticated tools have been developed, e.g., SMSC [54] or BIGMAC [55], but these have been rarely tested on large genomes and, according to published benchmarks on small-sized genomes (bacteria, yeast), these modules might take longer than the entire CSA pipeline. Nevertheless, improved faster detection of misassemblies by read re-mapping could further improve assembly quality in future versions of CSA.

Another well-tuned example seems our choice of RAGOUT, which can be easily adapted to the maf format output of the fast and sensitive LAST aligner, while similar tools like MeDuSa [56] and RACA [44] use computationally more expensive (LASTZ in RACA) or less sensitive aligners that do not work well on diverged reference genomes (MUMMER [57] in MeDuSa). RAGOUT1 [53] was preferred over RAGOUT2 [45], because it resulted in slightly better chromosome assemblies. RAGOUT can also close gaps, but this feature was designed for short, error-free overlaps of contigs from short-read assemblies and is not efficiently working with long-read assemblies. Thus, we implemented our own solutions for local gap reassembly and final contig stitching. In principle, gap closing could also be done by tools such as PBJelly [58] or LR_gapcloser [59], but we found that some closable gaps remain unclosed by these tools, probably due to overlapping repeat sequences at some contig ends, which do occur even in long-read assemblies.

The current version of CSA does not include methods for consensus polishing, yet, a single iteration of consensus polishing using long reads and 2 iterations using short reads must be applied to obtain high-quality consensus sequence and prior to sequence annotation efforts. We have had good experience using MEDAKA (by Oxford Nanopore Technologies [ONT] [60]) and PILON in this regard (benchmark Scenario 3). Recently we have replaced MEDAKA by FLYE-POLISH [12] because it is able to use both SMRT (single molecule real time; PacBio) and ONT data.

In the following, we tested CSA on different scenarios and benchmark its performance. CSA automatically chains many steps that traditionally require different software tools and laborious manual curation, for which similar pipelines are currently only available for short-read assembly (IMAP [48]). Therefore, we do not compare CSA results with known contig-level genome assembly tools or other software packages solving only parts of chromosomal assemblies but by re-assembling the currently best chromosome-scale reference genomes in different vertebrate species.

### Benchmark Scenario 1: Updating existing fish, bird, and mammal assemblies, using a prior assembly version as reference

The current CSA pipeline was tested using SMRT long-read sequencing data for representative species of 3 different vertebrate clades, namely, Mammalia (*Homo sapiens* [Hs]), Aves (*Taeniopygia guttata* [Tg]), and Teleostei (*Siniperca chuatsi* [Sc]). Our first tests used high-quality genomes of the same species from which the long-read input data were derived to assist the assembly. These tests show what we can expect from CSA in a best-case scenario. In a real-world scenario, where no known reference of the same species is available, this approach would be comparable to using CSA and scaffolding the CSA Step 1 assembly by Hi-C data and then continuing with assembly improvements (CSA Steps 2–4). The detailed results of these benchmarks are shown in Supplementary Table S1 .

In terms of chromosomal assembly completeness, we measured how much of the consensus sequence is contained in the top n largest scaffolds, where n is the haploid chromosome number. All CSA assemblies placed >94% of the consensus into the top n scaffolds (Hs = 97.5%; Tg = 94.2%; Sc = 99.3%). The contig N50 length was 25.9, 27.7, and 16.5 Mb for Hs, Tg, and Sc, respectively. These values outperform the current reference contig N50 for Tg (VGP assembly) and Sc (our own results), which are based on the same long-read input data but included different genome maps and curation steps to improve the assembly. For Hs we compared contig N50 to the so far best assembly from PacBio (Accession: GCA_003634875.1) data and found that CSA produced similar values, although we used an older dataset (P4C6 chemistry from RSII sequencer) for our tests. For Tg, we could improve contig N50 by 2.3-fold over the VGP assembly. The contig N50 of Sc improved 1.35-fold over our sinChu7 assembly.

**Figure 2:**
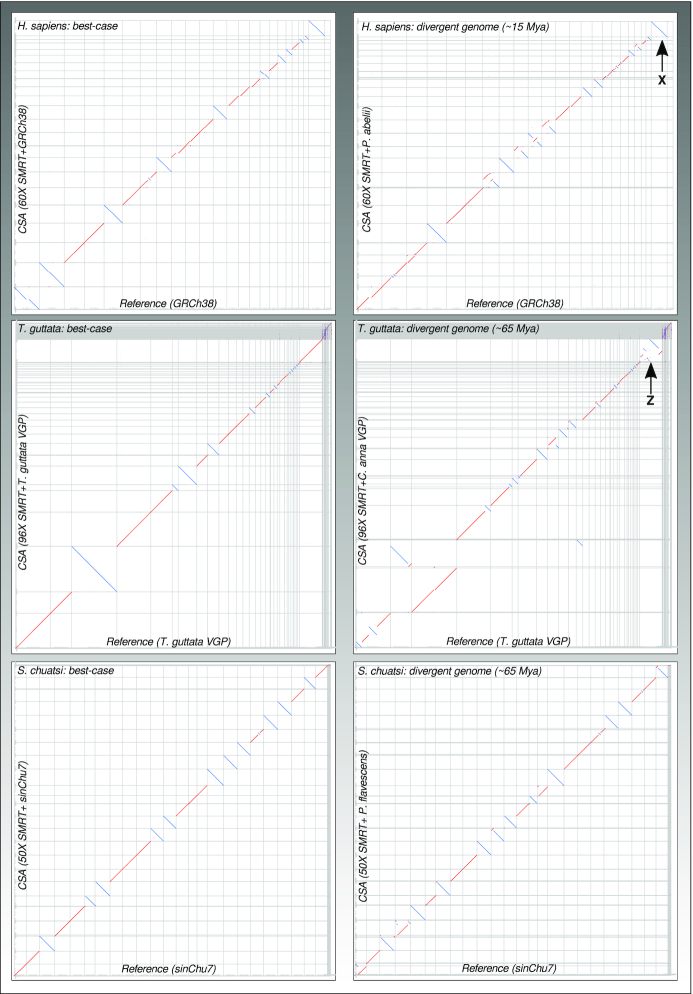
Dot plots of CSA results against reference genomes under best-case (left) and diverged reference scenarios (right) for mammal, bird, and fish genomes. Thin vertical lines separate chromosomes of the reference assembly; thin horizontal lines separate CSA scaffolds. Red and blue colours depict forward or reverse orientation of the alignments. Lines that are not placed on diagonals or sub-diagonals indicate rearrangements between reference and CSA assemblies. Note that a single blue line match per reference chromosome does not mean a large inversion is present but that CSA just outputted the corresponding chromosomal scaffold as reverse complement orientation. X chromosomes in *H. sapiens* and Z chromosomes in *T. guttata* are marked in the plots. In *T. guttata*, the Z chromosome shows a higher number of rearrangements than the autosomes.

Finally, we compared CSA assemblies versus the references to visually inspect assembly errors by dot plots (Fig. 2 left) and counted larger-scale synteny (gene order) breaks (rearranged genomic blocks >300 kb) by custom scripts. The CSA assemblies exhibited only a few structural misassemblies (f = interchr. Fusion/fission, t = intrachr. translocation, i = inversion: Hs: f: 0; t: 6; i: 2/Tg: f: 0; t: 1; i: 4/Sc: f: 0; t: 1; i: 5). For the teleost assembly, CSA even polished 2 misassemblies in the current reference genome (1 fusion and 1 inversion).

These results show that under our best-case scenario the pipeline performed very well and CSA seems to be a valuable tool to improve existing reference genomes by complete re-assembly as soon as improved sequence data are available.

### Benchmark Scenario 2: CSA using divergent genomes as reference allows chromosomal-scale assemblies from long reads only

Although this approach can be limited by complex evolutionary scenarios involving major rearrangements of genomes, in principle, the mapping steps in CSA have been designed to allow for incorporation of highly diverged genomes as references. Nowadays (and in the future even more) one will find suitable, perhaps distantly related reference genomes for most vertebrate species in databases, and this gives us the opportunity to obtain high-quality chromosome-scale assemblies from long-read data alone—potentially even without having other mapping data at hand (e.g., Hi-C, optical maps, linkage maps). We tested CSA on the long-read data from above using high-quality reference genomes of species that diverged between 10 and 240 Mya as references. Representative CSA assemblies, using diverged references, are listed in Table 1 and compared to state-of-the-art reference assemblies; the detailed results for all benchmarks are presented in Supplementary Table S2.

**Table 1:** CSA enables chromosomal-scale genome assemblies of mammal, bird, and fish by using just long-read data and a diverged reference genome

Assembly	Assembly strategy	Total assembled length without gaps (bp)	Contig N50 length	% bp placed in top n scaffolds	Structural discrepancies vs reference		Assembly time frame
					In scaffolds	In contigs	
*Homo sapiens*							
GRCh38 [Reference]	Clone based, extensive manual curation	2,937,639,113	50,761,348	98.64% (n = 23)			Years
GCA_003634875.1	SMRT + Hi-C	2,893,114,812	26,292,878	97.82% (n = 23)	f: 25; t: 58; i: 14	f: 5; t: 0; i: 4	Days/weeks
CSA	SMRT + diverged genome 10–20 Mya (*P. abelii*)	2,849,099,702	29,334,513	93.80% (n = 23)	f: 0; t: 58; i: 8	f: 0; t: 4; i: 6	Hours/days
CSA (see benchmark Scenario 6)	ONT ULRs + diverged genome 10–20 Mya (*P. abelii*)	2,884,341,430	45,943,940	92.90% (n = 23)	f: 2; t: 53; i: 8	f: 1; t: 5; i: 6	Hours/days
*Taeniopygia guttata*							
VGP [Reference]	SMRT + Optical map + Hi-C + curation	1,054,772,052	11,998,827	98.98% (n = 40)			Days/weeks
CSA	SMRT + diverged genome 65 Mya (*C. anna*)	1,096,370,479	18,882,724	92.13% (n = 40)	f: 1; t: 33; i: 20	f: 0; t: 1; i: 6	Hours
*Siniperca chuatsi*							
sinChu7 [Reference]	SMRT + high-density linkage map + curation	753,983,610	12,191,788	96.68% (n = 24)			Days/weeks
CSA	SMRT + diverged genome 65 Mya (*P. flavescens*)	721,014,191	16,688,192	94.73% (n = 24)	f: 1; t: 13; i: 16	f: 0; t: 0; i: 5	Hours
*Perca fluviatilis*							
PFLU1.1 [Reference]	ONT + Hi-C + curation	950,435,818	2,593,362	99.00% (n = 24)			Days/weeks
CSA	ONT + diverged genomes 10–20 and 65 Mya (*P. flavescens*+ *S. chuatsi*)	928,809,152	7,745,610	93.79% (n = 24)	f: 0; t: 19; i: 15	f: 0; t: 0; i: 3	Hours

In most cases, CSA improved the contig N50 length over current state-of-the-art reference genomes. Completeness of chromosomal assembly was in the range of 92.1–94.7%, only slightly below what can be obtained by using Hi-C data (97.8–99.0%). Structural discrepancies (>300 kb) with the reference genomes were low, especially for the contig level and, in case of the human genome, even lower than a comparable assembly, which used Hi-C mapping (GCA_003634875.1). f: interchromosome fusion/fission; i: inversion; SMRT: single-molecule real-time sequencing; t: intrachrosome translocation; ULR: ultra-long read.

Overall, the fraction of consensus sequences assigned to the top n scaffolds was slightly lower than under the best-case scenario, but it was well above 90% for the less diverged references. The loss of placed sequence typically occurs in the subtelomeric regions that diverge faster than the other chromosomal regions. In most cases, using more diverged reference genomes, CSA still allowed the placement of >92% of the assembly in the top n scaffolds.

Improvements of contig N50 were still observed at a similar scale as in the best-case scenario, and introduced assembly errors due to divergent reference genomes were low on the contig level (Supplementary Table S2, row “Errors ctg”). Our main focus of this benchmark was to analyse large-scale misassemblies that are introduced by using diverged genomes as references in the chromosomal scaffold assembly (Fig. 2*right*, additional plots Supplementary Figs S1 and S2) and how these develop with increasing divergence time. As expected, here we saw clear differences between mammals, birds, and teleosts.

Chromosomal gene order is highly conserved in birds [43], and among vertebrates, bird genomes have the lowest fraction of repetitive sequences (<20%) [61]. This possibly explains why CSA works very well for most autosomes when using diverged bird genomes (up to 90 Mya) as reference. Nevertheless, here we found a few chromosomal fusion errors that were related to known differences in bird karyotypes (e.g., fusion/fission of chr1/chr1A; chr4/chr4A) and a clear enrichment of inversion and translocation errors on the Z-chromosome (*Gallus gallus*: 32% and *Calypte anna*: 35% of t and i errors on Z), possibly a result of fast evolution of the Z/W sex chromosomes, which has been described earlier [62]. Error profiles were f: 1; t: 33; i: 20 and f: 2; t: 26; i: 20 when using *C. anna* (∼65 Mya) and *G. gallus* (∼90 Mya) as reference, respectively. Finally, CSA still worked reasonably well using the scaffold-level *Alligator mississipiensis* draft genome as reference, which diverged ∼240 Mya (f: 2; t: 22; i: 18).

In mammals, assembly errors were distributed more evenly over autosomes and for the X-chromosome we did not find an enrichment of errors, like in the bird Z chromosome. The 3-times larger and more repetitive (>30%) mammal genomes [61] were more prone to misassemblies with increasing divergence time of the reference than bird genomes. Still, CSA results were good for references that diverged 10–20 Mya (in our example *Pongo abelii:* f: 0; t: 58; i: 8).

In teleosts, we did not observe chromosome-specific assembly issues and the increase of misassemblies with divergence of the reference was not as harsh as in mammals, possibly due to the more compact genomes of most teleosts. Thus, CSA performed well when using fish reference genomes with divergence times <65 Mya (here *Perca flavescens*: f: 1; t: 13; i: 16).

Our results show that long-read data and well-chosen, order-level state-of-the-art reference genomes enable CSA to calculate highly continuous assemblies for most chromosomes, but in some cases clade-specific problems have to be resolved by manual curation. As a rule of thumb, if choosing from reference genomes that have similar divergence times to support assembly of a new genome, those with the same haploid chromosome number and slowest evolution (as depicted by small branch length in phylogenetic trees or a high fraction of alignable sequence between assembled and reference genome) should be preferred. Nevertheless, considering the contig level, the use of distant genomes as references in CSA is quite safe and produces only a few errors (Supplementary Table 2, compare “Errors scf” against “Errors ctg”) but still is able to highly improve contig N50.

### Benchmark Scenario 3: CSA on a fish genome, integrating ONT reads, 10X Genomics scaffolds, and diverged reference genomes

The previous benchmarks used SMRT long-read data and a single reference genome. In the following, we ran CSA using long reads generated by ONT sequencing from genomic DNA of *Perca fluviatilis* and supported the assembly by a 10X Genomics assembly of the same species and 2 diverged reference genomes.

A high-quality chromosomal-scale genome assembly of *P. fluviatilis* assembled from the same ONT long reads, and Hi-C sequencing has recently become available (BioProject Accession: PRJNA549142, the assembly has been updated recently, note that the reference used here was an earlier version called PFLU1.1 and is available from the supporting data [71]). The high-quality draft genome assembled from 10X Genomics sequence data was published earlier (68× Illumina short-read coverage, N50 contig/scaffold length 18.3 kb/6.3 Mb according to [63]). A chromosomal-scale reference genome for *P. flavescens*, a close relative (genus-level) of *P. fluviatilis*, is available [64]; both *Perca* diverged ∼8.2–17.5 Mya. As a more distantly related reference (divergence time ∼65 Mya), we used the chromosomal-scale *Siniperca chuatsi* genome (BioProject Accession: PRJNA513951).

To test whether CSA could reach a similar assembly quality as for the *P. fluviatilis* reference genome, we ran CSA on *P. fluviatilis* ONT long reads, supported by 10X Genomics scaffolds and *P. flavescens* and *S. chuatsi* genomes (see Supplementary Table S3).

**Figure 3:**
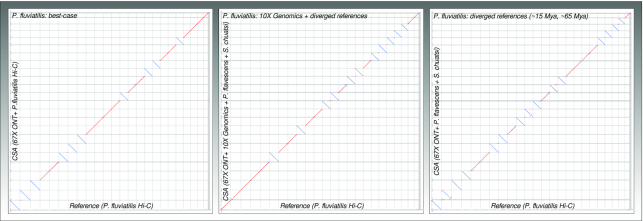
Dot plots of CSA results against reference genome for assembly of *Perca fluviatilis*—Oxford Nanopore data, sequentially supported by 10X Genomics and 2 diverged references genomes. For explanation of the dot plot properties, see Figure 2.

CSA managed to place 94.3% of the assembled sequence into 24 large scaffolds, which corresponded to the reference chromosomes. Only a few differences (f: 0; t: 12; i: 13) in chromosomal structure were apparent in the dot plot (Fig. 3). CSA increased the contig N50 nearly 3.1-fold compared to the reference genome. The total time to complete the chromosomal assembly was 5h:30 min, when using 80 CPU threads on a high-performance computing server or 12 h on a high-end desktop computer (12 CPU threads, 128 GB RAM). We polished the CSA assembly using MEDAKA (by ONT [60]) and PILON (RRID:SCR_014731) [65] and performed BUSCO (RRID:SCR_015008) [66], which confirmed that the assembly was highly complete on the gene level (Actinopterygii dataset, complete genes: 95.9%, fragmented genes: 2.1%, missing genes: 2.0%, number of tested genes: 4,584).

Next, we performed the same runs and omitted the 10X Genomics scaffold data. This only resulted in a slight loss of sequences placed in chromosomes (now 93.8%; loss 0.5%) and a few more intrachromosmal translocations and inversions (f: 0; t: 19; i: 15).

Finally, using only the most diverged reference still resulted in 86.3% of sequence placed in the top 24 scaffolds, but more intra-chromosomal translocations (f: 0; t: 57; i: 16). Yet, improvement of contig N50 was still 2.7-fold and structural errors in contigs were low (f: 0; t: 0; i: 4).

Thus, CSA was able to compute chromosome-scale assemblies by sequentially using species-, genus- and order-level references together with ONT long-read data. The species-level reference (10X Genomics, Supernova assembly) was only slightly contributing to the final assembly owing to its lower N50 scaffold length of 6.3 Mb. We have observed that ONT long-read datasets of comparable N50 read length and coverage produce less contiguous assemblies than SMRT datasets, possibly due to coverage bias of genomic sequences that interfere with ONT sequencing. According to our results the 2 gap closure steps performed by CSA were highly efficient to improve contig N50 in such a situation.

### Benchmark Scenario 4: CSA using draft assemblies as reference; contig-level assemblies of diverged species may be highly complementary

Under Scenario 2 we already found that draft assemblies of other species could be used to improve genome assemblies (*T. guttata*/*A. mississipiensis* results). So we asked the question, whether a diverged, low-N50 contig-level assembly could still support CSA to result in improved assemblies.

**Figure 4:**
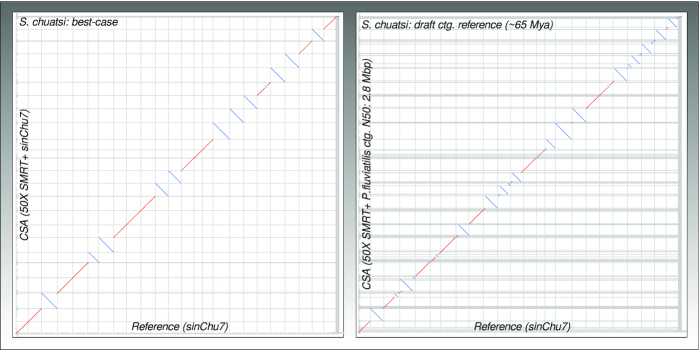
Dot plots of CSA results against reference genome for assembly of *Siniperca chuatsi* supported by contigs of a diverged draft genome assembly in comparison to the best-case CSA assembly. For explanation of the dot plot properties, see Figure 2.

Thus, we assembled the *S. chuatsi* genome using *P. fluviatilis* contigs (from Scenario 3 CSA step 1: N50 = 2.8 Mb) as reference. Although the reference contig N50 was relatively low, it was improving the *S. chuatsi* assembly significantly (Supplementary Table S2 last column). The *S. chuatsi* assembly continuity doubled from an N50 of 11.6 Mb (primary contigs) to 23.4 Mb (final scaffolds). The top 24 scaffolds consisted of 77.3% and the top 48 scaffolds consisted of 89.9% of the assembled sequence; thus, chromosomal assembly was less complete (Fig. 4). Interestingly, the improvement of contig N50 (1.37-fold) due to gap closure was similar to the tests performed with high-quality reference genomes in Scenario 2 and the number of assembly errors was low (scaffolds: f: 0; t: 2; i: 9/contigs: f: 0; t: 0; i: 5).

Thus, CSA is able to use even low continuous contig assemblies of diverged species to improve genome assemblies. This opens up new strategies in projects where many species of a certain clade are sequenced and might complement the assemblies of each other already at the draft state.

### Benchmark Scenario 5: Benchmarking influence of long-read sequencing coverage

**Figure 5:**
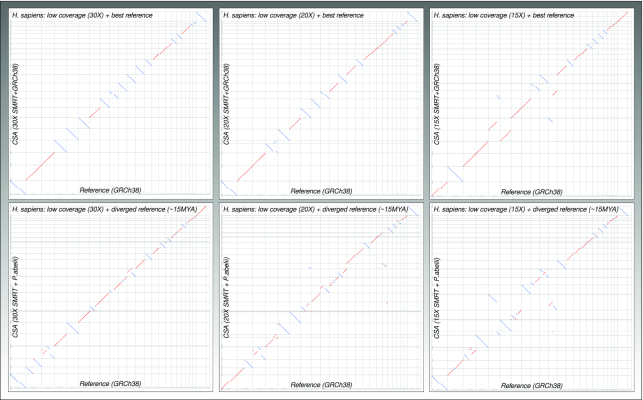
Dot plots of CSA results against reference genome for reduced coverage data (*Homo sapiens*), using either the best reference or a diverged reference to support CSA. For explanation of the dot plot properties, see Fig. 2.

On primary assemblies of lower contig N50 length, CSA can play its strength in gap closure. As this was already observed in Scenario 3, we now asked the question how long-read sequencing coverage influences the results of CSA assembly. We randomly subsampled reads from the *H. sapiens* 60× SMRT sequencing dataset, to obtain subsets of 15×, 20×, 30×, and 40× sequencing coverage. We observed only slight changes of the final results for 60×, 40×, and 30× sequencing coverage. Although contig N50 of the primary assembly started to drop below 30×, the CSA gap closures in Steps 3 and 4 still enabled a final contig N50, similar to what was obtained from the 40× and 60× datasets (Supplementary Table S4). The 20× and 15× data had significantly lower contig N50; here the improvement by the CSA gap closure was clearly the highest (3.8-fold for 20× and 4.5-fold for 15×), but assembly errors (especially fusion errors) started to increase (Fig. 5). Similar results were observed when the diverged *P. abelii* genome was used as reference. It seems worthwhile to mention that contig N50 length of the primary assembly (CSA Step 1) is an important factor and should be at least in the megabase range because low contiguity of the contigs increases the chance of wrongly resolving rearrangements between query and reference genomes.

Thus, when running CSA, 30× sequencing coverage is sufficient and even lower coverage may lead to respectable results. Particularly, low-coverage assemblies take profit from gap closure steps, and CSA can improve contig N50s by several hundred percent.

### Benchmark Scenario 6: Ultra long-read assembly

Ultra-long reads (ULR, N50 read length >50 kb) are currently gaining importance in the sequencing community and will possibly be available to many researchers soon. CSA default parameters have been optimized for current long-read data (N50 read length <30 kb). It has been reported recently that WTDBG2 performs relatively poorly on ULR data compared with the SHASTA assembler, which was designed for ULR assembly [67]. We found that optimization of some parameters of WTDBG2 overcame these issues (increasing minimum read length cut-off to roughly N50 read length, while maintaining sequence coverage >25× and increasing minimum overlap cut-off to ∼30% of the N50 read length). We found that CSA was running more slowly due to its several read re-mapping steps, which are computationally less efficient when using ULR data. Still, the assembly finished within 24 h on our compute-server. Our CSA ULR assembly (best case, using GRCh38 as reference; details: Supplementary Table S5) did compete well in terms of contig N50 (48.4 vs 46.0 Mb) with the SHASTA assembler, producing significantly fewer contigs (1,526 vs 1,925) and more complete total consensus length (2.9 vs 2.8 Mb, before sequence polishing). The number of structural misassemblies in CSA scaffolds, if compared against GRCh38 human reference chromosomes, was similar to using SMRT reads in Scenario 1 (f: 0; t: 9; i: 2, dot plot in Supplementary Fig. 1). On the contig level, we could compare CSA and SHASTA assemblies, which were both nearly free of large structural errors (CSA: f: 0; t: 0; i: 2/SHASTA: f: 0; t: 0; i: 0). This picture only slightly changed if we used a diverged reference during the CSA assembly (Table 1, Supplementary Table S5 and Fig. S3). In this case, the contig N50 of SHASTA and CSA were similar (both ∼46.0 Mb) and CSA error rates were slightly higher (scaffold level: f: 2; t: 53; i: 8/contig level: f: 1; t: 5; i: 6).

## Conclusions

Considering the scenarios tested, we have shown that CSA is a reliable tool that goes far beyond the contig-level assembly of long reads and enables automated chromosome-scale assemblies. Nevertheless, well-known assembly issues, such as genomes exhibiting high heterozygosity, higher ploidies, or extreme repeat content and genome size, may still result in assemblies of lower contiguity. For example, the few available high-quality amphibian genomes are currently posing challenges to CSA for this vertebrate class, as long as no high-quality Hi-C scaffolds from at least a relatively closely related species are available to support the assembly (model genomes from each systematic amphibian family would amount to great progress).

Yet, considering mammals, birds, fishes, and possibly reptiles, CSA allows for lower sequencing coverage in genome projects and reduces the need for computational resources. Thus, CSA can contribute to save significant human, time, and financial resources and thus reduce costs in small- and large-scale genome projects. Furthermore, CSA enables beginners to genome assembly to perform chromosomal-level assemblies, even on datasets that would be considered suboptimal when using other assembly tools. We are confident that CSA presents another important step towards the democratization of genome sequencing and assembly.

## Methods

### CSA GitHub project

CSA2.6 and future updates can be downloaded from [68]. All tools needed to run the pipeline will be installed by a script in the folder “CSA2.6/INSTALL”. Simply run “bash INSTALL.bash” and follow the instructions. Some system-specific installation issues are mentioned on GitHub. We have tested CSA2.6 on fresh server installations of Red Hat 8 and Ubuntu 18.04/19.04, OpenSuse LEAP 15.1, and CentOS 7 as well as older Red Hat and Ubuntu versions.

Owing to ongoing development of the CSA pipeline we provide the code that has been used to benchmark Scenarios 1–5 and 6 (see Additional Files 3 and 4 in the Supplementary Material).

CSA default parameters are currently tweaked for PacBio RSII and ONT reads (30–60×, N50 read length 10–30 kb). We have found that some SEQUEL datasets behave quite differently; here adding custom parameters for WTDBG2 will help: -l “-p 0 -k 15 -L5000 -S 2 -A.”

### Benchmark Scenario 1—Data and CSA parameters

For the best-case scenario we downloaded reference genomes for *H. sapiens* (GRCh38.p12; RefSeq assembly accession: GCF_000001405.38, here we kept only the chromosomes and removed alternative loci) and *T. guttata* (bTaeGut1_v1.p; RefSeq assembly accession: GCF_003957565.1). For *S. chuatsi* we used our new reference genome sinChu7 (BioProject accession: PRJNA513951). SMRT long-read data for *H. sapiens* were downloaded from the SRA accession SRP044331. SMRT long-read data for *T. guttata* were downloaded from SRA using the accessions: SRR5224495–SRR5224503. The SMRT data for *S. chuatsi i*s available through the BioProject accession PRJNA513951. All SMRT data were selected for longest subreads and converted to gzip compressed fasta files.

CSA assemblies were run by the following commands:

CSA2.6c.pl -r homSap_longest_subreads.fa.gz -g \GRCh38.p12.CHR.fa.gz -t 80 -d HS-GRCh38-2_6C\

-o HS-GRCh38-2_6C > HS-GRCh38-2_6C.bash

nohup bash\

HS-GRCh38-2_6C.bash > HS-GRCh38-2_6C.log 2>&1 &

 

CSA2.6c.pl -r taeGut_SMRT.fa.gz -g\

bTaeGut1_v1.p.fasta.gz -t 80 -o TG-TG-VGP-2_6C\

-d TG-TG-VGP-2_6C > TG-TG-VGP-2_6C.bash

nohup bash\

TG-TG-VGP-2_6C.bash > TG-TG-VGP-2_6C.log 2>&1 &

 

CSA2.6c.pl -r PACBIO-READS-RAW.fa.gz -g\

sinChu7.fasta -o SC-SC-2_6C\

-d SC-SC-2_6C -t 80 > SC-SC-2_6C.bash

bash SC-SC-2_6C.bash > SC-SC-2_6C.log 2>&1 &

### Benchmark Scenario 2—Data and CSA parameters

For our diverged reference scenario we downloaded the following genome assemblies.

**Mammals:** *P. abelii* (Accession: GCF_002880775.1); *C. jacchus* (Accession: GCA_002754865.1); *L. canadensis* (Accession: GCF_007474595.1); *O. anatinus* (Accession: GCF_004115215.1).

**Birds:** *C. anna* (Accession: GCF_003957555.1); *G. gallus* (Accession: GCF_000002315.6).

**Reptile:** *A. mississipiensis* (Accession: GCF_000281125.3).

**Fish:** *P. flavescens* (Accession: GCF_004354835.1).

CSA assemblies were run as above but omitting the primary assembly step. Because the primary assemblies were already calculated under Scenario 1 (CSA Step 1 is a pure *de novo* assembly without support by reference), we can just add the fasta contigs using the parameter –C to save computing time (this procedure would also allow using primary assemblies from other assembly tools than WTDBG2):

#### H. sapiens

CSA2.6c.pl -C HS-GRCh38-2_6C.step1.fa -r\

homSap_longest_subreads.fa.gz\

-g GCF_002880775.1_Susie_PABv2_genomic.fna.gz -t 80\

-d HS-PA-2_6C -o HS-PA-2_6C > HS-PA-2_6C.bash

nohup bash HS-PA-2_6C.bash > HS-PA-2_6C.log 2>&1 &

 

CSA2.6c.pl -C HS-GRCh38-2_6C.step1.fa -r\

homSap_longest_subreads.fa.gz\

-g GCA_002754865.1_ASM275486v1_genomic.fna.gz -t 80\

-d HS-CJ-2_6C -o HS-CJ-2_6C > HS-CJ-2_6C.bash

nohup bash HS-CJ-2_6C.bash > HS-CJ-2_6C.log 2>&1 &

 

CSA2.6c.pl -C HS-GRCh38-2_6C.step1.fa -r\

homSap_longest_subreads.fa.gz\

-g mLynCan4_s2.fasta.gz -t 80 -d HS-LC-2_6C -o\

HS-LC-2_6C > HS-LC-2_6C.bash

nohup bash HS-LC-2_6C.bash > HS-LC-2_6C.log 2>&1 &

 

CSA2.6c.pl -C HS-GRCh38-2_6C.step1.fa -r\

homSap_longest_subreads.fa.gz -g\

GCF_004115215.1_mOrnAna1.p.v1_genomic.fna.gz -t 80\

-d HS-OA-2_6C -o HS-OA-2_6C > HS-OA-2_6C.bash

nohup bash HS-OA-2_6C.bash > HS-OA-2_6C.log 2>&1 &

#### T. guttata

CSA2.6c.pl -C TG-TG-VGP-2_6C.step1.fa -r\

taeGut_SMRT.fa.gz -g bCalAnn1_v1.p.fasta.gz\

-t 80 -o TG-CA-VGP-2_6C -d\

TG-CA-VGP-2_6C > TG-CA-VGP-2_6C.bash

nohup bash\

TG-CA-VGP-2_6C.bash > TG-CA-VGP-2_6C.log 2>&1 &

 

CSA2.6c.pl -C TG-TG-VGP-2_6C.step1.fa -r\

taeGut_SMRT.fa.gz\

-g GCF_000002315.6_GRCg6a_genomic.fna.gz -t 80 -o\

TG-GG-2_6C -d TG-GG-2_6C > TG-GG-2_6C.bash

nohup bash TG-GG-2_6C.bash > TG-GG-2_6C.log 2>&1 &

 

CSA2.6c.pl -C TG-TG-VGP-2_6C.step1.fa -r\

taeGut_SMRT.fa.gz -g\

GCF_000281125.3_ASM28112v4_genomic.fna.gz -t 80 -o\

TG-AM-2_6C -d TG-AM-2_6C > TG-AM-2_6C.bash

nohup bash TG-AM-2_6C.bash > TG-AM-2_6C.log 2>&1 &

#### S. chuatsi

CSA2.6c.pl -C SC-SC-2_6C.step1.fa -r\

PACBIO-READS-RAW.fa.gz\

-g GCF_004354835.1_PFLA_1.0_genomic.fna.gz\

-o SC-PFLA-2_6C\

-d SC-PFLA-2_6C -t 80 > SC-PFLA-2_6C.bash

nohup bash\

SC-PFLA-2_6C.bash > SC-PFLA-2_6C.log 2>&1 &

### Benchmark Scenario 3—Data and CSA parameters

To assemble the *Perca fluviatilis* genome by CSA, we used the *P. fluviatilis* reference genome for the best-case scenario (BioProject Accession: PRJNA549142, the assembly has been updated recently, note that the reference used here was an earlier version called PFLU1.1 and is available from the supporting data [71]). A 10X Genomics Supernova assembly (Accession: GCA_003412525.1) of *P. fluviatilis* as well as the *P. flavescens* and the *S. chuatsi* genomes from above were used to benchmark CSA using multiple references sequentially. Oxford Nanopore long-read data for *P. fluviatilis* were obtained from BioProject Accession PRJNA549142. CSA parameters for the best-case scenario were as follows:

CSA2.6c.pl -r perFlu_ONT_ALL.fa.gz\

-g Perca_fluviatilis.PFLU1.1.dna.toplevel.fa.gz\

-t 80 -o PF-PF-HiC-2_6C\

-d PF-PF-HiC-2_6C > PF-PF-HiC-2_6C.bash

nohup bash\

PF-PF-HiC-2_6C.bash > PF-PF-HiC-2_6C.log 2>&1 &

Again for the assembly using multiple references, we used the primary contigs from above (-C), now adding the reference sequences for sequential improvement as a comma separated list (e.g., -g closest.fa, less_diverged.fa, most_diverged.fa):

CSA2.6c.pl -C PF-PF-HiC-2_6C.step1.fa -r\

perFlu_ONT_ALL.fa.gz\

-g GCA_003412525.1_UTU_Pfluv_1.1_genomic.fna.gz,\

Perca_flavescens.PFLA1.1.dna.toplevel.fa.gz,\

sinChu7.fasta -t 80 -o PF-10X-PFLA-SC-2_6C\

-d PF-10X-PFLA-SC-2_6C > PF-10X-PFLA-SC-2_6C.bash

nohup bash\

PF-10X-PFLA-SC-2_6C.bash > PF-10X-PFLA-SC-2_6C.log 2>&1 &

 

CSA2.6c.pl -C PF-PF-HiC-2_6C.step1.fa -r\

perFlu_ONT_ALL.fa.gz\

-g Perca_flavescens.PFLA1.1.dna.toplevel.fa.gz,\

sinChu7.fasta -t 80 -o PF-PFLA-SC-2_6C\

-d PF-PFLA-SC-2_6C > PF-PFLA-SC-2_6C.bash

nohup bash\

PF-PFLA-SC-2_6C.bash > PF-PFLA-SC-2_6C.log 2>&1 &

CSA2.6c.pl -C\ PF-PF-HiC-2_6C/01_WTDBG/PF-PF-HiC-2_6C.step1.fa\

-r ../DATA/perFlu/perFlu_ONT_ALL.fa.gz\

-g ../REFERENCES/sinChu7.fasta\

-t 80 -o PF-SC-2_6C -d PF-SC-2_6C > PF-SC-2_6C.bash

nohup bash PF-SC-2_6C.bash > PF-SC-2_6C.log 2>&1

### Benchmark Scenario 4—Data and CSA parameters

Here we used the relatively low N50 contig length primary assembly of *P. fluviatilis* from Scenario 3 to assemble the *S. chuatsi* SMRT data from above:

CSA2.6c.pl -C SC-SC-2_6C.step1.fa -r\

PACBIO-READS-RAW.fa.gz -g PF-PF-HiC-2_6C.step1.fa\

-o SC-PFdraft-2_6C -d \

SC-PFdraft-2_6C -t 80 > SC-PFdraft-2_6C.bash

nohup bash SC-PFdraft-2_6C.bash > SC-PFdraft-2_6C.log 2>&1 &

### Benchmark Scenario 5—Data and CSA parameters

To get subsets of the *H. sapiens* SMRT data we used SEQTK to randomly subsample reads from the full dataset in a way that we obtained ∼40×, 30×, 20×, and 15× sequencing coverage. We ran CSA with these read sets using either the GRCh38 genome (best-case) or the *P. abelii* genome (diverged reference) as reference.

Best-case:

CSA2.6c.pl -r hs15x.fa.gz -g GRCh38.p12.CHR.fa.gz\

-t 80 -d HS25-GRCh38-2_6C\

-o HS25-GRCh38-2_6C > HS25-GRCh38-2_6C.bash

nohup bash\

HS25-GRCh38-2_6C.bash > HS25-GRCh38-2_6C.log 2>&1 &

 

CSA2.6c.pl -r hs20x.gz -g GRCh38.p12.CHR.fa.gz\

-t 80 -d HS33-GRCh38-2_6C\

-o HS33-GRCh38-2_6C > HS33-GRCh38-2_6C.bash

nohup bash\

HS33-GRCh38-2_6C.bash > HS33-GRCh38-2_6C.log 2>&1 &

 

CSA2.6c.pl -r hs30x.fa.gz -g GRCh38.p12.CHR.fa.gz\

-t 80 -d HS50-GRCh38-2_6C\

-o HS50-GRCh38-2_6C > HS50-GRCh38-2_6C.bash

nohup bash\

HS50-GRCh38-2_6C.bash > HS50-GRCh38-2_6C.log 2>&1 &

 

CSA2.6c.pl -r hs40x.fa.gz -g GRCh38.p12.CHR.fa.gz\

-t 80 -d HS67-GRCh38-2_6C\

-o HS67-GRCh38-2_6C > HS67-GRCh38-2_6C.bash

nohup bash\

HS67-GRCh38-2_6C.bash > HS67-GRCh38-2_6C.log 2>&1 &

 

Diverged reference:

CSA2.6c.pl -C\

HS25-GRCh38-2_6C.step1.fa -r hs 15x.fa.gz\

-g GCF_002880775.1_Susie_PABv2_genomic.fna.gz\

-t 80 -d HS25-PA-2_6C\

-o HS25-PA-2_6C > HS25-PA-2_6C.bash

nohup bash\

HS25-PA-2_6C.bash > HS25-PA-2_6C.log 2>&1 &

 

CSA2.6c.pl -C HS33-GRCh38-2_6C.step1.fa\

-r hs 20x.fa.gz\

-g GCF_002880775.1_Susie_PABv2_genomic.fna.gz\

-t 80 -d HS33-PA-2_6C\

-o HS33-PA-2_6C > HS33-PA-2_6C.bash

nohup bash\

HS33-PA-2_6C.bash > HS33-PA-2_6C.log 2>&1 &

 

CSA2.6c.pl -C\

HS50-GRCh38-2_6C.step1.fa -r hs 30x.fa.gz\

-g GCF_002880775.1_Susie_PABv2_genomic.fna.gz -t 80\

-d HS50-PA-2_6C\

-o HS50-PA-2_6C > HS50-PA-2_6C.bash

nohup bash\

HS50-PA-2_6C.bash > HS50-PA-2_6C.log 2>&1 &

CSA2.6c.pl -C\

HS67-GRCh38-2_6C.step1.fa -r hs 40x.fa.gz\

-g GCF_002880775.1_Susie_PABv2_genomic.fna.gz -t\

80 -d HS67-PA-2_6C\

-o HS67-PA-2_6C > HS67-PA-2_6C.bash

nohup bash\

HS67-PA-2_6C.bash > HS67-PA-2_6C.log 2>&1 &

### Benchmark Scenario 6—CSA using ultra-long reads

ULRs (N50 read length >50 kb) from ONT sequencing will be available to many researchers soon. CSA default parameters are currently tweaked for common SMRT or ONT long-read data (N50 < 30 kb). Nevertheless, 2 CSA parameters may be set to improve ULR assembly:

A) Set “-p 2” to circumvent issues with the wtdbg-cns tool (WTDBG, RRID:SCR_017225) that might otherwise crash on ULRs. CSA uses the “-p” option to set the WTDBG2 consensus caller (0 = wtdbg-cons (default); 1 = wtpoa-cons; consensus calculation in step 1; 2 = wtdbg-cons -S 0; wtpoa-cns is slower but a bit more accurate; wtdbg-cns with option -S 0 is more stable on very long reads).
B) Set “-l “-L 70000 –aln-min-length 25000 –keep-multiple-alignment-parts 1 –A” to vastly improve contig N50 on ULR datasets. Make sure you still have enough coverage (e.g., ∼30×) left when skipping reads with length <70,000 bp, otherwise try –L 60000 or –L 50000 and so on. CSA uses the –I “…” parameter to pass on detailed parameters to the wtdbg2 assembler. Parameters provided by –I “…” may overrule other wtdbg2 parameters set by CSA (e.g., -k, -s, -e, or -m).

We downloaded ULR data (CHM13 cell line) for benchmarking from the Telomere-to-Telomere (T2T) consortium [69].

We also downloaded the SHASTA genome assembly derived from these data for comparisons [70].

CSA was run with the following parameters:

CSA2.6c.pl -r rel2.fa.gz -g GRCh38.p12.CHR.fa.gz -t\

72 -d HS-ULR-2_6C -o HS-ULR-2_6C \

-p 2 -l "-L 70000 –aln-min-length 25000\

–keep-multiple-alignment-parts 1 -A" > HS-ULR-2_6C.bash

nohup bash HS-ULR-2_6C.bash > HS-ULR-2_6C.log 2>&1

### Dot plots and assembly comparisons

All CSA assemblies were compared to state-of-the-art reference genomes of the same species by MINIMAP2 using parameters for slightly diverged assembly-to-reference mapping (-x asm20, as we are dealing with unpolished consensus sequences here). PAF output files were filtered for MQ 60 (most unique) alignments and plotted by MINIDOT. PAF files were also analysed by custom scripts to combine splitted neighbouring alignments and count large-scale (>300 kb) fusions (or inter-chromosomal translocations), intra-chromosomal translocations, and inversions.

### Using CSA to close gaps in an existing scaffolded assembly by long reads

Users might want to use CSA only for gap closing in their existing scaffolded assemblies. They may split their scaffolds into contigs and then parameterize CSA with these contigs, while using the scaffolded contigs as reference (e.g., “… -C contigs_from_scaffolds.fa -g scaffolds.fa –r longreads.fa.gz…”).

It is also possible to use the last gap closure step (in CSA Step 4), which looks for neighbouring contig overlaps in scaffolds as a stand-alone procedure:

bash/your_path/CSA2.6/INSTALL/../script/STITCH.sh scaffolds.fa\

/your_path/CSA2.6/INSTALL/.. > scaffolds_with_joined_overlapping_contigs.fa

## Availability of Supporting Data and Materials

Code and benchmark snapshots are available in the Supplementary Material and in the *GigaScience* GigaDB repository [71]. Test data are publicly available at NCBI (BioProject details listed above).

## Availability of Source Code and Requirements

Project name: CSA—Chromosome-Scale Assembler

Project home page: https://github.com/HMPNK/CSA2.6 and Reference 68

Operating system(s): Linux

Programming language: PERL, AWK, and BASH scripting

Other requirements: CSA was tested on Ubuntu 18.04/19.04, Red Hat 8, OpenSuse Leap 15.1, CentOS 7

License: MIT

RRID:SCR_017960

biotoolsID: biotools: CSA2.6 (https://bio.tools/CSA2.6)

## Additional Files

**Additional File 1:** Supplementary Tables

**Additional File 2:** High-resolution Figures

**Additional File 3:** Code snapshot used for Benchmarks 1–5

**Additional File 4:** Code snapshot used for Benchmarks 6

**Supplementary Table S1:** CSA results of the best-case scenario, for representative genomes of mammals, birds, and fish

**Supplementary Table S2:** CSA results using divergent reference genomes

**Supplementary Table S3:** ONT read assembly supported by 10X Genomics, genus-level and order-level references

**Supplementary Table S4:** Influence of sequencing coverage on *H. sapiens* CSA assemblies

**Supplementary Table S5:** *H. sapiens* CSA assembly by ultra-long reads

**Supplementary Figure S1:** Additional dot plots for *H. sapiens* CSA assemblies using ONT ultra-long reads, or SMRT reads and more diverged reference genomes

**Supplementary Figure S2:** Additional dot plots for *T. guttata* CSA assemblies using SMRT reads and more diverged reference genomes

**Supplementary Figure S3:** Additional dot plot for *H. sapiens* CSA assembly using ONT ultra-long reads and a diverged reference genome (*P. abelii*)

giaa034_GIGA-D-19-00380_Original_Submission

giaa034_GIGA-D-19-00380_Revision_1

giaa034_GIGA-D-19-00380_Revision_2

giaa034_Response_to_Reviewer_Comments_Original_Submission

giaa034_Response_to_Reviewer_Comments_Revision_1

giaa034_Reviewer_1_Report_Original_Submission Matthias Zytnicki -- 12/9/2019 Reviewed

giaa034_Reviewer_1_Report_Revision_1 Matthias Zytnicki -- 2/18/2020 Reviewed

giaa034_Reviewer_1_Report_Revision_2 Matthias Zytnicki -- 3/11/2020 Reviewed

giaa034_Reviewer_2_Report_Original_Submission Daniel Zerbino -- 12/19/2019 Reviewed

giaa034_Reviewer_2_Report_Revision_1 Daniel Zerbino -- 2/1/2020 Reviewed

giaa034_Supplemental_Files

## Abbreviations

bp: base pairs; BUSCO: Benchmarking Universal Single-Copy Orthologs; CPU: central processing unit; CSA: Chromosome-Scale Assembler; ctg: contig; f: chromosomal fusion/fission; Gb: gigabase pairs; i: inversion; interchr.: inter-chromosomal; intrachr.: intra-chromosomal; Hs: *Homo sapiens*; IMAP: Integrative Meta-Assembly Pipeline; kb: kilobase pairs; Mb: megabase pairs; My: million years; Mya: million years ago; NCBI: National Center for Biotechnology Information; ONT: Oxford Nanopore Technologies; PacBio: Pacific Biosciences; RACA: reference-assisted chromosome assembly; RAM: random access memory; scf: scaffold; Sc: *Siniperca chuatsi*; SMRT: Single Molecule Real-Time; SRA: Sequence Read Archive; t: intra-chromosomal translocation; Tg: *Taeniopygia guttata*; ULR: ultra-long read; VGP: Vertebrate Genomes Project; WGD: whole-genome duplication; WGS: whole-genome shotgun.

## Competing Interests

The authors declare that they have no competing interests.

## Funding

This work was funded by the German Research Foundation (DFG) “Eigene Stelle” grant within the project “Reference genomes of the Chinese perch (*Siniperca chuatsi*), the Eurasian perch (*Perca fluviatilis*) and three related fish species of the family Sinipercidae for comparative genomics and marker assisted breeding in aquaculture” KU 3596/1-1; project number: 324050651.

## Authors' Contributions

H.K. designed, programmed, and benchmarked the CSA pipeline. C.K. performed independent tests of CSA. L.L., X.-F.L., and C.K. provided long-read data. H.K. wrote the manuscript with contributions from S.W., M.S., and C.K.
