# Supplementary material for: CSA: A high-throughput chromosome-scale assembly pipeline for vertebrate genomes
Source: Gigascience. 2020 May 25;9(5):giaa034. doi: 10.1093/gigascience/giaa034 (PMC7247394; doi:10.1093/gigascience/giaa034)
Supplement: giaa034_GIGA-D-19-00380_Original_Submission [file giaa034_giga-d-19-00380_original_submission.pdf]

GigaScience

CSA: A high-throughput chromosome-scale assembly pipeline for vertebrate genomes

--Manuscript Draft--

**Manuscript Number:**	GIGA-D-19-00380
**Full Title:**	CSA: A high-throughput chromosome-scale assembly pipeline for vertebrate genomes
**Article Type:**	Technical Note
**Funding Information:**	Deutsche Forschungsgemeinschaft (KU 3596/1-1; project number: 324050651) — Dr. Heiner Kuhl
**Abstract:**	Background:<br>Easy-to-use and fast bioinformatics pipelines for long-read assembly that go beyond the contig-level to generate high-quality chromosome-scale genomes from raw data remain scarce.<br>Results:<br>Chromosome Scale Assembler (CSA) is a novel computationally highly efficient bioinformatics pipeline that fills this gap. CSA integrates information from scaffolded assemblies (e.g. Hi-C or 10X Genomics) or even from diverged reference genomes into the assembly process. As CSA performs automated assembly of chromosome-sized scaffolds, we benchmark its performance against state-of-the art reference genomes that have been built in a laborious fashion using multiple separate assembly tools and manual curation. CSA increases the contig length using scaffolding, local re-assembly and gap-closing. On certain datasets, initial contig N50 may be increased up to 4.5-fold. For smaller vertebrate genomes, chromosome-scale assemblies can be achieved within 12 h using low cost, high-end desktop computers. Mammalian genomes can be processed within 16 h on compute-servers. Using diverged reference genomes for fish, birds and mammals, we demonstrate that CSA calculates chromosome-scale assemblies from long-read data and genome comparisons alone. Even contig-level draft assemblies of diverged genomes are helpful for reconstructing chromosome-scale sequences. CSA is capable of assembling ultra-long reads.<br>Conclusions:<br>CSA can speed-up and simplify chromosome-level assembly and significantly lower costs of large-scale family-level vertebrate genome projects.
**Corresponding Author:**	Heiner Kuhl<br>IGB Leibniz-Institute of Freshwater Ecology and Inland Fisheries<br>Berlin, Berlin GERMANY
**Corresponding Author Secondary Information:**	
**Corresponding Author's Institution:**	IGB Leibniz-Institute of Freshwater Ecology and Inland Fisheries
**Corresponding Author's Secondary Institution:**	
**First Author:**	Heiner Kuhl
**First Author Secondary Information:**	
**Order of Authors:**	Heiner Kuhl
	Ling Li
	Sven Wuertz
	Matthias Stoeck
	Xu-Fang Liang
	Christophe Klopp
**Order of Authors Secondary Information:**	
**Additional Information:**	

Powered by Editorial Manager® and ProduXion Manager® from Aries Systems Corporation

Question	Response
Are you submitting this manuscript to a special series or article collection?	No
**Experimental design and statistics**<br><br>Full details of the experimental design and statistical methods used should be given in the Methods section, as detailed in our Minimum Standards Reporting Checklist. Information essential to interpreting the data presented should be made available in the figure legends.<br><br>Have you included all the information requested in your manuscript?	Yes
**Resources**<br><br>A description of all resources used, including antibodies, cell lines, animals and software tools, with enough information to allow them to be uniquely identified, should be included in the Methods section. Authors are strongly encouraged to cite Research Resource Identifiers (RRIDs) for antibodies, model organisms and tools, where possible.<br><br>Have you included the information requested as detailed in our Minimum Standards Reporting Checklist?	Yes
**Availability of data and materials**<br><br>All datasets and code on which the conclusions of the paper rely must be either included in your submission or deposited in publicly available repositories (where available and ethically appropriate), referencing such data using a unique identifier in the references and in the "Availability of Data and Materials" section of your manuscript.	No

Have you have met the above requirement as detailed in our Minimum Standards Reporting Checklist?	
If not, please give reasons for any omissions below.<br><br>as follow-up to "**Availability of data and materials**<br><br>All datasets and code on which the conclusions of the paper rely must be either included in your submission or deposited in publicly available repositories (where available and ethically appropriate), referencing such data using a unique identifier in the references and in the "Availability of Data and Materials" section of your manuscript.<br><br>Have you have met the above requirement as detailed in our Minimum Standards Reporting Checklist?<br><br>"	Some datasets used for benchmarks are still under submission at NCBI (S.chuatsi and P.fluviatilis long read data and reference genome assemblies)

Manuscript
Click here to download Manuscript CSA-manuscript_v7c_GIG.docx

CSA: A high-throughput **c**hromosome-**s**cale **a**ssembly pipeline for vertebrate genomes

Heiner Kuhl[1]*, Ling Li [1,2], Sven Wuertz[1], Matthias Stoeck[1], Xu-Fang Liang[2] and Christophe Klopp[3]

*Corresponding author

Affiliations

[1] Department of Ecophysiology and Aquaculture, Leibniz-Institute of Freshwater Ecology and Inland Fisheries (IGB), Berlin, Germany.

[2] College of Fisheries, Chinese Perch Research Center, Huazhong Agricultural University; Innovation Base for Chinese Perch Breeding, Key Lab of Freshwater Animal Breeding, Ministry of Agriculture, Wuhan, China.

[3] Sigenae, Mathématiques et Informatique Appliquées de Toulouse, INRA, Castanet Tolosan, France.

E-mail addresses

Heiner Kuhl:	kuhl@igb-berlin.de
Ling Li:	ling.li@igb-berlin.de
Sven Würtz:	wuertz@igb-berlin.de
Matthias Stöck:	matthias.stoeck@igb-berlin.de
Xu-Fang Liang:	xfliang@mail.hzau.edu.cn
Christophe Klopp:	christophe.klopp@inra.fr

Abstract

Background: Easy-to-use and fast bioinformatics pipelines for long-read assembly that go beyond the contig-level to generate high-quality chromosome-scale genomes from raw data remain scarce.

Results: Chromosome Scale Assembler (CSA) is a novel computationally highly efficient bioinformatics pipeline that fills this gap. CSA integrates information from scaffolded assemblies (e.g. Hi-C or 10X Genomics) or even from diverged reference genomes into the assembly process. As CSA performs automated assembly of chromosome-sized scaffolds, we benchmark its performance against state-of-the art reference genomes that have been built in a laborious fashion using multiple separate assembly tools and manual curation. CSA increases the contig length using scaffolding, local re-assembly and gap-closing. On certain datasets, initial contig N50 may be increased up to 4.5-fold. For smaller vertebrate genomes, chromosome-scale assemblies can be achieved within 12 h using low cost, high-end desktop computers. Mammalian genomes can be processed within 16 h on compute-servers. Using diverged reference genomes for fish, birds and mammals, we demonstrate that CSA calculates chromosome-scale assemblies from long-read data and genome comparisons alone. Even contig-level draft assemblies of diverged genomes are helpful for reconstructing chromosome-scale sequences. CSA is capable of assembling ultra-long reads.

Conclusions: CSA can speed-up and simplify chromosome-level assembly and significantly lower costs of large-scale family-level vertebrate genome projects.

Keywords

Genome assembly, genome scaffolding, long-read, comparative genomics, genome evolution, chromosomes, vertebrates

Findings

Background

Whole genome shotgun (WGS) assembly in vertebrates – state of the art

WGS assembly of large vertebrate genomes has been an important topic of bioinformatic research over the last two decades, but obtaining completely assembled chromosomes through a single bioinformatics tool has not yet been achieved for large vertebrate genomes. Despite the ongoing replacement of short- by long-read sequencing in *de novo* genome projects, chromosome-level assemblies of vertebrates still require great bioinformatics expertise, especially in projects, where cutting-edge genome maps or scaffolding data are not available.

Today, most vertebrate genomes can be assembled using noisy long reads[1-3] and the results - in terms of assembly contiguity, measured as contig N50 - can outperform results obtained by short-read sequencing by a factor greater than 100. Contig N50 of today's noisy long-read assemblies reaches lengths similar to scaffold N50 of high-quality short-read genome assemblies some years ago. Still, current assembly tools can profit from their ancestors[4-9]. So far most of them produce only contigs[10-14] and do not incorporate additional information to order these contigs into scaffolds, which would enable further gap-closing and lead to chromosomal-level assemblies.

Chromosomal-level genome assembly as the final goal of a genome project still requires additional scaffolding or mapping data (Hi-C[15-17] or optical mapping[18], high density genetic linkage map[19]), resulting in additional efforts that may add significant human, time and financial resources to sequencing projects. For many, especially rare species, DNA-resources for *de novo* genome sequencing come from archival tissues (e.g. frozen or ethanol-fixed or preserved in other storage media), preventing the application of Hi-C, which requires living cells, and thus mapping panels can hardly be established. In such and similar cases, synteny and gene order analysis between evolutionary related genomes may be the only option to improve the genome assembly process.

Synteny as a common feature of vertebrate genomes

All vertebrates, with currently ca. 71,000 scientifically described species (August 2019), experienced two ancestral whole genome duplications (WGDs), leading to ca. 38,000 extant tetrapods, while most of the ca. 33,000 teleosts have gone through a 3rd WGD[20, 21]. Beyond different ancestral WGD-"substrates" that influenced the evolution of deletions, silencing and/or pseudogenization, sub- and neofunctionalization, genome size in vertebrates differs strongly (typical size range 0.4–4 Gbp; huge genome sizes of amphibians pose strong exceptions: c-value (haploid genome size) 3.3–57 pg (http://genomesize.com)). While neither associated with morphological complexity nor gene numbers, genome size differences are caused by quantities of various repetitive elements and other non-coding DNA, comprising up to 98% of vertebrate genomes[22].

Despite these WGD and size differences, "structural conservation" of vertebrate genomes as inferred from the distribution and positioning of genes on chromosomes, known as synteny, is a major feature of their evolution[23] with a pattern of conserved syntenic associations dating back 360 My[24], and even 600 My in other metazoans[25, 26]. Locations and order of genes (also referred to as "blocs") in genomes depend on phylogenetic relatedness and on the "substrates" evolved after the ancient WGDs. Despite synteny, the various classes of vertebrates show different speed of chromosomal and sequence, and thus genome evolution.

Teleost fishes exhibit accelerated evolutionary rate of protein-coding and other sequences, a higher rate of intron turnover, loss of many potential *cis*-regulatory elements and shorter conserved syntenic blocks[27, 28]. Due to their mostly enormous genome size with huge repetitive fractions, merely two handful of amphibian genomes have been sequenced and few reached chromosomal scale quality, with deep divergences (often >100 My) between systematic amphibian families posing additional challenges. Nevertheless, orthologs defining bird chromosomes are discretely ordered in the single well-assembled urodelan (*Ambystoma mexicanum*)[29, 30] and the few anuran genomes (*Xenopus tropicalis*, *Nanorana parkeri)*[31, 32], suggesting that ancestral chromosome segments and structures also remained conserved during amphibian phylogenesis[23]. Conservation of chromosomes, syntenic with avian autosomes, has been demonstrated in squamate reptiles[33], in

which numerous microchromosomes pose special challenges for genomics[34]. Whole-genome comparisons among birds and mammals point to regions in genomes where the order of orthologous sequences has been maintained for tens of millions of years[35, 36].

In summary, despite specific genomic properties of various vertebrate classes, synteny and conserved gene order present common and long-known inherent features of vertebrate genomes[37] that deserve to be better considered during genome assembly and that can be exploited by current bioinformatics.

Exploiting synteny information for new approaches in vertebrate genomics

Indeed, evolutionary relationships such as highly conserved chromosome structure (synteny and gene order) in related vertebrate species[38], in some taxa even between several taxonomic-levels[39], can enable low-cost approximations of chromosomal-scale assembly by comparative genomics[40, 41]. Of course, such an approach requires the existence of at least one suitable high-quality reference genome, which has become a dwindling problem as for each vertebrate order at least one "platinum grade" reference genome will soon be created in Phase I of the international Vertebrate Genome Project (VGP an offspring of the genome10K project)[42] and more will follow in the course of other large-scale genomics projects, like the Earth BioGenome Project (EBP)[43].

Here, we present a novel bioinformatics pipeline, which we call "Chromosomal Scale Assembler" (CSA). CSA overcomes limitations of current long-read assemblers by integrating comparisons between diverged reference genomes and/or scaffolds, derived from optical mapping, Hi-C or 10X Genomics into the *de novo* assembly process. CSA runs computationally highly efficient tools for long-read genome assembly, whole genome alignments and reference-assisted chromosomal assembly in an iterative fashion. We show that CSA is able to produce chromosomal-level assemblies for smaller vertebrate genomes (fishes, birds) within 12 h on low cost computing equipment (1000 – 2000 $, Intel i7, 128 GB RAM), using just long-read data and a diverged reference genome (div. time ~65 Mya) as input. Larger mammalian genomes, such as the human, can be assembled within 16 h on

server equipment (Intel Xeon, 1 TB RAM). Depending on the type and coverage of the input data, CSA is able to improve contig N50 length up to 4.5-fold from initial to final contig assembly.

Results and Discussion

Implementation of the CSA pipeline

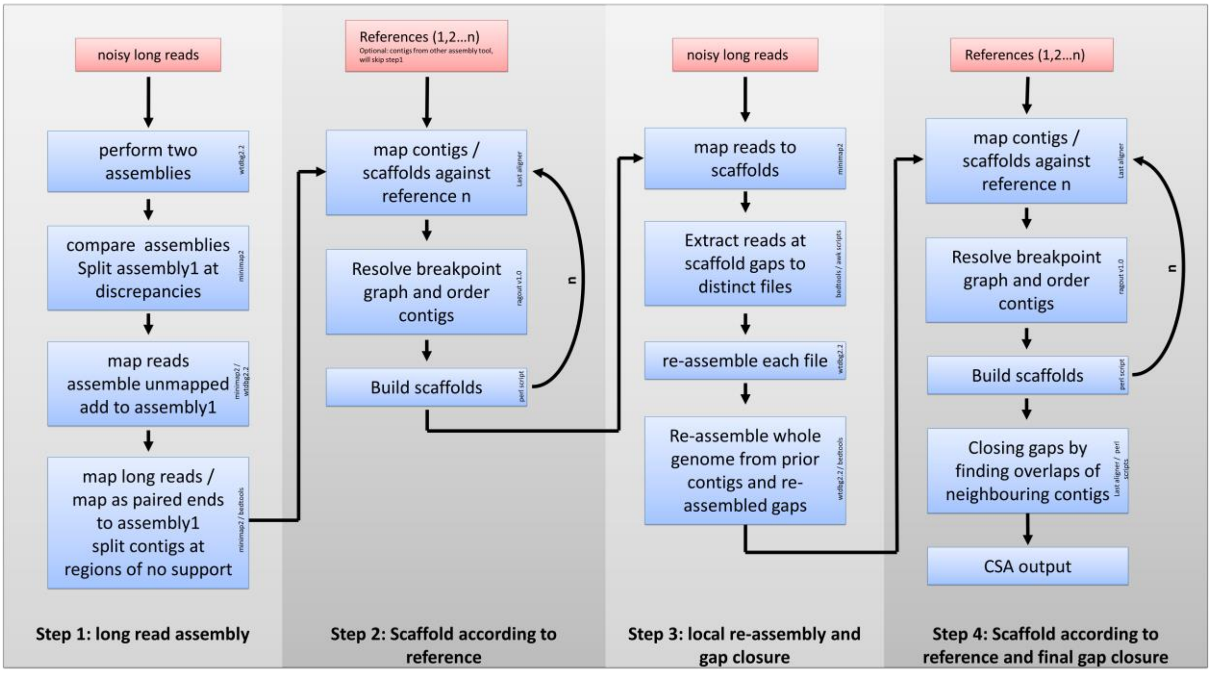

Figure 1: Flowchart of the four step CSA pipeline.

The first step of CSA (Figure 1) is a *de novo* assembly of noisy long-read data (either Pacific Bioscience or Oxford Nanopore data). It employs the WTDBG2 (version 2.2 11 Dec. 2018) assembler as it is among the most computationally efficient *de novo* genome assemblers to date[14]. CSA runs two WTDBG assemblies with slightly varying parameters and splits the contigs at discrepancies between the two assemblies to get rid of rarely occurring misassemblies. Additionally, we re-assemble long reads that can only be partially mapped to the WTDBG2 assembly (< 10 % of read-length); this step may recover up to 1-2 % of genomic sequence (large contigs) that is missing in current WTDBG2 primary assemblies. A final assembly curation is performed by re-mapping (MINIMAP2)[44] long reads and paired ends of long reads (500 bp from each end) to the assembly and split contigs at regions of zero coverage. If needed, the WTDBG2 assembly can be omitted and a contig file from

another genome assembly tool can be used by CSA; thus, CSA can also be used to update existing assemblies.

In the second step, the resulting curated contigs are mapped by LAST aligner[45] to one or more references. These references may be scaffolds from the same species that have been built using various methods (e.g. 10X Genomics, optical mapping or Hi-C). An outstanding feature of CSA is that even diverged reference and draft genomes are a suitable input. The LAST alignments are used by RAGOUT[46] to order the curated contigs (from step 1) into scaffolds, which then already may reach chromosomal size.

During step three all noisy long reads are mapped to the scaffolds by MINIMAP2. Reads that map in 20 kbp-windows around scaffold gaps or contig ends are extracted into distinct fasta files. These files are submitted to the WTDBG2 assembler and are locally re-assembled in parallel. The resulting local re-assemblies for each gap/contig end are then assembled with the primary WTDBG2 contigs that have been split into overlapping pseudo-reads to meet the read length limits of WTDBG2 (256 kb in version 2.2, version 2.4 has no limits, but showed lower performance in tests). As WTDBG2 now assembles pre-assembled reads with higher accuracy (consensus accuracy 98-99%), more stringent parameters are set. This iterative assembly step can typically double N50 contig sizes as shown in different tests hereafter. Alignment of the improved contigs to the prior scaffolds is used to remove few intra- and inter-scaffold misassemblies.

Step four, again, maps the improved contigs against the references used in step2, by LAST aligner and runs RAGOUT to order the contigs into scaffolds. Finally, some gaps with overlapping neighbouring contig-ends are identified by LAST and closed. In the following, we tested CSA on different scenarios and benchmark its performance. As CSA automatically performs many steps that traditionally required using different software tools and laborious manual curation, we do not compare its results against known contig-level genome assembly tools, but against the currently best chromosomal-scale reference genome assemblies for different vertebrate species.

Benchmark scenario 1: Updating existing fish, bird and mammal assemblies, using a prior assembly version as reference

The current CSA pipeline was tested using SMRT (single molecule real time; Pacific Biosciences) long-read sequencing data for representative species of three different vertebrate clades, namely mammalia (*Homo sapiens* = Hs), aves (*Taeniopygia guttata* = Tg) and teleostei (*Siniperca chuatsi* = Sc). Our first tests used high-quality genomes of the same species from which the long-read input data was derived to assist the assembly. These tests show what we can expect from CSA in a best-case scenario. In a real-world scenario, where no known reference of the same species is available, this approach would be comparable to using CSA and scaffolding the CSA step 1 assembly by Hi-C data and then continuing with assembly improvements (CSA step 2-4). The detailed results of these benchmarks are shown in suppl. table 1.

In terms of completeness of the chromosomal assembly, we measured, how much of the consensus sequence is contained in the top n largest scaffolds, where n is the haploid chromosome number. All CSA assemblies placed more than 94% of the consensus into the top n scaffolds (Hs = 97.5%; Tg = 94.2%; Sc = 99.3%). The contig N50 length was 25.9 Mbp, 27.7 Mbp and 16.5 Mbp for Hs, Tg and Sc, respectively. These values outperform the current reference contig N50 for Tg (VGP assembly) and Sc (own results), which are based on the same long-read input data but included different genome maps and curation steps to improve the assembly. For Hs we compared contig N50 to the so far best assembly from Pacbio (Acc: GCA_003634875.1) data and found that CSA produced similar values, although we used an older dataset (P4C6 chemistry from RSII sequencer) for our tests. For Tg, we could improve contig N50 by 2.3-fold over the VGP assembly. The contig N50 of Sc improved 1.35-fold over our sinChu7 assembly.

Finally, we compared CSA assemblies versus the references to visually inspect assembly errors by dot plots (Figure 2 top row) and counted larger scale synteny (gene order) breaks (rearranged genomic blocks >300 kbp) by custom scripts. The CSA assemblies exhibited only few structural misassemblies (**f** = interchr. fusion, **t** = intrachr. translocation, **i** = inversion: **Hs:** f: 0; t: 6; i: 2 / **Tg:** f: 0; t: 1; i: 4 / **Sc:**

f: 0; t: 1; i: 5). For the teleost assembly, CSA even polished two misassemblies in the current reference genome (1 fusion and one 1 inversion).

These results show that under our best case scenario the pipeline performed very well and CSA appears as a valuable tool to improve existing reference genomes by complete re-assembly as soon as improved sequence data is available.

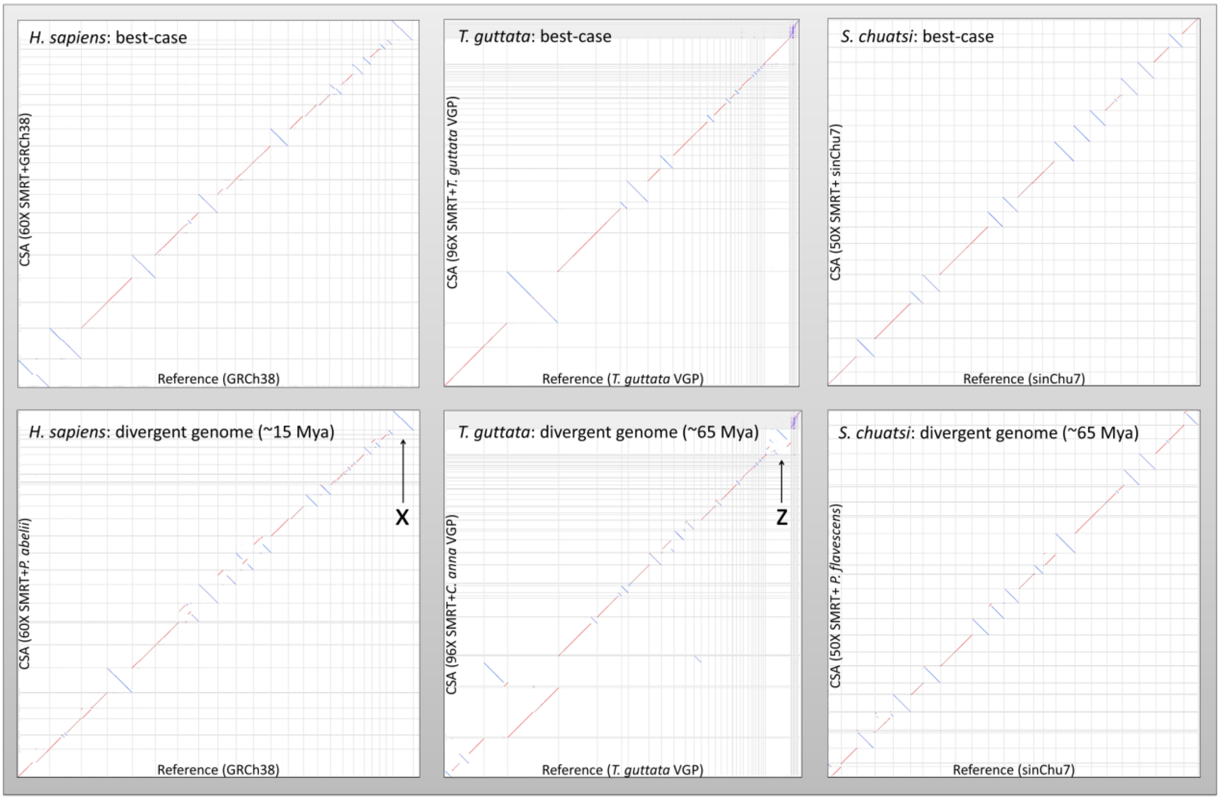

Figure 2: Dot plots of CSA results against reference genomes under best-case (top row) and diverged reference scenarios (bottom row) for mammal, bird and fish genomes.

Benchmark scenario 2: CSA using divergent genomes as reference, allows chromosomal scale assemblies from long reads only

Although this approach can be limited by complex evolutionary scenarios involving major re-arrangements of genomes, in principle, the mapping steps in CSA have been designed to allow for incorporation of highly diverged genomes as references. Nowadays (and in the future even more) one will find suitable, perhaps distantly related reference genomes for most vertebrate species in databases and this gives us the opportunity to obtain high-quality chromosome-scale assemblies from long-read data alone – potentially even without having other mapping data at hand (e.g. Hi-C, optical maps, linkage maps). We tested CSA on the long-read data from above using high-quality

reference genomes of species that diverged between 10-240 Mya as references. The detailed results of these benchmarks are shown in suppl. table 2.

Overall the fraction of consensus sequences assigned to the top n scaffolds, was slightly lower than under the best-case scenario, but well above 90% for the less diverged references. The loss of placed sequence typically occurs in the subtelomeric regions that diverge faster than the other chromosomal regions. In most cases, using more diverged reference genomes, CSA still allowed to place more than 92% of the assembly in the top n scaffolds.

Improvements of contig N50 were still observed at a similar scale as in the best-case scenario and introduced assembly errors due to divergent reference genomes were low on the contig-level (suppl. table 2; row: “Errors ctg”). Our main focus of this benchmark was to analyse large-scale misassemblies that are introduced by using diverged genomes as references in the chromosomal scaffold assembly (Figure 2 bottom row, additional plots suppl. figure 1 and 2) and how these develop with increasing divergence time. As expected, here we saw clear differences between mammals, birds and teleosts.

Chromosomal gene order is highly conserved in birds[39] and among vertebrates, bird genomes have the lowest fraction of repetitive sequences (< 20%)[47]. This possibly explains why CSA works very well for most of autosomes when using diverged bird genomes (up to 90 Mya) as reference. Nevertheless, here we found few chromosomal fusion errors that were related to known differences in bird karyotypes (e.g. fusion/fission of chr1/chr1A; chr4/chr4A etc.) and a clear enrichment of inversion and translocation errors on the Z-chromosome (*Gallus gallus*: 32% and *Calypte anna*: 35% of t and i errors on Z), possibly a result of fast evolution of the Z/W sex chromosomes, which has been decribed earlier[48]. Error profiles were f: 1; t: 33; i: 20 and f: 2; t: 26; i: 20 when using *C. anna* (~65 Mya) and *G. gallus* (~90 Mya) as reference respectively. Finally, CSA still worked reasonably well using the scaffold-level *Alligator mississipiensis* draft genome as reference which has diverged about 240 Mya (f: 2; t: 22; i: 18).

In mammals, assembly errors were distributed more evenly over autosomes and for the X-chromosome we did not find an enrichment of errors, like in the bird Z chromosome. The three-times

larger and more repetitive (> 30%) mammal genomes[47] were more prone to misassemblies with increasing divergence time of the reference than bird genomes. Still, CSA results were good for references that diverged 10-20 Mya ago (in our example *Pongo abelii:* f: 0; t: 58; i: 8).

In teleosts, we did not observe chromosome specific assembly issues and the increase of misassemblies with divergence of the reference was not as harsh as in mammals, possibly due to the more compact genomes of most teleosts. Thus, CSA performed well when using fish reference genomes with divergence times smaller than 65 Mya (here *Perca flavescens*: f: 1; t: 13; i: 16).

Our results show that long-read data and a well-chosen, order-level state-of-the-art reference genomes enable CSA to calculate high-quality assemblies for most chromosomes, but in some cases clade-specific problems have to be resolved by manual curation. As a rule of thumb, if choosing from reference genomes that have similar divergence times to support assembly of a new genome, those with the same haploid chromosome number and slowest evolution (as depicted by small branch length in phylogenetic trees or a high fraction of alignable sequence between assembled and reference genome) should be preferred. Nevertheless, considering the contig-level, the usage of distant genomes as references in CSA is quite safe and produces only few errors (supplementary table 2, compare “Errors scf” against “Errors ctg”), but still is able to highly improve contig N50.

Benchmark scenario 3: CSA on a fish genome, integrating Oxford Nanopore reads, 10X Genomics scaffolds and diverged reference genomes

The previous benchmarks did use SMRT long-read data and a single reference genome. In the following, we ran CSA using long reads generated by Oxford Nanopore (ONT) sequencing from genomic DNA of *Perca fluviatilis* and supported the assembly by a 10X Genomics assembly of the same species and two diverged reference genomes.

A high-quality chromosomal-scale genome assembly of *P. fluviatilis* assembled from the same ONT long reads and Hi-C sequencing has recently become available (Acc: under submission). The high-quality draft genome assembled from 10X Genomics sequence data has been published earlier[49]. As a close relative (genus-level) of *P. fluviatilis* a chromosomal-scale reference genome for *P.*

flavescens is available[50]; both *Perca* diverged about 8.2-17.5 Mya. As a more distantly related reference (div. time ~65 Mya), we used the chromosomal scale *Siniperca chuatsi* genome (Acc: PRJNA513951; under submission).

To test, if CSA could reach a similar assembly quality as for the *P. fluviatilis* reference genome, we ran CSA on *P. fluviatilis* ONT long reads supported by 10X Genomics scaffolds, *P. flavescens* and *S. chuatsi* genomes (see suppl. table 3).

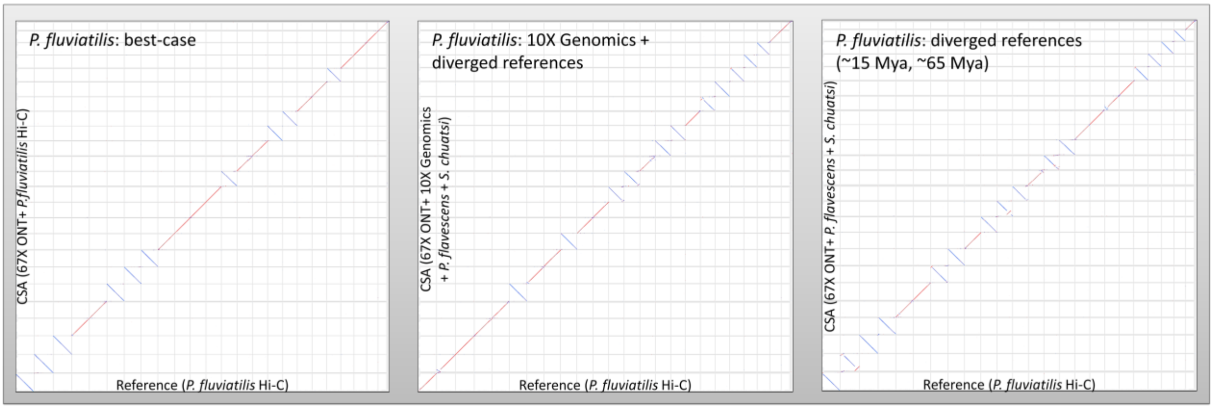

Figure 3: Dot plots of CSA results against reference genome for assembly of *Perca fluviatilis* Oxford Nanopore data sequentially supported by 10X Genomics and two diverged references genomes.

CSA managed to place 94.3% of the assembled sequence into 24 large scaffolds, which corresponded to the reference chromosomes. Only few differences (f: 0; t: 12; i: 13) in chromosomal structure were apparent in the dot plot (Figure 3). CSA increased the contig N50 nearly 3.1-fold compared to the reference genome. The total time to complete the chromosomal assembly was 5h:30min, when using 80 CPU threads on a HPC server or 12 h on a high end desktop computer (12 CPU threads, 128 GB RAM). We polished the CSA assembly using MEDAKA (by ONT: https://github.com/nanoporetech/medaka) and PILON[51] and performed BUSCO[52], which confirmed that the assembly was highly complete on the gene level (Actinopterygii dataset, C: 95.9%, F: 2.1%, M: 2.0%, n: 4584).

Next, we performed the same runs and omitted the 10X Genomics scaffold data. This only resulted in a slight loss of sequences placed in chromosomes (now 93.8%; loss 0.5%) and a few more intrachr. translocations and inversions (f: 0; t: 19; i: 15).

Finally, using only the most diverged reference still resulted in 86.3% of sequence placed in the top 24 scaffolds, but more intra-chromosomal translocations (f: 0; t: 57; i: 16). Yet, improvement of contig N50 was still 2.7-fold and structural errors in contigs were low (f: 0; t: 0; i: 4).

Thus, CSA was able to compute chromosome-scale assemblies by sequentially using species-, genus- and order-level references together with ONT long-read data. The species-level reference (10X Genomics, Supernova assembly) was only slightly contributing to the final assembly due to its lower N50 scaffold length of 6.3 Mbp. We have observed that ONT long-read datasets of comparable N50 read length and coverage produce less contiguous assemblies than SMRT datasets, possibly due to coverage bias of genomic sequences that interfere with ONT sequencing. According to our results the two gap closure steps performed by CSA were highly efficient to improve contig N50 in such a situation.

Benchmark scenario 4: CSA using draft assemblies as reference, contig-level assemblies of diverged species may be highly complementary

Under scenario 2 we already found that draft assemblies of other species could be used to improve genome assemblies (*T. guttata* / *A. mississipiensis* results). So we asked the question, if a diverged, low N50 contig-level assembly could still support CSA to result in improved assemblies.

Thus, we assembled the *S. chuatsi* genome using *P. fluviatilis* contigs (from scenario 3 CSA step 1: N50 = 2.8Mbp) as reference. Although the reference contig N50 was relatively low, it was improving the *S. chuatsi* assembly significantly (suppl. table 2 last column). The *S. chuatsi* assembly continuity doubled from a N50 11.6 Mbp (primary contigs) to 23.4 Mbp (final scaffolds). The top 24 scaffolds consisted of 77.26% and the top 48 scaffolds consisted of 89.9% of the assembled sequence, thus chromosomal assembly was less complete, but possibly most chromosome arms were well assembled (Figure 4). Interestingly, the improvement of contig N50 (1.37-fold) due to gap closure was similar to the tests performed with high-quality reference genomes in scenario2 and the number of assembly errors was low (scaffolds: f: 0; t: 2; i: 9 / contigs: f: 0; t: 0; i: 5).

Thus, CSA is able to use even low continuous contig assemblies of diverged species to improve genome assemblies. This opens up new strategies in projects, where many species of a certain clade are sequenced and might complement the assemblies of each other already at draft state.

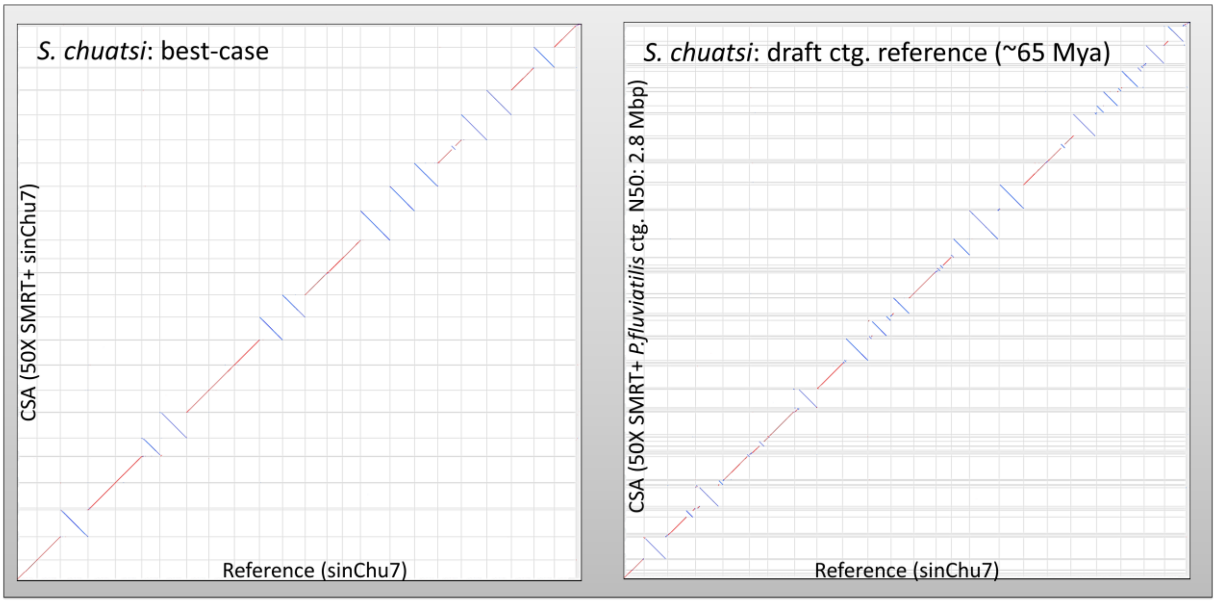

Figure 4: Dot plots of CSA results against reference genome for assembly of *Siniperca chuatsi* supported by contigs of a diverged draft genome assembly in comparison to the best case CSA assembly.

Benchmark scenario 5: Benchmarking influence of long-read sequencing coverage

On primary assemblies of lower contig N50 length, CSA can play its strength in gap closure. As this was already observed in scenario 3, we now asked the question how long-read sequencing coverage does influence the results of CSA assembly. We randomly subsampled reads from the *H. sapiens* 60x SMRT sequencing dataset, to obtain subsets of 15x, 20x, 30x and 40x sequencing coverage. We observed only slight changes of the final results for 60x, 40x and 30x sequencing coverage. Although contig N50 of the primary assembly started to drop below 30x, the CSA gap closures in step 3 and 4 still enabled a final contig N50, similar to what was obtained from the 40x and 60x datasets (suppl. table 4). The 20x and 15x data had significantly lower contig N50, here the improvement by the CSA gap closure was clearly the highest (3.8-fold for 20x and 4.5-fold for 15x), but assembly errors (especially fusion errors) started to increase (Figure 5). Similar results were observed, if using the diverged *P. abelii* genome as reference.

Thus, when running CSA, 30x sequencing coverage is sufficient and even lower coverage may lead to respectable results. Particularly, low coverage assemblies take profit from gap closure steps, and CSA can improve contig N50s by several hundred percent.

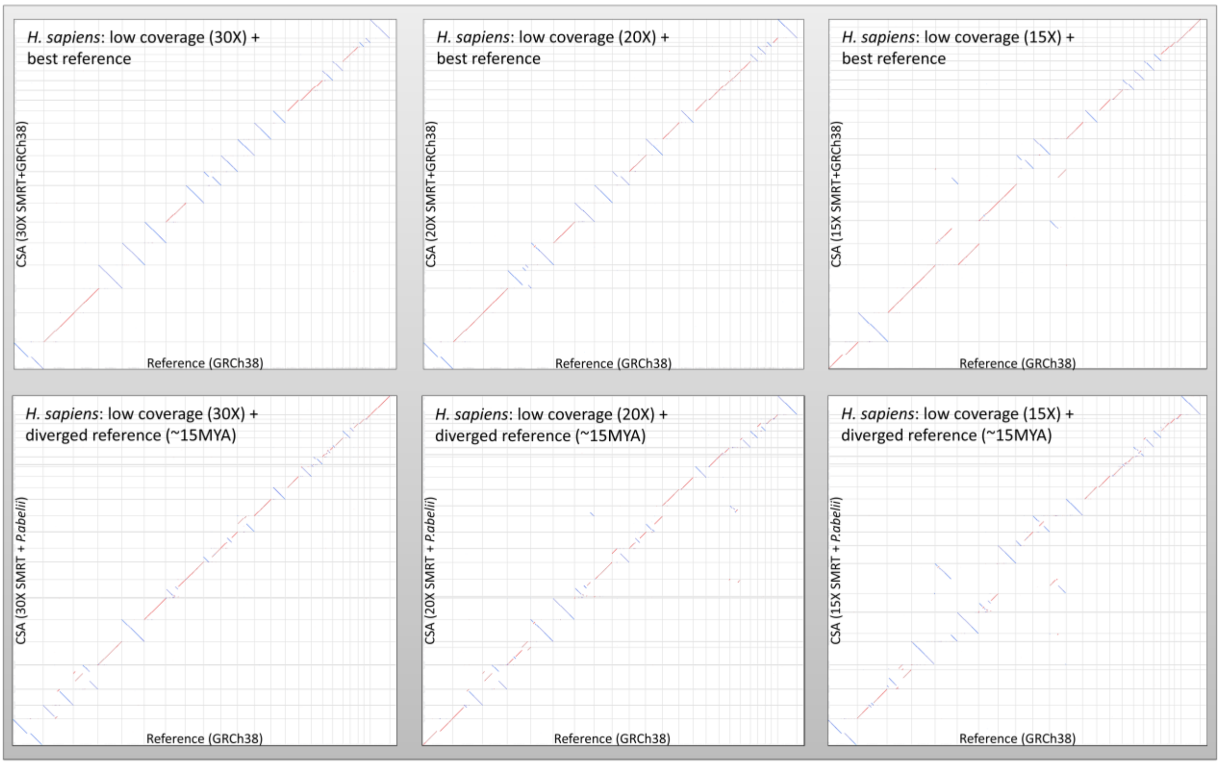

Figure 5: Dot plots of CSA results against reference genome for reduced coverage data (*Homo sapiens*), using either the best reference or a diverged reference to support CSA.

Benchmark scenario 6: Ultra long-read assembly

Ultra-long reads (ULR, N50 read length > 50 kbp) are currently gaining importance in the sequencing community and will possibly be available to many researchers soon. CSA default parameters have been optimized for current long-read data (N50 read length < 30 kbp). It has been reported recently that WTDBG2 performs relatively poor on ULR data compared to the SHASTA assembler, which was designed for ULR assembly[53]. We found that optimization of some parameters of WTDBG2 did overcome these issues (increasing minimum read length cut-off to about N50 read length, while maintaining sequence coverage >25X and increasing minimum overlap cut-off to about 30% of the N50 read length). We found that CSA was running more slowly due to its several read re-mapping steps, which are computationally less efficient when using ULR data. Still, the assembly finished within 24 h on our compute-server. Our CSA ULR assembly (best-case, using GRCh38 as reference;

details: suppl. table 5) did compete well in terms of contig N50 (48.4 Mbp vs. 46.0 Mbp) with the SHASTA assembler, producing significantly less contigs (1,526 vs. 1,925) and more complete total consensus length (2.9 Mbp vs. 2.8 Mbp, before sequence polishing). The number of structural misassemblies in CSA scaffolds, if compared against GRCh38 human reference chromosomes, was similar to using SMRT reads in scenario 1 (f:0; t:9; i:2, dot plot in suppl. figure 1). On the contig-level, we could compare CSA and SHASTA assemblies, which were both nearly free of large structural errors (CSA: f:0; t:0; i:2 / SHASTA: f:0; t:0; i:0).

Conclusions

Considering the scenarios tested, we have shown that CSA is a reliable tool that goes far beyond the contig-level assembly of long reads and enables automated chromosome-scale assemblies. Nevertheless, well-known assembly issues, like genomes exhibiting high heterozygosity, higher ploidies or extreme repeat content and genome size may still result in assemblies of lower contiguity. For example, the few available high-quality amphibian genomes, are currently posing challenges to CSA for this vertebrate class, as long as no high-quality Hi-C scaffolds from at least a relatively closely related species are available to support the assembly (model genomes from each systematic amphibian family would pose a great progress).

Yet, considering mammals, birds, fishes and possibly reptiles, CSA allows for lower sequencing coverage in genome projects and reduces the need for computational resources. Thus, CSA can contribute to save significant human, time and financial resources and thus cost-reduction in small- and large-scale genome projects. Furthermore, CSA enables beginners to genome assembly to perform chromosomal-level assemblies, even on datasets that would be considered suboptimal, when using other assembly tools. We are confident that CSA presents another important step towards the democratization of genome sequencing and assembly.

Acknowledgements

We thank Yann Guiguen for granting early access to *P. fluviatilis* long-read data. We also thank the authors of various tools that make up the CSA pipeline, especially: Jue Ruan (WTDBG2), Heng Li (MINIMAP2), Mikhail Kolmogorov (RAGOUT) and Martin C. Frith (LAST). We thank the Vertebrate Genomes Project (VGP) and the Telomere-to-Telomere (T2T) consortium for making reference genome assemblies and read-data publicly available for benchmarking purposes.

Availability of supporting data

Code snapshots are available in the manuscript supplements. Test data is publicly available at NCBI or is currently under submission to NCBI.

Availability and requirements

Project name: CSA – Chromosome-scale Assembler

Project home page: https://github.com/HMPNK/CSA2.6

Operating system(s): Linux

Programming language: PERL and BASH scripting

Other requirements: CSA was tested on Ubuntu 18.04/19.04 , Red Hat 8, OpenSuse Leap 15.1, CentOS 7

Any restrictions to use by non-academics: none

Additional files

Additional file 1: Supplementary tables.

Additional file 2: High-resolution figures.

Additional file 3: Code snapshot used for benchmarks 1-5.

Additional file 4: Code snapshot used for benchmarks 6.

Abbreviations

CPU: Central Processing Unit; CSA: Chromosome-Scale Assembler; ctg: contig; Mya: Million years ago; ONT: Oxford Nanopore Technologies; PacBio: Pacific Biosciences; scf: scaffold; SMRT: Single Molecule Real-Time; WGS: Whole Genome Shotgun;

Competing interests

The authors declare that they have no competing interests.

Funding information

This work was funded by the German Research Foundation (DFG) "Eigene Stelle" grant within the project "Reference genomes of the Chinese perch (*Siniperca chuatsi*), the Eurasian perch (*Perca fluviatilis*) and three related fish species of the family Sinipercidae for comparative genomics and marker assisted breeding in aquaculture" KU 3596/1-1; project number: 324050651.

Authors contributions

HK designed, programmed and benchmarked the CSA pipeline. **CK** performed independent tests of CSA. **LL**, **XF** and **CK** provided long-read data. **HK** wrote the manuscript with contributions from **SK**, **MS** and **CK**.

Methods

CSA Github project

CSA2.6 and future updates can be downloaded from "https://github.com/HMPNK/CSA2.6". All tools needed to run the pipeline will be installed by a script in the folder "CSA2.6/INSTALL". Simply run "bash INSTALL.bash" and follow the instructions. Some system specific installation issues are mentioned on GitHub. We have tested CSA2.6 on fresh server installations of Red Hat 8 and Ubuntu 18.04/19.04, OpenSuse LEAP 15.1 and CentOS 7 as well as older Red Hat and Ubuntu versions.

Due to ongoing development of the CSA pipeline we provide the code that has been used to benchmark scenarios 1 - 5 and 6 (see supplementary files: "CSA2.6c_benchmarks1-5.tar.gz" and "CSA2.6c_tweaked_ for_ULRs.tar.gz") with this manuscript.

CSA default parameters are currently tweaked for Pacbio RSII and ONT reads (30-60X, N50 readlength 10-30 kbp) We have found that some SEQUEL datasets behave quite different, here adding custom parameters for WTDBG2 will help: -l "-p 0 -k 15 -L5000 -S 2 -A" .

Benchmark scenario 1 – Data and CSA parameters

For the best-case scenario we downloaded reference genomes for *H. sapiens* (GRCh38.p12; RefSeq assembly accession: GCF_000001405.38, here we kept only the chromosomes and removed alternative loci) and *T. guttata* (bTaeGut1_v1.p; RefSeq assembly accession: GCF_003957565.1). For *S. chuatsi* we used our new reference genome sinChu7 (BioProject accession: PRJNA513951, under submission). SMRT long-read data for *H. sapiens* was downloaded from the SRA accession: SRP044331. SMRT long-read data for *T. guttata* was downloaded from SRA using the accessions: SRR5224495 - SRR5224503. The SMRT data for *S. chuatsi* will also be available through the BioProject accession: PRJNA513951. All SMRT data were selected for longest subreads and converted to gzip compressed fasta files.

CSA assemblies were run by the following commands:

```
CSA2.6c.pl -r homSap_longest_subreads.fa.gz  -g GRCh38.p12.CHR.fa.gz -t 80 -d HS-GRCh38-2_6C -o HS-GRCh38-2_6C > HS-GRCh38-2_6C.bash
nohup bash HS-GRCh38-2_6C.bash > HS-GRCh38-2_6C.log 2>&1 &

CSA2.6c.pl -r taeGut_SMRT.fa.gz -g bTaeGut1_v1.p.fasta.gz -t 80 -o TG-TG-VGP-2_6C -d TG-TG-VGP-2_6C > TG-TG-VGP-2_6C.bash
nohup bash TG-TG-VGP-2_6C.bash > TG-TG-VGP-2_6C.log 2>&1 &

CSA2.6c.pl -r PACBIO-READS-RAW.fa.gz -g sinChu7.fasta -o SC-SC-2_6C -d SC-SC-2_6C -t 80 > SC-SC-2_6C.bash
bash SC-SC-2_6C.bash > SC-SC-2_6C.log 2>&1 &
```

Benchmark scenario 2 – Data and CSA parameters

For our diverged reference scenario we downloaded the following genome assemblies.

Mammals: *P. abelii* (Acc: GCF_002880775.1); *C. jacchus* (Acc: GCA_002754865.1); *L. canadensis* (Acc: GCF_007474595.1); *O. anatinus* (Acc: GCF_004115215.1).

Birds: *C. anna* (Acc: GCF_003957555.1); *G. gallus* (Acc: GCF_000002315.6).

Reptile: *A. mississipiensis* (Acc: GCF_000281125.3).

Fish: *P. flavescens* (Acc: GCF_004354835.1).

CSA assemblies were run as above, but omitting the primary assembly step. As the primary assemblies were already calculated under scenario 1 (CSA step1 is a pure *de novo* assembly without support by reference), we can just add the fasta contigs using the parameter –C to save computing time (this procedure would also allow using primary assemblies from other assembly tools than WTDBG2):

H. sapiens

```
CSA2.6c.pl -C HS-GRCh38-2_6C.step1.fa -r homSap_longest_subreads.fa.gz  -g GCF_002880775.1_Susie_PABv2_genomic.fna.gz -t 80 -d HS-PA-2_6C -o HS-PA-2_6C > HS-PA-2_6C.bash
nohup bash HS-PA-2_6C.bash > HS-PA-2_6C.log 2>&1 &

CSA2.6c.pl -C HS-GRCh38-2_6C.step1.fa -r homSap_longest_subreads.fa.gz  -g GCA_002754865.1_ASM275486v1_genomic.fna.gz -t 80 -d HS-CJ-2_6C -o HS-CJ-2_6C > HS-CJ-2_6C.bash
nohup bash HS-CJ-2_6C.bash > HS-CJ-2_6C.log 2>&1 &

CSA2.6c.pl -C HS-GRCh38-2_6C.step1.fa -r homSap_longest_subreads.fa.gz  -g mLynCan4_s2.fasta.gz -t 80 -d HS-LC-2_6C -o HS-LC-2_6C > HS-LC-2_6C.bash
nohup bash HS-LC-2_6C.bash > HS-LC-2_6C.log 2>&1 &

CSA2.6c.pl -C HS-GRCh38-2_6C.step1.fa -r homSap_longest_subreads.fa.gz  -g GCF_004115215.1_mOrnAna1.p.v1_genomic.fna.gz -t 80 -d HS-OA-2_6C -o HS-OA-2_6C > HS-OA-2_6C.bash
nohup bash HS-OA-2_6C.bash > HS-OA-2_6C.log 2>&1 &
```

T. guttata

```
CSA2.6c.pl -C TG-TG-VGP-2_6C.step1.fa -r taeGut_SMRT.fa.gz -g bCalAnn1_v1.p.fasta.gz -t 80 -o TG-CA-VGP-2_6C -d TG-CA-VGP-2_6C > TG-CA-VGP-2_6C.bash
nohup bash TG-CA-VGP-2_6C.bash > TG-CA-VGP-2_6C.log 2>&1 &

CSA2.6c.pl -C TG-TG-VGP-2_6C.step1.fa -r taeGut_SMRT.fa.gz -g GCF_000002315.6_GRCg6a_genomic.fna.gz -t 80 -o TG-GG-2_6C -d TG-GG-2_6C > TG-GG-2_6C.bash
nohup bash TG-GG-2_6C.bash > TG-GG-2_6C.log 2>&1 &

CSA2.6c.pl -C TG-TG-VGP-2_6C.step1.fa -r taeGut_SMRT.fa.gz -g GCF_000281125.3_ASM28112v4_genomic.fna.gz -t 80 -o TG-AM-2_6C -d TG-AM-2_6C > TG-AM-2_6C.bash
nohup bash TG-AM-2_6C.bash > TG-AM-2_6C.log 2>&1 &
```

S. chuatsi

```
CSA2.6c.pl -C SC-SC-2_6C.step1.fa -r PACBIO-READS-RAW.fa.gz  -g GCF_004354835.1_PFLA_1.0_genomic.fna.gz -o SC-PFLA-2_6C -d SC-PFLA-2_6C -t 80 > SC-PFLA-2_6C.bash
nohup bash SC-PFLA-2_6C.bash > SC-PFLA-2_6C.log 2>&1 &
```

Benchmark scenario 3 – Data and CSA parameters

To assemble the *Perca fluviatilis* genome by CSA we used the *P. fluviatilis* reference genome (Acc: under submission) for the best case scenario. A 10X Genomics Supernova assembly (Acc: GCA_003412525.1) of *P. fluviatilis* as well as the *P. flavescens* and the *S. chuatsi* genomes from above were used to benchmark CSA using multiple references sequentially. Oxford Nanopore long-read data for *P. fluviatilis* was obtained from (Acc: under submission). CSA parameters for the best case scenario were:

```
CSA2.6c.pl -r perFlu_ONT_ALL.fa.gz -g Perca_fluviatilis.PFLU1.1.dna.toplevel.fa.gz -t 80 -o PF-PF-HiC-2_6C -d PF-PF-HiC-2_6C > PF-PF-HiC-2_6C.bash
nohup bash PF-PF-HiC-2_6C.bash > PF-PF-HiC-2_6C.log 2>&1 &
```

Again for the assembly using multiple references, we used the primary contigs from above (-C), now adding the reference sequences for sequential improvement as a comma separated list (e.g. -g closest.fa,less_diverged.fa,most_diverged.fa):

```
CSA2.6c.pl -C PF-PF-HiC-2_6C.step1.fa -r perFlu_ONT_ALL.fa.gz -g GCA_003412525.1_UTU_Pfluv_1.1_genomic.fna.gz,Perca_flavescens.PFLA1.1.dna.toplevel.fa.gz,sinChu7.fasta -t 80 -o PF-10X-PFLA-SC-
2_6C -d PF-10X-PFLA-SC-2_6C > PF-10X-PFLA-SC-2_6C.bash
nohup bash PF-10X-PFLA-SC-2_6C.bash > PF-10X-PFLA-SC-2_6C.log 2>&1 &

CSA2.6c.pl -C PF-PF-HiC-2_6C.step1.fa -r perFlu_ONT_ALL.fa.gz -g Perca_flavescens.PFLA1.1.dna.toplevel.fa.gz,sinChu7.fasta -t 80 -o PF-PFLA-SC-2_6C -d PF-PFLA-SC-2_6C > PF-PFLA-SC-2_6C.bash
nohup bash PF-PFLA-SC-2_6C.bash > PF-PFLA-SC-2_6C.log 2>&1 &

CSA2.6c.pl -C PF-PF-HiC-2_6C/01_WTDBG/PF-PF-HiC-2_6C.step1.fa -r ../DATA/perFlu/perFlu_ONT_ALL.fa.gz -g ../REFERENCES/sinChu7.fasta -t 80 -o PF-SC-2_6C -d PF-SC-2_6C > PF-SC-2_6C.bash
nohup bash PF-SC-2_6C.bash > PF-SC-2_6C.log 2>&1
```

Benchmark scenario 4 – Data and CSA parameters

Here we used the relatively low N50 contig length primary assembly of *P. fluviatilis* from scenario 3 to assemble the *S. chuatsi* SMRT data from above:

```
CSA2.6c.pl -C SC-SC-2_6C.step1.fa -r PACBIO-READS-RAW.fa.gz  -g PF-PF-HiC-2_6C.step1.fa -o SC-PFdraft-2_6C -d SC-PFdraft-2_6C -t 80 > SC-PFdraft-2_6C.bash
nohup bash SC-PFdraft-2_6C.bash > SC-PFdraft-2_6C.log 2>&1 &
```

Benchmark scenario 5 – Data and CSA parameters

To get subsets of the *H. sapiens* SMRT data we used SEQTK to randomly subsample reads from the full dataset in a way that we obtained about 40x, 30x, 20x and 15x sequencing coverage. We ran CSA with these read sets using either the GRCh38 genome (best-case) or the *P. abelii* genome (diverged reference) as reference.

Best-case:

```
CSA2.6c.pl -r hs15x.fa.gz  -g GRCh38.p12.CHR.fa.gz -t 80 -d HS25-GRCh38-2_6C -o HS25-GRCh38-2_6C > HS25-GRCh38-2_6C.bash
nohup bash HS25-GRCh38-2_6C.bash > HS25-GRCh38-2_6C.log 2>&1 &

CSA2.6c.pl -r hs20x.gz  -g GRCh38.p12.CHR.fa.gz -t 80 -d HS33-GRCh38-2_6C -o HS33-GRCh38-2_6C > HS33-GRCh38-2_6C.bash
nohup bash HS33-GRCh38-2_6C.bash > HS33-GRCh38-2_6C.log 2>&1 &

CSA2.6c.pl -r hs30x.fa.gz  -g GRCh38.p12.CHR.fa.gz -t 80 -d HS50-GRCh38-2_6C -o HS50-GRCh38-2_6C > HS50-GRCh38-2_6C.bash
nohup bash HS50-GRCh38-2_6C.bash > HS50-GRCh38-2_6C.log 2>&1 &

CSA2.6c.pl -r hs40x.fa.gz  -g GRCh38.p12.CHR.fa.gz -t 80 -d HS67-GRCh38-2_6C -o HS67-GRCh38-2_6C > HS67-GRCh38-2_6C.bash
nohup bash HS67-GRCh38-2_6C.bash > HS67-GRCh38-2_6C.log 2>&1 &
```

Diverged reference:

```
CSA2.6c.pl -C HS25-GRCh38-2_6C.step1.fa -r hs 15x.fa.gz -g GCF_002880775.1_Susie_PABv2_genomic.fna.gz -t 80 -d HS25-PA-2_6C -o HS25-PA-2_6C > HS25-PA-2_6C.bash
nohup bash HS25-PA-2_6C.bash > HS25-PA-2_6C.log 2>&1 &

CSA2.6c.pl -C HS33-GRCh38-2_6C.step1.fa -r hs 20x.fa.gz -g GCF_002880775.1_Susie_PABv2_genomic.fna.gz -t 80 -d HS33-PA-2_6C -o HS33-PA-2_6C > HS33-PA-2_6C.bash
nohup bash HS33-PA-2_6C.bash > HS33-PA-2_6C.log 2>&1 &

CSA2.6c.pl -C HS50-GRCh38-2_6C.step1.fa -r hs 30x.fa.gz -g GCF_002880775.1_Susie_PABv2_genomic.fna.gz -t 80 -d HS50-PA-2_6C -o HS50-PA-2_6C > HS50-PA-2_6C.bash
nohup bash HS50-PA-2_6C.bash > HS50-PA-2_6C.log 2>&1 &

CSA2.6c.pl -C HS67-GRCh38-2_6C.step1.fa -r hs 40x.fa.gz -g GCF_002880775.1_Susie_PABv2_genomic.fna.gz -t 80 -d HS67-PA-2_6C -o HS67-PA-2_6C > HS67-PA-2_6C.bash
nohup bash HS67-PA-2_6C.bash > HS67-PA-2_6C.log 2>&1 &
```

Benchmark scenario 6 – CSA using ultra long reads

Ultra long reads (ULR, N50 readlength > 50 kbp) from Oxford Nanopore sequencing will be available to many researchers soon. CSA default parameters are currently tweaked for common SMRT or ONT long-read data (N50<30 kbp). Nevertheless, two CSA parameters may be set to improve ultra long-read assembly:

A) Set '-p 2' to circumvent issues with the wtdbg-cns tool that might otherwise crash on ultra long reads.

B) Set '-l "-L 70000 --aln-min-length 25000 --keep-multiple-alignment-parts 1 –A' to vastly improve contig N50 on ultra long-read datasets. Make sure you still have enough coverage (e.g. ~30x) left when skipping reads with length below 70000bp, otherwise try –L 60000 or –L 50000 and so on.

We downloaded ULR data (CHM13 cell line) for benchmarking from the Telomere-to-Telomere (T2T) consortium:

https://s3.amazonaws.com/nanopore-human-wgs/chm13/nanopore/rel2/rel2.fastq.gz

We also downloaded the SHASTA genome assembly derived from this data for comparisons:

https://s3-us-west-2.amazonaws.com/human-pangenomics/assemblies/raw/shasta/CHM13.shasta.fasta

CSA was run with the following parameters:

```
CSA2.6c.pl -r rel2.fa.gz  -g GRCh38.p12.CHR.fa.gz -t 72 -d HS-ULR-2_6C -o HS-ULR-2_6C -p 2 -l "-L 70000 --aln-min-length 25000 --keep-multiple-alignment-parts 1 -A" > HS-ULR-2_6C.bash
nohup bash HS-ULR-2_6C.bash > HS-ULR-2_6C.log 2>&1
```

Dot plots and assembly comparisons

All CSA assemblies were compared to state-of-the-art reference genomes of the same species by MINIMAP2 using parameters for slightly diverged assembly-to-reference mapping (-x asm20, as we are dealing with unpolished consensus sequences here). PAF output files were filtered for MQ 60 (most unique) alignments and plotted by MINIDOT. PAF files were also analysed by custom scripts to combine splitted neighbouring alignments and count large scale (>300 kb) fusions (or inter-chromosomal translocations), intra-chromosomal transloctions and inversions.

Using CSA to close gaps in an existing scaffolded assembly by long reads

Users might want to use CSA only for gap closing their existing scaffolded assemblies. They may split their scaffolds in contigs and then parametrize CSA with these contigs, while using the scaffolded contigs as reference (e.g. "…. -C contigs_from_scaffolds.fa -g scaffolds.fa –r longreads.fa.gz….").

It is also possible to use the last gap closure step (in CSA-step4), which looks for neighbouring contig overlaps in scaffolds as a stand-alone procedure:

```
bash /your_path/CSA2.6/INSTALL/../script/STITCH.sh scaffolds.fa /your_path/CSA2.6/INSTALL/.. > scaffolds_with_joined_overlapping_contigs.fa
```

References

1. Gordon D, Huddleston J, Chaisson MJ, Hill CM, Kronenberg ZN, Munson KM, et al. Long-read sequence assembly of the gorilla genome. Science. 2016;352 6281:aae0344. doi:10.1126/science.aae0344.
2. Vij S, Kuhl H, Kuznetsova IS, Komissarov A, Yurchenko AA, Van Heusden P, et al. Chromosomal-Level Assembly of the Asian Seabass Genome Using Long Sequence Reads and Multi-layered Scaffolding. PLoS Genet. 2016;12 4:e1005954. doi:10.1371/journal.pgen.1005954.
3. Korlach J, Gedman G, Kingan SB, Chin CS, Howard JT, Audet JN, et al. De novo PacBio long-read and phased avian genome assemblies correct and add to reference genes generated with intermediate and short reads. Gigascience. 2017;6 10:1-16. doi:10.1093/gigascience/gix085.
4. Myers EW, Sutton GG, Delcher AL, Dew IM, Fasulo DP, Flanigan MJ, et al. A whole-genome assembly of *Drosophila*. Science. 2000;287 5461:2196-204. doi:10.1126/science.287.5461.2196.
5. Batzoglou S, Jaffe DB, Stanley K, Butler J, Gnerre S, Mauceli E, et al. ARACHNE: a whole-genome shotgun assembler. Genome Res. 2002;12 1:177-89. doi:10.1101/gr.208902.
6. Huang X, Wang J, Aluru S, Yang SP and Hillier L. PCAP: a whole-genome assembly program. Genome Res. 2003;13 9:2164-70. doi:10.1101/gr.1390403.
7. Margulies M, Egholm M, Altman WE, Attiya S, Bader JS, Bemben LA, et al. Genome sequencing in microfabricated high-density picolitre reactors. Nature. 2005;437 7057:376-80. doi:10.1038/nature03959.
8. Gnerre S, Maccallum I, Przybylski D, Ribeiro FJ, Burton JN, Walker BJ, et al. High-quality draft assemblies of mammalian genomes from massively parallel sequence data. Proc Natl Acad Sci U S A. 2011;108 4:1513-8. doi:10.1073/pnas.1017351108.
9. Luo R, Liu B, Xie Y, Li Z, Huang W, Yuan J, et al. SOAPdenovo2: an empirically improved memory-efficient short-read de novo assembler. Gigascience. 2012;1 1:18. doi:10.1186/2047-217X-1-18.
10. Chin CS, Peluso P, Sedlazeck FJ, Nattestad M, Concepcion GT, Clum A, et al. Phased diploid genome assembly with single-molecule real-time sequencing. Nat Methods. 2016;13 12:1050-4. doi:10.1038/nmeth.4035.
11. Koren S, Walenz BP, Berlin K, Miller JR, Bergman NH and Phillippy AM. Canu: scalable and accurate long-read assembly via adaptive k-mer weighting and repeat separation. Genome Res. 2017;27 5:722-36. doi:10.1101/gr.215087.116.
12. Kolmogorov M, Yuan J, Lin Y and Pevzner PA. Assembly of long, error-prone reads using repeat graphs. Nat Biotechnol. 2019;37 5:540-6. doi:10.1038/s41587-019-0072-8.
13. Li H. Minimap and miniasm: fast mapping and de novo assembly for noisy long sequences. Bioinformatics. 2016;32 14:2103-10. doi:10.1093/bioinformatics/btw152.
14. Ruan J and Li H. Fast and accurate long-read assembly with wtdbg2. bioRxiv. 2019.
15. Burton JN, Adey A, Patwardhan RP, Qiu R, Kitzman JO and Shendure J. Chromosome-scale scaffolding of de novo genome assemblies based on chromatin interactions. Nat Biotechnol. 2013;31 12:1119-25. doi:10.1038/nbt.2727.
16. Ghurye J, Pop M, Koren S, Bickhart D and Chin CS. Scaffolding of long read assemblies using long range contact information. BMC Genomics. 2017;18 1:527. doi:10.1186/s12864-017-3879-z.
17. Ghurye J and Pop M. Modern technologies and algorithms for scaffolding assembled genomes. PLoS Comput Biol. 2019;15 6:e1006994. doi:10.1371/journal.pcbi.1006994.
18. Howe K and Wood JM. Using optical mapping data for the improvement of vertebrate genome assemblies. Gigascience. 2015;4:10. doi:10.1186/s13742-015-0052-y.

19. Fierst JL. Using linkage maps to correct and scaffold de novo genome assemblies: methods, challenges, and computational tools. Front Genet. 2015;6:220. doi:10.3389/fgene.2015.00220.
20. Meyer A and Van de Peer Y. From 2R to 3R: evidence for a fish-specific genome duplication (FSGD). Bioessays. 2005;27 9:937-45. doi:10.1002/bies.20293.
21. Sacerdot C, Louis A, Bon C, Berthelot C and Roest Crollius H. Chromosome evolution at the origin of the ancestral vertebrate genome. Genome Biol. 2018;19 1:166. doi:10.1186/s13059-018-1559-1.
22. Gregory TR. Synergy between sequence and size in large-scale genomics. Nat Rev Genet. 2005;6 9:699-708. doi:10.1038/nrg1674.
23. Voss SR, Kump DK, Putta S, Pauly N, Reynolds A, Henry RJ, et al. Origin of amphibian and avian chromosomes by fission, fusion, and retention of ancestral chromosomes. Genome Res. 2011;21 8:1306-12. doi:10.1101/gr.116491.110.
24. Ruiz-Herrera A, Farre M and Robinson TJ. Molecular cytogenetic and genomic insights into chromosomal evolution. Heredity (Edinb). 2012;108 1:28-36. doi:10.1038/hdy.2011.102.
25. Irimia M, Tena JJ, Alexis MS, Fernandez-Minan A, Maeso I, Bogdanovic O, et al. Extensive conservation of ancient microsynteny across metazoans due to cis-regulatory constraints. Genome Res. 2012;22 12:2356-67. doi:10.1101/gr.139725.112.
26. Zimmermann B, Robert NSM, Technau U and Simakov O. Ancient animal genome architecture reflects cell type identities. Nat Ecol Evol. 2019;3 9:1289-93. doi:10.1038/s41559-019-0946-7.
27. Braasch I, Gehrke AR, Smith JJ, Kawasaki K, Manousaki T, Pasquier J, et al. The spotted gar genome illuminates vertebrate evolution and facilitates human-teleost comparisons. Nat Genet. 2016;48 4:427-37. doi:10.1038/ng.3526.
28. Ravi V and Venkatesh B. The Divergent Genomes of Teleosts. Annu Rev Anim Biosci. 2018;6:47-68. doi:10.1146/annurev-animal-030117-014821.
29. Nowoshilow S, Schloissnig S, Fei JF, Dahl A, Pang AWC, Pippel M, et al. The axolotl genome and the evolution of key tissue formation regulators. Nature. 2018;554 7690:50-5. doi:10.1038/nature25458.
30. Smith JJ, Timoshevskaya N, Timoshevskiy VA, Keinath MC, Hardy D and Voss SR. A chromosome-scale assembly of the axolotl genome. Genome Res. 2019;29 2:317-24. doi:10.1101/gr.241901.118.
31. Hellsten U, Harland RM, Gilchrist MJ, Hendrix D, Jurka J, Kapitonov V, et al. The genome of the Western clawed frog *Xenopus tropicalis*. Science. 2010;328 5978:633-6. doi:10.1126/science.1183670.
32. Sun YB, Xiong ZJ, Xiang XY, Liu SP, Zhou WW, Tu XL, et al. Whole-genome sequence of the Tibetan frog *Nanorana parkeri* and the comparative evolution of tetrapod genomes. Proc Natl Acad Sci U S A. 2015;112 11:E1257-62. doi:10.1073/pnas.1501764112.
33. Pokorna M, Giovannotti M, Kratochvil L, Caputo V, Olmo E, Ferguson-Smith MA, et al. Conservation of chromosomes syntenic with avian autosomes in squamate reptiles revealed by comparative chromosome painting. Chromosoma. 2012;121 4:409-18. doi:10.1007/s00412-012-0371-z.
34. Deakin JE and Ezaz T. Understanding the Evolution of Reptile Chromosomes through Applications of Combined Cytogenetics and Genomics Approaches. Cytogenet Genome Res. 2019;157 1-2:7-20. doi:10.1159/000495974.
35. Farre M, Kim J, Proskuryakova AA, Zhang Y, Kulemzina AI, Li Q, et al. Evolution of gene regulation in ruminants differs between evolutionary breakpoint regions and homologous synteny blocks. Genome Res. 2019;29 4:576-89. doi:10.1101/gr.239863.118.
36. Nanda I, Shan Z, Schartl M, Burt DW, Koehler M, Nothwang H, et al. 300 million years of conserved synteny between chicken Z and human chromosome 9. Nat Genet. 1999;21 3:258-9. doi:10.1038/6769.

37. Catchen JM, Conery JS and Postlethwait JH. Automated identification of conserved synteny after whole-genome duplication. Genome Res. 2009;19 8:1497-505. doi:10.1101/gr.090480.108.
38. Zhao T and Schranz ME. Network-based microsynteny analysis identifies major differences and genomic outliers in mammalian and angiosperm genomes. Proc Natl Acad Sci U S A. 2019;116 6:2165-74. doi:10.1073/pnas.1801757116.
39. Zhang G. The bird's-eye view on chromosome evolution. Genome Biol. 2018;19 1:201. doi:10.1186/s13059-018-1585-z.
40. Kim J, Larkin DM, Cai Q, Asan, Zhang Y, Ge RL, et al. Reference-assisted chromosome assembly. Proc Natl Acad Sci U S A. 2013;110 5:1785-90. doi:10.1073/pnas.1220349110.
41. Kolmogorov M, Armstrong J, Raney BJ, Streeter I, Dunn M, Yang F, et al. Chromosome assembly of large and complex genomes using multiple references. Genome Res. 2018;28 11:1720-32. doi:10.1101/gr.236273.118.
42. Koepfli KP, Paten B, Genome KCoS and O'Brien SJ. The Genome 10K Project: a way forward. Annu Rev Anim Biosci. 2015;3:57-111. doi:10.1146/annurev-animal-090414-014900.
43. Lewin HA, Robinson GE, Kress WJ, Baker WJ, Coddington J, Crandall KA, et al. Earth BioGenome Project: Sequencing life for the future of life. Proc Natl Acad Sci U S A. 2018;115 17:4325-33. doi:10.1073/pnas.1720115115.
44. Li H. Minimap2: pairwise alignment for nucleotide sequences. Bioinformatics. 2018;34 18:3094-100. doi:10.1093/bioinformatics/bty191.
45. Frith MC and Kawaguchi R. Split-alignment of genomes finds orthologies more accurately. Genome Biol. 2015;16:106. doi:10.1186/s13059-015-0670-9.
46. Kolmogorov M, Raney B, Paten B and Pham S. Ragout-a reference-assisted assembly tool for bacterial genomes. Bioinformatics. 2014;30 12:i302-9. doi:10.1093/bioinformatics/btu280.
47. Kapusta A, Suh A and Feschotte C. Dynamics of genome size evolution in birds and mammals. Proc Natl Acad Sci U S A. 2017;114 8:E1460-E9. doi:10.1073/pnas.1616702114.
48. Wang Z, Zhang J, Yang W, An N, Zhang P, Zhang G, et al. Temporal genomic evolution of bird sex chromosomes. BMC Evol Biol. 2014;14:250. doi:10.1186/s12862-014-0250-8.
49. Ozerov MY, Ahmad F, Gross R, Pukk L, Kahar S, Kisand V, et al. Highly Continuous Genome Assembly of Eurasian Perch (*Perca fluviatilis*) Using Linked-Read Sequencing. G3 (Bethesda). 2018;8 12:3737-43. doi:10.1534/g3.118.200768.
50. Feron R, Zahm M, Cabau C, Klopp C, Roques C, Bouchez O, et al. Characterization of a Y-specific duplication/insertion of the anti-Mullerian hormone type II receptor gene based on a chromosome-scale genome assembly of yellow perch, *Perca flavescens*. bioRxiv. 2019.
51. Walker BJ, Abeel T, Shea T, Priest M, Abouelliel A, Sakthikumar S, et al. Pilon: an integrated tool for comprehensive microbial variant detection and genome assembly improvement. PLoS One. 2014;9 11:e112963. doi:10.1371/journal.pone.0112963.
52. Simao FA, Waterhouse RM, Ioannidis P, Kriventseva EV and Zdobnov EM. BUSCO: assessing genome assembly and annotation completeness with single-copy orthologs. Bioinformatics. 2015;31 19:3210-2. doi:10.1093/bioinformatics/btv351.
53. Shafin K, Pesout T, Lorig-Roach R, Haukness M, Olsen HE, Bosworth C, et al. Efficient *de novo* assembly of eleven human genomes using PromethION sequencing and a novel nanopore toolkit. bioRxiv. 2019.

Supplementary tables

Supplementary Table 1: CSA results of the best case scenario, for representative genomes of mammals, birds and fish.

	vertebrate clade	Mammalia	Aves	Teleostei
CSA setup	species	*Homo sapiens*	*Taeniopygia guttata*	*Siniperca chuatsi*
	species haploid chr count=n	23	40	24
	input data type: seq. coverage; N50 read length	SMRT: 60-fold; N50:20 kb	SMRT: 96-fold; N50:19 kb	SMRT: 50-fold; N50:12 kb
	benchmark scenario	best case	best case	best case
	reference	*H.sapiens GRCh38*	*T.guttata*	*S. chuatsi*
	reference divergence time	0.0	0.0	0.0
	reference haploid chr count	23	~40	24
CSA step1	total contig length	2,846,783,372	1,099,596,476	721,123,858
	contig N50	15,637,873	17,162,677	11,615,497
	max. contig length	103,078,150	65,902,548	30,768,821
CSA step2	placed in top n chr	97.44%	93.99%	98.40%
	scaffold N50	151,200,468	71,399,975	30,139,544
	max. scaffold length	234,201,754	151,322,278	38,172,652
CSA step3	total contig length	2,849,859,767	1,096,751,084	721,496,232
	contig N50	25,894,807	26,171,859	13,428,662
	max. contig length	109,927,675	72,222,397	30,765,277
CSA final	total scaffold length	2,866,335,788	1,099,655,660	726,281,108
	total contig length	2,849,495,757	1,096,712,419	715,472,342
	placed in top n chr	97.50%	94.24%	99.33%
	scaffold N50	150,569,357	71,372,070	30,013,467
	contig N50	25,894,807	27,655,297	16,495,661
	max. scaffold length	233,785,065	151,378,290	38,160,875
	max. contig length	109,927,675	72,222,397	30,765,277
	runtime server (80 threads E7-8890v4@2.20GHz)	16h	5h:45m	2h:30m
	contig N50 improvement over CSA step1 [x-fold]	1.66	1.61	1.42
	contig N50 impr. over best published SMRT assembly [x-fold]	0.98	2.30	1.35
Errors scf	fusions; intra-chr. translocations; inversions (blocks >300kbp)	f:0; t:6; i:2;	f:0; t:1; i:4	f:0(1); t:1; i:5(6)
Errors ctg		f:0; t:2; i:0	f:0; t:1; i:4	f:0(1); t:0; i:3(4)

Supplementary Table 2: CSA results using divergent reference genomes

CSA setup	**species**	*Homo sapiens*	*Homo sapiens*	*Homo sapiens*	*Homo sapiens*	*Taeniopygia guttata*	*Taeniopygia guttata*	*Taeniopygia guttata*	*Siniperca chuatsi*	*Siniperca chuatsi*
	**species haploid chr count=n**	23	23	23	23	40	40	40	24	24
	**input data type: seq. coverage; N50 read length**	SMRT: 60-fold; N50:20 kb	SMRT: 60-fold; N50:20 kb	SMRT: 60-fold; N50:20 kb	SMRT: 60-fold; N50:20 kb	SMRT: 96-fold; N50:19 kb	SMRT: 96-fold; N50:19 kb	SMRT: 96-fold; N50:19 kb	SMRT: 50-fold; N50:12 kb	SMRT: 50-fold; N50:12 kb
	**benchmark scenario**	diverged ref.	diverged ref.	diverged ref.	diverged ref.	diverged reference	diverged reference	diverged draft assembly	diverged reference	draft contig reference
	**reference**	*P. abelii*	*C. jacchus*	*L. canadensis*	*O. anatinus*	*C. anna*	*G. gallus*	*A. mississipiensis*	*P. flavescens*	*P. fluviatilis* (CSA step1 contigs; N50: 2.8Mbp)
	**reference divergence time**	15.8	42.9	94.0	180.0	65.0	80.0	240.0	65.0	65.0
	**reference haploid chr count**	24	22	19	27	>40	~39	16	24	24
CSA step1	**total contig length**	2,846,783,372	2,846,783,372	2,846,783,372	2,846,783,372	1,099,596,476	1,099,596,476	1,099,596,476	721,123,858	721,123,858
	**contig N50**	15,637,873	15,637,873	15,637,873	15,637,873	17,162,677	17,162,677	17,162,677	11,615,497	11,615,497
	**max. contig length**	103,078,150	103,078,150	103,078,150	103,078,150	65,902,548	65,902,548	65,902,548	30,768,821	30,768,821
CSA step2	**placed in top n chr**	93.76%	96.00%	95.83%	92.51%	92.04%	92.77%	90.48%	93.92%	76.04%
	**scaffold N50**	128,353,462	129,800,600	167,706,686	127,367,631	55,664,037	74,226,408	53,874,258	29,495,621	22,961,239
	**max. scaffold length**	219,829,795	210,205,854	392,920,739	307,297,870	146,901,896	186,662,268	115,106,231	38,170,751	36,597,929
CSA step3	**total contig length**	2,849,302,170	2,848,630,351	2,848,648,159	2,849,085,066	1,096,419,901	1,096,584,403	1,096,413,559	721,051,678	720,977,241
	**contig N50**	25,506,235	24,655,207	25,316,480	24,550,351	18,624,647	19,226,526	18,624,651	14,142,781	13,428,662
	**max. contig length**	106,332,896	106,583,100	104,396,992	108,663,965	65,887,603	65,887,602	65,887,602	30,764,502	30,764,502
CSA final	**total scaffold length**	2,858,013,933	2,856,072,174	2,856,023,627	2,857,274,643	1,097,447,182	1,097,814,908	1,098,232,426	724,064,917	721,968,017
	**total contig length**	2,849,099,702	2,848,553,253	2,848,553,748	2,849,048,046	1,096,370,479	1,096,530,772	1,096,361,835	721,014,191	720,954,856
	**placed in top n chr**	93.84%	96.07%	96.10%	91.62%	92.13%	93.11%	90.56%	94.73%	77.26%
	**scaffold N50**	127,999,133	129,879,741	167,642,951	127,324,216	55,672,797	74,887,648	53,831,915	29,407,777	23,439,636
	**contig N50**	29,334,513	24,749,570	28,128,276	25,651,180	18,882,724	19,248,331	19,226,897	16,688,192	16,688,192
	**max. scaffold length**	219,825,495	210,340,950	393,325,976	307,230,484	146,917,470	186,491,133	114,959,208	38,156,639	36,593,613
	**max. contig length**	109,935,533	106,583,100	104,396,992	108,663,965	65,887,603	65,887,602	65,887,602	35,806,844	35,807,018
	**runtime server (80 threads E7-8890v4@2.20GHz)**	15h30m	13h:30m	13h	12h:40m	5h:41m	5h:46m	5h:52m	2h:30m	2h:20m
	**contig N50 improvement over CSA step1 [x-fold]**	1.88	1.58	1.80	1.64	1.10	1.12	1.12	1.44	1.44
	**contig N50 impr. over best published SMRT assembly [x-fold]**	1.12	0.94	1.07	0.98	1.57	1.60	1.60	1.37	1.37
Errors scf	**fusions; intra-chr. translocations; inversions (blocks >300kbp)**	f:0; t:58; i:8	f:11; t:72; i:12	f:15; t:78; i:12	f:47; t:87; i:23	f:1; t:33; i:20	f:2; t:26; i:20	f:2; t:22; i:18	f:1(2); t:13; i:16(17)	f:0(1); t:2; i:9(10)
Errors ctg		f:0; t:4; i:6	f:0; t:4; i:7	f:0; t:5; i:10	f:1; t:5; i:12	f:0; t:1; i:6	f:0; t:1; i:8	f:0; t:1; i:6	f:0(1); t:0; i:5(6)	f:0(1); t:0; i:5(6)

Supplementary Table 3: ONT read assembly supported by 10X Genomics, genus-level and order-level references.

	**vertebrate clade**	Teleostei	Teleostei	Teleostei	Teleostei
CSA setup	**species**	*Perca fluviatilis*	*Perca fluviatilis*	*Perca fluviatilis*	*Perca fluviatilis*
	**species haploid chr count=n**	24	24	24	24
	**input data type: seq. coverage; N50 read length**	ONT: 67-fold; N50: 12 kb	ONT: 67-fold; N50: 12 kb	ONT: 67-fold; N50: 12 kb	ONT: 67-fold; N50: 12 kb
	**benchmark scenario**	best case	10X genomics + two diverged references	two diverged references	diverged reference
	**reference**	*Perca fluviatilis HiC*	*10X P.fluviatilis; P. flavescens; S.chuatsi*	*P. flavescens; S.chuatsi*	*S.chuatsi*
	**reference divergence time**	0.0	0,0; 10-20; 65;	10-20; 65;	65.0
	**reference haploid chr count**	24	24	24	24
CSA step1	**total contig length**	929,045,493	929,045,493	929,045,493	929,045,493
	**contig N50**	2,816,703	2,816,703	2,816,703	2,816,703
	**max. contig length**	15,906,396	15,906,396	15,906,396	15,906,396
CSA step2	**placed in top n chr**	95.89%	93.89%	93.10%	86.12%
	**scaffold N50**	38,622,337	37,697,155	37,043,440	33,865,843
	**max. scaffold length**	47,653,006	47,519,747	46,927,054	46,802,501
CSA step3	**total contig length**	929,510,545	929,018,531	929,069,033	928,502,336
	**contig N50**	5,237,589	4,684,879	5,007,526	4,699,930
	**max. contig length**	24,199,483	24,200,308	24,198,405	24,792,848
CSA final	**total scaffold length**	944,861,078	943,065,049	942,130,566	936,090,143
	**total contig length**	929,241,819	928,740,496	928,809,152	928,301,535
	**placed in top n chr**	96.15%	94.28%	93.79%	86.34%
	**scaffold N50**	38,362,302	37,309,771	36,955,123	33,805,383
	**contig N50**	7,574,134	8,008,293	7,745,610	7,050,314
	**max. scaffold length**	47,026,637	46,017,986	46,666,870	46,417,396
	**max. contig length**	27,361,245	27,581,946	27,582,002	27,361,483
	**runtime server (80 threads E7-8890v4@2.20GHz)**	5h:02m	5h:30m	5h:15m	5h:15m
	**contig N50 improvement over CSA step1 [x-fold]**	2.69	2.84	2.75	2.50
	**contig N50 impr. over best published ONT assembly [x-fold]**	2.92	3.09	2.99	2.72
Errors scf	**fusions; intra-chr. translocations; inversions (blocks >300kbp)**	f:0; t:0; i:3	f:0; t:12; i:13	f:0; t:19; i:15	f:0; t:57; i:16
Errors ctg		f:0; t:0; i:1	f:0; t:0; i:4	f:0; t:0; i:3	f:0; t:0; i:4

Supplementary Table 4: Influence of sequencing coverage on *H. sapiens* CSA assemblies.

	vertebrate clade	Mammalia	Mammalia	Mammalia	Mammalia	Mammalia
CSA setup	species	*Homo sapiens*	*Homo sapiens*	*Homo sapiens*	*Homo sapiens*	*Homo sapiens*
	species haploid chr count=n	23	23	23	23	23
	input data type: seq. coverage; N50 read length	SMRT: 60-fold; N50:20 kb	SMRT: 40-fold; N50:20 kb	SMRT: 30-fold; N50:20 kb	SMRT: 20-fold; N50:20 kb	SMRT: 15-fold; N50:20 kb
	benchmark scenario	best case	lower coverage	lower coverage	lower coverage	lower coverage
	reference	*H.sapiens GRCh38*	*H.sapiens GRCh38*	*H.sapiens GRCh38*	*H.sapiens GRCh38*	*H.sapiens GRCh38*
	reference divergence time	0.0	0	0.0	0.0	0.0
	reference haploid chr count	23	23	23	23	23
CSA step1	total contig length	2,846,783,372	2,840,640,553	2,841,463,516	2,847,406,287	2,826,278,103
	contig N50	15,637,873	16,341,163	12,497,249	4,383,406	1,299,535
	max. contig length	103,078,150	88,948,008	82,332,068	27,279,889	11,252,181
CSA step2	placed in top n chr	97.44%	97.60%	97.96%	96.85%	96.80%
	scaffold N50	151,200,468	151,424,426	151,231,434	142,490,860	143,351,851
	max. scaffold length	234,201,754	235,703,142	234,220,726	235,695,545	236,751,258
CSA step3	total contig length	2,849,859,767	2,843,263,701	2,841,991,360	2,851,115,509	2,847,893,417
	contig N50	25,894,807	25,692,063	24,108,925	13,125,096	4,226,428
	max. contig length	109,927,675	94,304,511	105,741,764	77,076,239	23,200,772
CSA final	total scaffold length	2,866,335,788	2,857,919,940	2,858,673,516	2,869,385,805	2,879,298,236
	total contig length	2,849,495,757	2,842,762,436	2,841,530,625	2,850,195,964	2,846,708,724
	placed in top n chr	97.50%	97.66%	97.96%	97.00%	96.83%
	scaffold N50	150,569,357	150,914,039	151,221,074	142,388,744	143,182,554
	contig N50	25,894,807	29,383,620	26,652,963	16,727,580	5,840,632
	max. scaffold length	233,785,065	235,400,774	233,997,714	235,967,303	236,759,292
	max. contig length	109,927,675	94,304,511	105,741,764	110,922,859	36,670,033
	runtime server (80 threads E7-8890v4@2.20GHz)	16:00h	11h:26m	10h:45	7h:49m	7h:04m
	contig N50 improvement over CSA step1 [x-fold]	1.66	1.80	2.13	3.82	4.49
	contig N50 impr. over best published SMRT assembly [x-fold]	0.98	1.12	1.01	0.64	0.22
Errors scf	fusions; intra-chr. translocations; inversions (blocks >300kbp)	f:0; t:6; i:2	f:0; t:14; i:1	f:0; t:6; i:0	f:2; t:14; i:2	f:4; t:7; i:1
Errors ctg		f:0; t:2; i:0	f:0; t:1; i:3	f:0; t:0; i:0	f:2; t:4; i:3	f:2; t:4; i:3

	vertebrate clade	Mammalia	Mammalia	Mammalia	Mammalia	Mammalia
CSA setup	species	*Homo sapiens*	*Homo sapiens*	*Homo sapiens*	*Homo sapiens*	*Homo sapiens*
	species haploid chr count=n	23	23	23	23	23
	input data type: seq. coverage; N50 read length	SMRT: 60-fold; N50:20 kb	SMRT: 40-fold; N50:20 kb	SMRT: 30-fold; N50:20 kb	SMRT: 20-fold; N50:20 kb	SMRT: 15-fold; N50:20 kb
	benchmark scenario	diverged ref	lower coverage / div.ref	lower coverage / div.ref	lower coverage / div.ref	lower coverage / div.ref
	reference	*P. abelii*	*P. abelii*	*P. abelii*	*P. abelii*	*P. abelii*
	reference divergence time	15.8	15.76	15.8	15.8	15.8
	reference haploid chr count	24	24	24	24	24
CSA step1	total contig length	2,846,783,372	2,840,640,553	2,841,463,516	2,847,406,287	2,826,278,103
	contig N50	15,637,873	16,341,163	12,497,249	4,383,406	1,299,535
	max. contig length	103,078,150	88,948,008	82,332,068	27,279,889	11,252,181
CSA step2	placed in top n chr	93.76%	95.17%	95.33%	94.29%	94.40%
	scaffold N50	128,353,462	130,971,511	131,070,274	131,625,032	132,314,504
	max. scaffold length	219,829,795	218,626,822	220,361,819	222,667,500	246,425,011
CSA step3	total contig length	2,849,302,170	2,843,047,974	2,841,357,350	2,850,745,354	2,846,782,940
	contig N50	25,506,235	25,690,650	19,077,358	12,527,629	4,187,987
	max. contig length	106,332,896	110,075,087	105,740,926	77,028,376	29,369,898
CSA final	total scaffold length	2,858,013,933	2,851,752,214	2,850,589,323	2,861,981,207	2,873,174,020
	total contig length	2,849,099,702	2,842,870,217	2,841,215,149	2,850,328,062	2,845,954,290
	placed in top n chr	93.84%	95.34%	95.45%	94.17%	94.52%
	scaffold N50	127,999,133	130,814,023	130,998,612	131,456,531	132,067,157
	contig N50	29,334,513	25,696,829	25,502,124	16,322,611	5,493,827
	max. scaffold length	219,825,495	218,621,187	219,807,659	222,045,937	244,800,533
	max. contig length	109,935,533	110,075,087	105,740,926	110,874,709	41,099,382
	runtime server (80 threads E7-8890v4@2.20GHz)	15h30m	15h:03m	13h:28m	12h:41m	12h:15m
	contig N50 improvement over CSA step1 [x-fold]	1.88	1.57	2.04	3.72	4.23
	contig N50 impr. over best published SMRT assembly [x-fold]	1.12	0.98	0.97	0.62	0.21
Errors scf	fusions; intra-chr. translocations; inversions (blocks >300kbp)	f:0; t:58; i:8	f:1; t:56; i:12	f:0; t:65; i:8	f:6; t:68; i:7	f:4; t:67; i:13
Errors ctg		f:0; t:4; i:6	f:0; t:5; i:11	f:0; t:8; i:7	f:4; t:8; i:8	f:2; t:6; i:6

Supplementary Table 5: *H. sapiens* CSA assembly by ultra long reads.

	**vertebrate clade**	Mammalia
CSA setup	**species**	*Homo sapiens*
	**species haploid chr count=n**	23
	**input data type: seq. coverage; N50 read length**	ONT: 50-fold; N50:70 kb
	**benchmark scenario**	best case
	**reference**	*H. sapiens*
	**reference divergence time**	0.0
	**reference haploid chr count**	23
CSA step1	**total contig length**	2,890,906,026
	**contig N50**	38,151,541
	**max. contig length**	109,306,044
CSA step2	**placed in top n chr**	96.58%
	**scaffold N50**	150,526,033
	**max. scaffold length**	235,656,620
CSA step3	**total contig length**	2,883,898,281
	**contig N50**	39,712,896
	**max. contig length**	109,194,274
CSA final	**total scaffold length**	2,891,827,299
	**total contig length**	2,883,411,943
	**placed in top n chr**	96.69%
	**scaffold N50**	150,158,625
	**contig N50**	48,445,356
	**max. scaffold length**	236,106,895
	**max. contig length**	109,194,274
	**runtime server (80 threads E7-8890v4@2.20GHz)**	23h:43m
	**contig N50 improvement over CSA step1 [x-fold]**	1.27
	**contig N50 impr. over best published SMRT assembly [x-fold]**	1.84
Errors scf	**fusions; intra-chr. translocations; inversions (blocks >300kbp)**	f:0; t:9; i:2
Errors ctg		f:0; t:0; i:2

Supplementary figures:

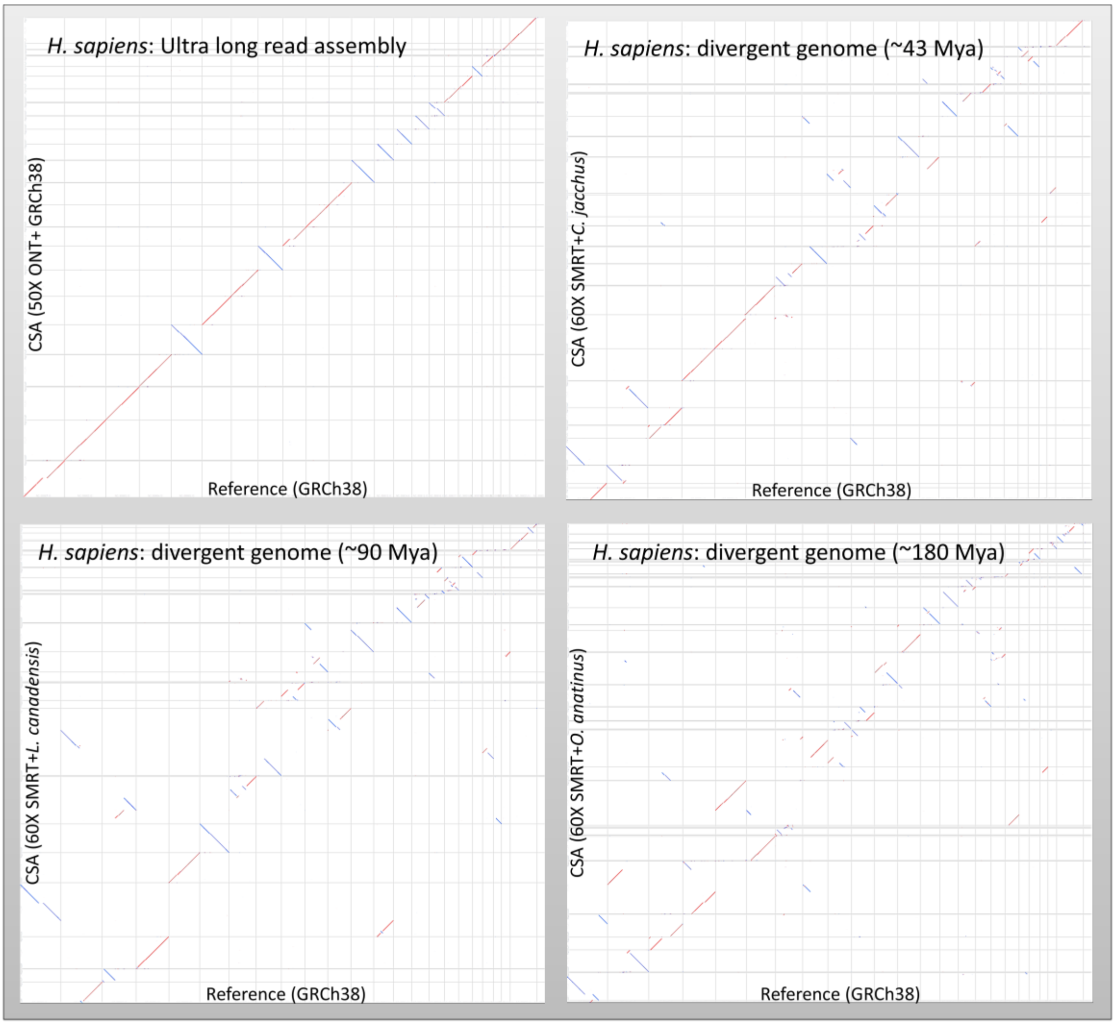

Supplementary Figure 1: Additional dot plots for *H. sapiens* CSA assemblies using ONT Ultra long reads, or SMRT reads and more diverged reference genomes.

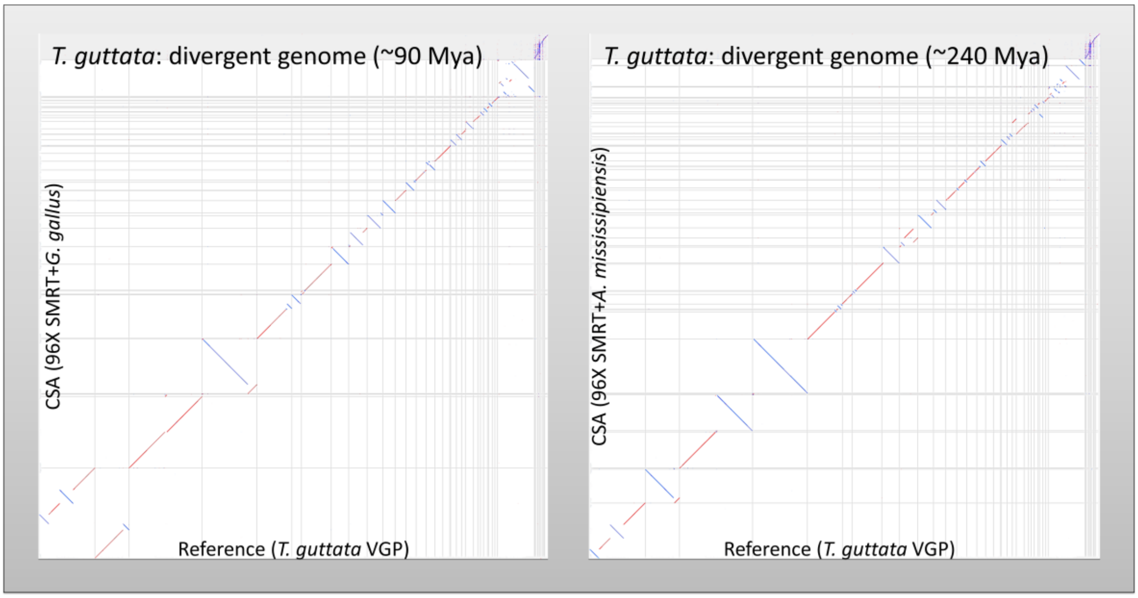

Supplementary Figure 2: Additional dot plots for *T. guttata* CSA assemblies using SMRT reads and more diverged reference genomes.

Additional file 1

Click here to access/download
Supplementary Material
CSA-Eval table_version6.xlsx

Additional file 2

Click here to access/download
Supplementary Material
CSA-Figures_HiRes.pptx

Additional file 3 + 4 download site

Click here to access/download
Supplementary Material
CSA2.6-DOWNLOAD.pdf
