# Supplementary material for: CSA: A high-throughput chromosome-scale assembly pipeline for vertebrate genomes
Source: Gigascience. 2020 May 25;9(5):giaa034. doi: 10.1093/gigascience/giaa034 (PMC7247394; doi:10.1093/gigascience/giaa034)
Supplement: giaa034_GIGA-D-19-00380_Revision_1 [file giaa034_giga-d-19-00380_revision_1.pdf]

GigaScience

CSA: A high-throughput chromosome-scale assembly pipeline for vertebrate genomes

--Manuscript Draft--

Manuscript Number:	GIGA-D-19-00380R1
Full Title:	CSA: A high-throughput chromosome-scale assembly pipeline for vertebrate genomes
Article Type:	Technical Note
Funding Information:	Deutsche Forschungsgemeinschaft (KU 3596/1-1; project number: 324050651) — Dr. Heiner Kuhl
Abstract:	Background: Easy-to-use and fast bioinformatics pipelines for long-read assembly that go beyond the contig-level to generate high-quality chromosome-scale genomes from raw data remain scarce.<br>Results: Chromosome Scale Assembler (CSA) is a novel computationally highly efficient bioinformatics pipeline that fills this gap. CSA integrates information from scaffolded assemblies (e.g. Hi-C or 10X Genomics) or even from diverged reference genomes into the assembly process. As CSA performs automated assembly of chromosome-sized scaffolds, we benchmark its performance against state-of-the art reference genomes that have been built in a laborious fashion using multiple separate assembly tools and manual curation. CSA increases the contig length using scaffolding, local re-assembly and gap-closing. On certain datasets, initial contig N50 may be increased up to 4.5-fold. For smaller vertebrate genomes, chromosome-scale assemblies can be achieved within 12 h using low cost, high-end desktop computers. Mammalian genomes can be processed within 16 h on compute-servers. Using diverged reference genomes for fish, birds and mammals, we demonstrate that CSA calculates chromosome-scale assemblies from long-read data and genome comparisons alone. Even contig-level draft assemblies of diverged genomes are helpful for reconstructing chromosome-scale sequences. CSA is also capable of assembling ultra-long reads.<br>Conclusions: CSA can speed-up and simplify chromosome-level assembly and significantly lower costs of large-scale family-level vertebrate genome projects.
Corresponding Author:	Heiner Kuhl<br>IGB Leibniz-Institute of Freshwater Ecology and Inland Fisheries<br>Berlin, Berlin GERMANY
Corresponding Author Secondary Information:	
Corresponding Author's Institution:	IGB Leibniz-Institute of Freshwater Ecology and Inland Fisheries
Corresponding Author's Secondary Institution:	
First Author:	Heiner Kuhl
First Author Secondary Information:	
Order of Authors:	Heiner Kuhl
	Ling Li
	Sven Wuertz
	Matthias Stöck
	Xu-Fang Liang
	Christophe Klopp
Order of Authors Secondary Information:	
Response to Reviewers:	Dear Dr. Edmunds,<br><br>we herewith submit our revised manuscript “CSA: A high-throughput chromosome-scale assembly pipeline for vertebrate genomes” for your consideration.

Powered by Editorial Manager® and ProduXion Manager® from Aries Systems Corporation

We tried to answer all open questions and changed the manuscript accordingly. In this regard, we would like to thank the reviewers and editor for their valuable comments. As requested CSA has now been registered to “bio.tools” and “SciCrunch.org” databases.

Regarding the benchmarking of CSA, we have discussed other options among the authors but would like to stay with comparing results of CSA to state-of-the-art reference genome assemblies, which represent the best assemblies currently available for the different species presented in the manuscript.

We would like to do so for mainly three reasons:

a)There is currently no pipeline for long-read assembly published that is comparable to CSA. Similar pipelines are only available for short-read assembly (e.g. IMAP proposed by Reviewer 1).

b)CSA is a bioinformatics pipeline that automates many steps (4 to 5 different tasks) necessary to arrive at chromosomal-scale assemblies. For each of these tasks many different software tools are available and testing all kinds of combinations of these tools in comparison to CSA would be out of scope of the manuscript and very puzzling to the reader.

c)It would be easy to choose a set of programs which chained would perform less well than CSA and such a benchmark would be meaningless, while comparison with reference genomes is much more reliable.
We are convinced that our benchmarking system clearly shows that CSA produces results that are very close to state-of-the-art reference genomes and at the same time reduces costs for input data types, computational resources and manual curation time.

We hope you may consider our manuscript to be published in Gigascience as a Technical Note.

Yours sincerely,

Dr. Heiner Kuhl
On behalf of all authors

The authors’ answers to reviewer reports are marked by "R:"

Reviewer #1: The paper presents a new pipeline for genome assembly. This pipeline uses reference genome, gap filling and assembly correction, for the production of the final assembly. The pipeline includes tools with a low memory footprint (relatively to genome assembly tools).
The paper is clearly written, and 6 use cases, which provide metrics on several scenarios, are presented.
This work should be useful for many lab around the world.
I have tried the example available on the GitHub repository. However, due to limited time to review this article (2 weeks), I did not have time to redo the analyses done in the article.
The package is easy to install and I could run seamlessly the small example.
However, I have major comments on the manuscript.
I feel that a description of the state of the art is missing.
When reading the paper, the user may wonder whether:

1. the pipeline deliver better results that other methods,

R:
We understand that the editor and both reviewers would like to see comparisons to existing pipelines that do a similar job as CSA. However, to our knowledge, if such pipelines do already exist, they have not been published. Reviewer 1 proposed IMAP, but we found that this pipeline was created for short read assembly, while CSA was created for long read assembly.
We could, in principle, combine other existing tools that solve parts of the chromosome-level assembly problem for comparisons (see table below), but this would

Powered by Editorial Manager® and ProduXion Manager® from Aries Systems Corporation

actually mean constructing another new pipeline from scratch. If understood like this, there would be many combinations of several kinds of tools… However, we strongly believe that benchmarking would be out of scope (as reviewer 1, already mentioned).

To solve these issues, we have compared CSA results to published current state-of-the-art reference genome assemblies (horizontal axis of each dot plot / contig N50 improvements mentioned in text and suppl. tables), which have in most cases been obtained from many different sequencing and mapping techniques and substantial manual curation efforts. This type of benchmarking shows that CSA can deliver similar or even better results (regarding contig N50) with much less costs, efforts and at high-throughput level.

We now do explain our choice of benchmarking in the manuscript as follows (L194 – L199):
"In the following, we tested CSA on different scenarios and benchmark its performance. CSA automatically chains many steps that traditionally require different software tools and laborious manual curation, for which similar pipelines are currently only available for short-read assembly (IMAP[44]). Therefore, we do not compare CSA-results with known contig-level genome assembly tools or other software packages solving only parts of chromosomal assemblies, but by re-assembling the currently best chromosomal-scale reference genomes in different vertebrate species."

To underline our benchmarking we have now added Table 1 to the manuscript, which directly compares important statistical values of the state-of-the-art reference genomes and "CSA + diverged genome" assemblies.

2. the selected features (gap filling, assembly correction, etc.) are the best mix (i.e. why did you choose these features over others?),

R:
We now have improved this and discuss our choices in the manuscript (see below for details regarding the different alternative tools that the reviewers proposed).

3. the selected tools (implementing the features) have been carefully selected.

R:
As above, we explain our choices now (see below).

Objective 1 has been partly addressed. However, I think that the other objectives deserve at least a discussion (an exhaustive benchmark may be out of scope).

Main questions:
- Concerning objective 1:
  + Please compare your approach/results with IMAP.

R: Thanks for hinting us at IMAP, which is a pipeline with similar features as CSA but it can only handle short reads. To address this point, we now cite IMAP in the Introduction (L117-L118) and mention it as being similar to CSA (L196 - L197). Unfortunately, IMAP it is not able to use long read data and thus cannot be directly compared with CSA.

- Concerning objective 2:
  + Why did you not include other tools, such as polishers? You mentionned MEDAKA and PILON in scenario 3. If you did not include them in the pipeline, please mention why.

R:
The reviewer is right; consensus polishing is a crucial step after assembling noisy long read data. A variety of methods are available. We think an in-depth analysis of consensus polishing methods would be a manuscript of its own and we would like to focus on chromosomal-level assembly in our manuscript. Nevertheless, we do now state more clearly that CSA currently does not perform polishing (L191 - L193):
"The current version of CSA does not include methods for consensus polishing, yet, one iteration of consensus polishing using long-reads and two iterations using short-

reads should be applied prior to sequence annotation efforts."

- Concerning objective 3:
  + Please discuss the use of RAGOUT (by the way, why not RAGOUT 2?), compared to other tools, such as MeDuSa or RACA.

R:
We have long experience using RAGOUT1 for constructing chromosomal-scale assemblies using related reference genomes (see for example burrowing owl genome; PMID: 29769357). We have also tested RAGOUT2 within CSA, but found that it produced slightly less complete chromosomal reconstructions (few percent) in this setting. RAGOUT2 by default also breaks assemblies and fits them to the reference, although this may be helping to remove missassemblies when using closely related reference genomes, it may also remove true rearrangements between genomes, thus we decided to stay with RAGOUT1. RAGOUT1 or 2 support maf alignment format, which made it easy to combine it with the fast and at the same time sensitive LAST aligner. We now briefly discuss this issue in the new version of the manuscript.

We are convinced that we cannot use MeDuSa, as it relies on Mummer, which is not a sensitive and fast aligner for whole genome alignment of large, diverged genomes.

Likewise, we do not use RACA, because it is a very complex pipeline consisting of many scripts. It is not able to use multiple references in parallel and it is by default using slower alignment methods (here „LASTZ", while CSA uses „LAST aligner", a completely different tool despite similar name). Again, we now briefly discuss this point in the new version of the manuscript.

Taken together, we mention these tools now in the section "Implementation of the CSA pipeline" L180 - L183 and explain why we think it is more appropriate to stay with RAGOUT1.

  + Some tools have been developed for CSA: breaking contigs, joining contigs, closing gaps. Why did you implement these features, whereas solutions already exist: SMSC or BIGMAC for breaking contigs, LINKS for scaffolding, LR_Gapcloser or GAPPader for closing gaps? These tools have been published and benchmarked (to some extent). Do your tools perform better?

R:
SMSC: Has not been tested on vertebrate genomes, just E. coli and Yeast. Possibly performance issues may exist for vertebrate-sized genomes (as it is using outdated aligners like NUCMER or BLASR by default).
BIGMAC: this has been designed for metagenomes but not for genomes, running 2 h with 20 threads on small assemblies. BIGMAC on large genomes would possibly take longer than running the whole CSA pipeline. To address this, we do now discuss assembly reconciliation in L171 - L179.

LINKS: We have tested LINKS a while ago, but were not happy with its performance on large genomes. The alignment-free kmer analysis implemented in LINKS cannot be parallelized and is actually much slower than current multi-threaded mapping tools like minimap2 on large datasets.

LR_GAPCLOSER and also PBJelly: in many cases appears inefficient, as long neighboring contig overlaps remain unclosed, such unresolved repeats are typical for long read assemblies.
In response, we discuss this now in L188 - L191.

GAPPADER: This tool uses paired-end or mate-pair short reads for gap closure, it cannot be used for gap closure by long reads.

Other questions:
 - Did you consider using Nextflow or Snakemake? This would probably help the users who work on a cluster for an easier interface with a job scheduler.

Powered by Editorial Manager® and ProduXion Manager® from Aries Systems Corporation

R:
We thank the reviewer for this suggestion and have discussed this among the authors. CSA, as most current long-read assembly tools, depends on a single computing node with high amount of RAM and CPU power, running jobs distributed on a cluster would not result in much better performance as there is little room for parallelization of the pipeline. This lowers our interest of using a job scheduler system. Anyway, we will consider adapting the pipeline to improve usability (e.g. easy re-start of stopped processes etc.) in future versions.

- I do not exactly understand why, on Scenario 5, the assembly is not perfect, even with low coverage, given that the very same genome is given as reference (as far as I understand it). Why is not LAST+RAGOUT stitching contigs?

R:
The assembly is imperfect, because with lower coverage more miss-assemblies occur in the primary assembly and slip through the miss-assembly correction. CSA does not split the primary contigs according to the reference, because otherwise species-specific rearrangements would be removed from the assemblies.

The reviewer is right that RAGOUT can close gaps, but RAGOUT was designed to close gaps based on exact sequence matches between neighboring contig ends, as these occur in de bruijn graph short-read assemblies. With long-read read assemblies, neighboring contig overlaps are typically noisy (often even partly uncorrected reads), thus RAGOUT cannot close these gaps.
This is now explained in the main text (L184 - L187)

- Scenario 6, do you compare ULR+CSA+GRCh38 to ULR+SHASTA? If so, since you provide the "true" genome, is not the first method highly favored?

R:
This benchmark was mainly to show that wtdbg2 and thus CSA can be used to assemble ULRs, which was questioned by the SHASTA manuscript. We now do also provide information on an ULR assembly using not the “true” genome, but a diverged reference genome(L413 - L416).

- Could you please provide "meta-paramaters" to your tool? In minimap2, the author use the "-x" paramter. You could adopt something similar to handle Sequel reads.

R:
In the newest version of CSA parameter –l can be used to parametrize WTDBG2 in more detail, we give examples for sequel reads or ULRs in the Readme file. A preset for different input data will be implemented in future versions.

Minor questions and comments:
- Page 3, there is probably something missing in the sentence: "[...] huge genome sizes of amphibians pose strong exceptions: c-value [...]"

R:
We changed this to: "[...] in this regard the huge genome sizes of amphibians pose strong exceptions: c-value [...]"

- Page 3, what are "bird chromosomes"?

R:
To be clearer we have now changed this to:
“Nevertheless, ortholog genes that exhibit distinct order in bird chromosomes are also discretely ordered in the single well-assembled urodelan (Ambystoma mexicanum)”

- Figure 1, the tool names written in the right hand of the rectangles are too small. Please enlarge.

Powered by Editorial Manager® and ProduXion Manager® from Aries Systems Corporation

R:
We have doubled the font size.

- Figure 1, some rectangle texts start with a capital letter, some without. Why?

R:
We have unified the capital letters in the figure

- Figure 2, please present the figures in the caption. The reader may not be familiar with minidot outputs. Please explain the meaning of:
  + the vertical and horizontal thin lines,
  + the color of the diagonal lines.

R:
We now explain the plot properties in the caption of figure 2.

- Figure 2, please enlarge the dot plots, with at most two plots per row, preferably one.

R:
We have now adjusted the Figure 2 into two plots per row, which results in a full-page figure. All other options would take too much space. We think the full-sized plots could be better viewed downloading the supplement.

- Page 10, the commas seem misplaced in "Overall the fraction of consensus sequences assigned to the top n scaffolds, was slightly lower than [...]"

R:
We have removed the commas.

- Scenario 3, please briefly present the data used in the "10X Genomics assembly," such as sequencing technology and coverage.

R:
We added some basic values next to the citation: "….(68X Illumina short-read coverage, N50 contig / scaffold length 18.3 kbp / 6.3 Mbp according to [58])."

- Page 12, the comma seems superfluous in "To test, if CSA could reach [...]"

R:
We removed the comma.

- Page 12, please explain "C: 95.9%, F: 2.1%, M: 2.0%, n: 4584"

R:
We replaced the BUSCO shortcuts by full description:
"(Actinopterygii dataset, complete genes: 95.9%, fragmented genes: 2.1%, missing genes: 2.0%, number of tested genes: 4584)"

- Page 13, I do not understand the comma in "Benchmark scenario 4: CSA using draft assemblies as reference, contig-level assemblies of diverged species may be highly complementary." It is a semi-colon?

R:
As suggested we replaced the comma by a semicolon

- Other acronyms:
  + interchr.: inter-chromosomal
  + intrachr.: intra-chromosomal
  + f: inter-chromosomal fusion
  + t: intra-chromosomal translocation
  + i: inversion
  + Hs: Homo sapiens

Powered by Editorial Manager® and ProduXion Manager® from Aries Systems Corporation

+ Tg: taoniopygia guttat
+ Sc: Siniperca chuatsi
+ RAM: random access memory

R:
Thank you very much. We added these to the Abbreviation section.

- Page 17, in "Programming language", you should probably add "awk."

R:
We added AWK.

- Page 20 and after, please increase font for your commands.

R:
We have set the Font to Courier 10 now, line breaks are indicated now by "\" thus one could directly copy paste the commands into a linux terminal.

- Page 21, "comma separated list (e.g." -> "(e.g."

R:
We removed the whitespace letter.

- Page 23, please explain the "-p" and the "-l" parameters.

R:
We added the following two explanations:
"CSA uses the "-p" option to set the WTDBG2 consensus caller (0 = wtdbg-cons (default); 1 = wtpoa-cons; consensus calculation in step 1; 2 = wtdbg-cons -S 0; wtpoa-cns is slower but a bit more accurate; wtdbg-cns with option -S 0 is more stable on very long reads."

"CSA uses the –l "…" parameter to pass on detailed parameters to the wtdbg2 assembler. Parameters provided by –l "…" may overrule other wtdbg2 parameters which are set by CSA (e.g. -k, -s, -e or -m)."

Reviewer #2: Dear Colleagues,

I read with interest your manuscript on CSA, an assembly pipeline which uses synteny conservation across species. As you explain in the introduction, as massive vertebrate genome sequencing starts within the VGP project, not all genomes will get the same amount of attention, and such a pipeline would be useful in evening out the quality of assemblies.
I really liked the sequence of benchmarks, each with a clear setup. Overall the results look promising.

R:
Thank you very much!

A major concern I have however is the absence of comparison or reference to existing methods, as synteny-based scaffolding has been considered by other groups, e.g.:
https://www.jstage.jst.go.jp/article/gi1990/17/2/17_2_152/_pdf/-char/ja (reference 1)
https://bmcgenomics.biomedcentral.com/articles/10.1186/1471-2164-16-S10-S11 (ARt-DeCo)
https://genomebiology.biomedcentral.com/articles/10.1186/s13059-014-0573-1 (ALLMAPS)

R:
As reference 1 shows, the idea of using synteny in genome assembly is not new (we now do cite this reference among others proposed by reviewers#1 and #2). With our manuscript we do not suggest that we have invented synteny-based scaffolding, but want to make a strong example how useful this method is and especially will be in the future of large-scale animal genome projects. We also do not say CSA is the only way

Powered by Editorial Manager® and ProduXion Manager® from Aries Systems Corporation

	to assemble a chromosomal scale genome, but to our knowledge it is currently the computationally most efficient solution for long read data and provides a scheme of how future genome assembly tools could be structured. Regarding benchmarking issues, please, see also answers to reviewer #1.<br><br>ARt-DeCo: as the authors themselves state in their manuscript is lagging behind other methods as it does not use gene orientation.<br>ALLMAPS has been designed to integrate genetic linkage maps with draft genome assemblies. Although synteny data may be somehow fitted into ALLMAPS, other tools seem to be more tailored to do so (RACA or RAGOUT which is used in CSA).<br><br>Also, why do so many of the dot plots show local inversions? Is this a characteristic pattern of the assembler?<br><br>R:<br>We actually rather assume that as a characteristic of chromosome evolution. Many rearrangements between a diverged reference genome and the genome to be assembled may be inversions and sometimes (if the breakpoint region cannot be fully assembled) the true contig placement cannot be determined, in such a situation, the contig might be placed according to the reference genome and results in an inversion. This also explains that we obtain more inversions and translocation errors, if using more diverged reference genomes to assist CSA. A low contig N50 of the primary de novo assembly can have similar effects.<br><br>In response, we discuss this now in L384 - 387:<br><br>"It seems worth to mention that contig N50 length of the primary assembly (CSA step1) is an important factor and should be at least in the mega base range, as low contiguity of the contigs increases the chance of wrongly resolving rearrangements between query and reference genomes."
**Additional Information:**	
**Question**	**Response**
Are you submitting this manuscript to a special series or article collection?	No
**Experimental design and statistics**<br><br>Full details of the experimental design and statistical methods used should be given in the Methods section, as detailed in our Minimum Standards Reporting Checklist. Information essential to interpreting the data presented should be made available in the figure legends.<br><br>Have you included all the information requested in your manuscript?	Yes
**Resources**<br><br>A description of all resources used, including antibodies, cell lines, animals and software tools, with enough information to allow them to be uniquely	Yes

Powered by Editorial Manager® and ProduXion Manager® from Aries Systems Corporation

identified, should be included in the Methods section. Authors are strongly encouraged to cite Research Resource Identifiers (RRIDs) for antibodies, model organisms and tools, where possible.<br><br>Have you included the information requested as detailed in our Minimum Standards Reporting Checklist?	
**Availability of data and materials**<br><br>All datasets and code on which the conclusions of the paper rely must be either included in your submission or deposited in publicly available repositories (where available and ethically appropriate), referencing such data using a unique identifier in the references and in the "Availability of Data and Materials" section of your manuscript.<br><br>Have you have met the above requirement as detailed in our Minimum Standards Reporting Checklist?	No
If not, please give reasons for any omissions below.<br><br>as follow-up to "**Availability of data and materials**<br><br>All datasets and code on which the conclusions of the paper rely must be either included in your submission or deposited in publicly available repositories (where available and ethically appropriate), referencing such data using a unique identifier in the references and in the "Availability of Data and Materials" section of your manuscript.<br><br>Have you have met the above requirement as detailed in our Minimum Standards Reporting Checklist?	Some datasets used for benchmarks are still under submission at NCBI (S.chuatsi and P.fluviatilis long read data and reference genome assemblies)

"	

Powered by Editorial Manager® and ProduXion Manager® from Aries Systems Corporation

Manuscript

Click here to access/download;Manuscript;CSA-manuscript_v7c_GIG_R1_final.docx

CSA: A high-throughput **c**hromosome-**s**cale **a**ssembly pipeline for vertebrate genomes

Heiner Kuhl[1]*, Ling Li [1,2], Sven Wuertz[1], Matthias Stöck[1], Xu-Fang Liang[2] and Christophe Klopp[3]

*Corresponding author

Affiliations

[1] Department of Ecophysiology and Aquaculture, Leibniz-Institute of Freshwater Ecology and Inland Fisheries (IGB), Berlin, Germany.

[2] College of Fisheries, Chinese Perch Research Center, Huazhong Agricultural University; Innovation Base for Chinese Perch Breeding, Key Lab of Freshwater Animal Breeding, Ministry of Agriculture, Wuhan, China.

[3] Sigenae, Mathématiques et Informatique Appliquées de Toulouse, INRA, Castanet Tolosan, France.

E-mail addresses

Heiner Kuhl:	kuhl@igb-berlin.de
Ling Li:	ling.li@igb-berlin.de
Sven Würtz:	wuertz@igb-berlin.de
Matthias Stöck:	matthias.stoeck@igb-berlin.de
Xu-Fang Liang:	xfliang@mail.hzau.edu.cn
Christophe Klopp:	christophe.klopp@inrae.fr

Abstract

Background: Easy-to-use and fast bioinformatics pipelines for long-read assembly that go beyond the contig-level to generate high-quality chromosome-scale genomes from raw data remain scarce.

Results: Chromosome Scale Assembler (CSA) is a novel computationally highly efficient bioinformatics pipeline that fills this gap. CSA integrates information from scaffolded assemblies (e.g. Hi-C or 10X Genomics) or even from diverged reference genomes into the assembly process. As CSA performs automated assembly of chromosome-sized scaffolds, we benchmark its performance against state-of-the art reference genomes that have been built in a laborious fashion using multiple separate assembly tools and manual curation. CSA increases the contig length using scaffolding, local re-assembly and gap-closing. On certain datasets, initial contig N50 may be increased up to 4.5-fold. For smaller vertebrate genomes, chromosome-scale assemblies can be achieved within 12 h using low cost, high-end desktop computers. Mammalian genomes can be processed within 16 h on compute-servers. Using diverged reference genomes for fish, birds and mammals, we demonstrate that CSA calculates chromosome-scale assemblies from long-read data and genome comparisons alone. Even contig-level draft assemblies of diverged genomes are helpful for reconstructing chromosome-scale sequences. CSA is also capable of assembling ultra-long reads.

Conclusions: CSA can speed-up and simplify chromosome-level assembly and significantly lower costs of large-scale family-level vertebrate genome projects.

Keywords

Genome assembly, genome scaffolding, long-read, comparative genomics, genome evolution, chromosomes, vertebrates

Findings

Background

Whole genome shotgun (WGS) assembly in vertebrates – state of the art

WGS assembly of large vertebrate genomes has been an important topic of bioinformatic research over the last two decades, but obtaining completely assembled chromosomes through a single bioinformatics tool has not yet been achieved for large vertebrate genomes. Despite the ongoing replacement of short- by long-read sequencing in *de novo* genome projects, chromosome-level assemblies of vertebrates still require great bioinformatics expertise, especially in projects, where cutting-edge genome maps or scaffolding data are not available.

Today, most vertebrate genomes can be assembled using noisy long reads[1-3] and the results - in terms of assembly contiguity, measured as contig N50 - can outperform results obtained by short-read sequencing by a factor greater than 100. Contig N50 of today's noisy long-read assemblies reaches lengths similar to scaffold N50 of high-quality short-read genome assemblies some years ago. Still, current assembly tools can profit from their ancestors[4-9]. So far most of them produce only contigs[10-14] and do not incorporate additional information to order these contigs into scaffolds, which would enable further gap-closing and lead to chromosomal-level assemblies.

Chromosomal-level genome assembly as the final goal of a genome project still requires additional scaffolding or mapping data (Hi-C[15-17] or optical mapping[18], high density genetic linkage map[19]), resulting in additional efforts that may add significant human, time and financial resources to sequencing projects. For many, especially rare species, DNA-resources for *de novo* genome sequencing come from archival tissues (e.g. frozen or ethanol-fixed or preserved in other storage media), preventing the application of Hi-C, which requires living cells, and thus mapping panels can hardly be established. In such and similar cases, synteny and gene order analysis between evolutionary related genomes may be the only option to improve the genome assembly process.

Synteny as a common feature of vertebrate genomes

All vertebrates, with currently ca. 71,000 scientifically described species (August 2019), experienced two ancestral whole genome duplications (WGDs), leading to ca. 38,000 extant tetrapods, while most of the ca. 33,000 teleosts have gone through a 3rd WGD[20, 21]. Beyond different ancestral WGD-"substrates" that influenced the evolution of deletions, silencing and/or pseudogenization, sub- and neofunctionalization, genome size in vertebrates differs strongly (typical size range 0.4–4 Gbp; in this regard the huge genome sizes of amphibians pose strong exceptions: c-value (haploid genome size) 3.3–57 pg (http://genomesize.com)). While neither associated with morphological complexity nor gene numbers, genome size differences are caused by quantities of various repetitive elements and other non-coding DNA, comprising up to 98% of vertebrate genomes[22].

Despite these WGD and size differences, "structural conservation" of vertebrate genomes as inferred from the distribution and positioning of genes on chromosomes, known as synteny, is a major feature of their evolution[23] with a pattern of conserved syntenic associations dating back 360 My[24], and even 600 My in other metazoans[25, 26]. Locations and order of genes (also referred to as "blocs") in genomes depend on phylogenetic relatedness and on the "substrates" evolved after the ancient WGDs. Despite synteny, the various classes of vertebrates show different speed of chromosomal and sequence, and thus genome evolution.

Teleost fishes exhibit accelerated evolutionary rate of protein-coding and other sequences, a higher rate of intron turnover, loss of many potential *cis*-regulatory elements and shorter conserved syntenic blocks[27, 28]. Due to their mostly enormous genome size with huge repetitive fractions, only 26 amphibian genomes have been sequenced and few reached chromosomal scale quality, with deep divergences (often >100 My) between systematic amphibian families posing additional challenges. Nevertheless, ortholog genes that exhibit distinct order in bird chromosomes are also discretely ordered in the single well-assembled urodelan (*Ambystoma mexicanum*)[29, 30] and the few anuran genomes (*Xenopus tropicalis*, *Nanorana parkeri)*[31, 32], suggesting that ancestral chromosome segments and structures also remained conserved during amphibian phylogenesis[23].

Conservation of chromosomes, syntenic with avian autosomes, has been demonstrated in squamate

reptiles[33], in which numerous microchromosomes pose special challenges for genomics[34]. Whole-genome comparisons among birds and mammals point to genomic regions where the orthologous gene order has been maintained for tens of millions of years[35, 36].

In summary, despite specific genomic properties of various vertebrate classes, synteny and conserved gene order present common and long-known inherent features of vertebrate genomes[37] that deserve to be better considered during genome assembly and that can be exploited by current bioinformatics.

Exploiting synteny information for new approaches in vertebrate genomics

Indeed, evolutionary relationships such as highly conserved chromosome structure (synteny and gene order) in related vertebrate species[38], in some taxa even between several taxonomic-levels[39], can enable low-cost approximations of chromosomal-scale assembly by comparative genomics[40-43]. An example for a successful short-read application is the genome assembly pipeline IMAP[44]. Of course, such an approach requires the existence of at least one suitable high-quality reference genome, which has become a dwindling problem as for each vertebrate order at least one "platinum grade" reference genome will soon be created in Phase I of the international Vertebrate Genome Project (VGP an offspring of the genome10K project)[45] and more will follow in the course of other large-scale genomics projects, like the Earth BioGenome Project (EBP)[46].

Here, we present a novel bioinformatics pipeline, which we call "Chromosomal Scale Assembler" (CSA). CSA overcomes limitations of current long-read assemblers by integrating comparisons between diverged reference genomes and/or scaffolds, derived from optical mapping, Hi-C or 10X Genomics into the *de novo* assembly process. CSA runs computationally highly efficient tools for long-read genome assembly, whole genome alignments and reference-assisted chromosomal assembly in an iterative fashion. We show that CSA is able to produce chromosomal-level assemblies for smaller vertebrate genomes (fishes, birds) within 12 h on low cost computing equipment (1000 – 2000 $, Intel i7, 128 GB RAM), using just long-read data and a diverged reference genome (div. time ~65 Mya) as input. Larger mammalian genomes, such as human, can be assembled within 16 h on

server equipment (Intel Xeon, 1 TB RAM). Depending on the type and coverage of the input data, CSA is able to improve contig N50 length up to 4.5-fold from initial to final contig assembly.

Results and Discussion

Implementation of the CSA pipeline

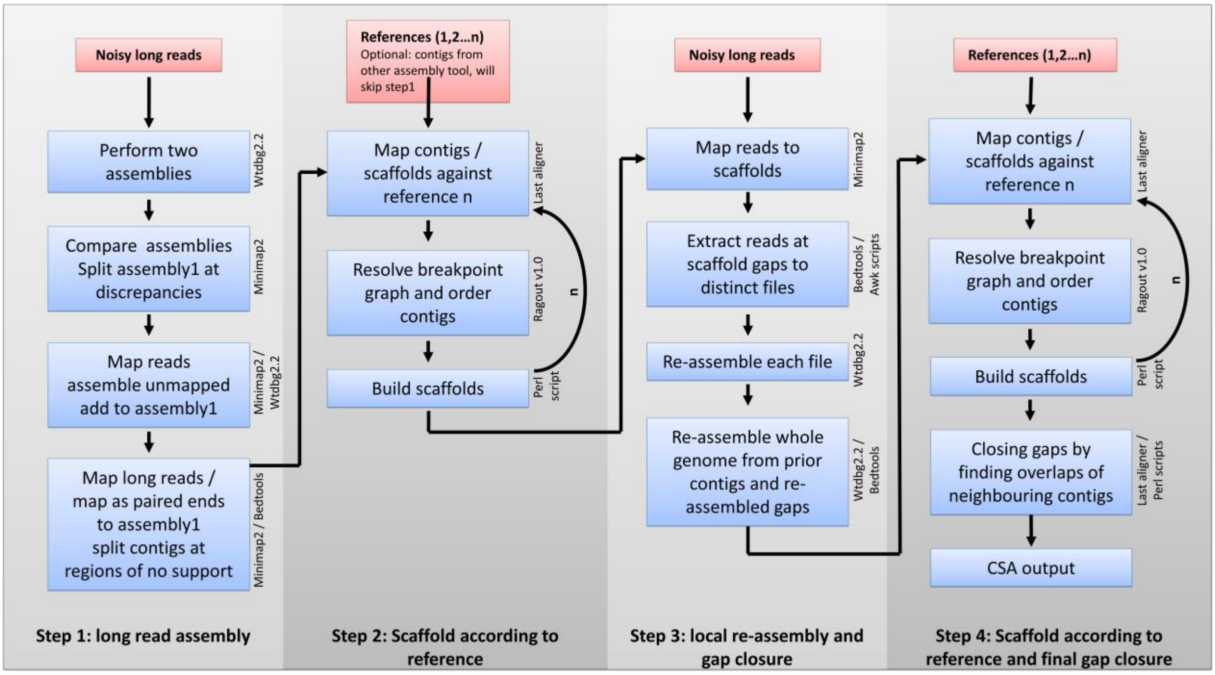

Figure 1: Flowchart of the four step CSA pipeline.

The first step of CSA (Figure 1) is a *de novo* assembly of noisy long-read data (either Pacific Bioscience or Oxford Nanopore data). It employs the WTDBG2 (version 2.2 11 Dec. 2018) assembler as it is among the most computationally efficient *de novo* genome assemblers to date[14]. CSA runs two WTDBG assemblies with slightly varying parameters and splits the contigs at discrepancies between both assemblies to get rid of rarely occurring misassemblies. Additionally, we re-assemble long reads that can only be partially mapped to the WTDBG2 assembly (< 10 % of read-length); this step may recover up to 1-2 % of genomic sequence (large contigs) that is missing in current WTDBG2 primary assemblies. A final assembly curation is performed by re-mapping (MINIMAP2)[47] long reads and paired ends of long reads (500 bp from each end) to the assembly and split contigs at regions of zero

coverage. If needed, the WTDBG2 assembly can be omitted and a contig file from another genome assembly tool can be used by CSA; thus, CSA can also be used to update existing assemblies.

In the second step, the resulting curated contigs are mapped by LAST aligner[48] to one or more references. These references may be scaffolds from the same species that have been built using various methods (e.g. 10X Genomics, optical mapping or Hi-C). An outstanding feature of CSA is that even diverged reference and draft genomes are a suitable input. The LAST alignments are used by RAGOUT[49] to order the curated contigs (from step 1) into scaffolds, which then already may reach chromosomal size.

During step three all noisy long reads are mapped to the scaffolds by MINIMAP2. Reads that map in 20 kbp-windows around scaffold gaps or contig ends are extracted into distinct fasta files. These files are submitted to the WTDBG2 assembler and are locally re-assembled in parallel. The resulting local re-assemblies for each gap/contig end are then assembled with the primary WTDBG2 contigs that have been split into overlapping pseudo-reads to meet the read length limits of WTDBG2 (256 kb in version 2.2, version 2.4 has no limits, but showed lower performance in tests). As WTDBG2 now assembles pre-assembled reads with higher accuracy (consensus accuracy 98-99%), more stringent parameters are set. This iterative assembly step can typically double N50 contig sizes as shown in different tests hereafter. Alignment of the improved contigs to the prior scaffolds is used to remove few intra- and inter-scaffold misassemblies.

Step four, again, maps the improved contigs against the references used in step2, by LAST aligner and runs RAGOUT to order the contigs into scaffolds. Finally, some gaps with overlapping neighbouring contig-ends are identified by LAST and closed.

The tools selected to build the CSA pipeline have been mainly chosen based on performance and sensitivity when using diverged reference genomes. Relatively simple procedures split primary *de novo* assemblies at lowly supported regions (e.g. non-continuously covered regions in comparisons of two *de novo* assemblies or regions of low read coverage), which is relatively fast, even when dealing with very large genomes. For assembly re-conciliation, more sophisticated tools have been developed, for instance SMSC[50] or BIGMAC[51], but these have been rarely tested on large

genomes and, according to published benchmarks on small-sized genomes (bacteria, yeast), these modules might take longer than the entire CSA pipeline. Nevertheless, improved faster detection of misassemblies by read re-mapping could further improve assembly quality in future versions of CSA.

Another well-tuned example seems our choice of RAGOUT, which can be easily adapted to the maf format output of the fast and sensitive LAST aligner, while similar tools like MeDuSa[52] and RACA[40] use computationally more expensive (LASTZ in RACA) or less sensitive aligners that do not work well on diverged reference genomes (MUMMER[53] in MeDuSa). RAGOUT1[49] was preferred over RAGOUT2[41], because it resulted in slightly better chromosome assemblies. RAGOUT can also close gaps, but this feature was designed for short, nearly error-free overlaps of contigs from short-read assemblies and is not working with long-read assemblies. Thus, we implemented own solutions for local gap reassembly and final contig stitching. In principle, gap closing could also be done by tools such as PBJelly[54] or LR_gapcloser[55], but we found that some closable gaps remain unclosed by these tools, probably due to overlapping repeat sequences at some contig ends which do occur even in long read assemblies.

The current version of CSA does not include methods for consensus polishing, yet, one iteration of consensus polishing using long-reads and two iterations using short-reads should be applied prior to sequence annotation efforts.

In the following, we tested CSA on different scenarios and benchmark its performance. CSA automatically chains many steps that traditionally require different software tools and laborious manual curation, for which similar pipelines are currently only available for short-read assembly (IMAP[44]). Therefore, we do not compare CSA-results with known contig-level genome assembly tools or other software packages solving only parts of chromosomal assemblies, but by re-assembling the currently best chromosomal-scale reference genomes in different vertebrate species.

Benchmark scenario 1: Updating existing fish, bird and mammal assemblies, using a prior assembly version as reference

The current CSA pipeline was tested using SMRT (single molecule real time; Pacific Biosciences) long-read sequencing data for representative species of three different vertebrate clades, namely mammalia (*Homo sapiens* = Hs), aves (*Taeniopygia guttata* = Tg) and teleostei (*Siniperca chuatsi* = Sc). Our first tests used high-quality genomes of the same species from which the long-read input data was derived to assist the assembly. These tests show what we can expect from CSA in a best-case scenario. In a real-world scenario, where no known reference of the same species is available, this approach would be comparable to using CSA and scaffolding the CSA step 1 assembly by Hi-C data and then continuing with assembly improvements (CSA step 2-4). The detailed results of these benchmarks are shown in suppl. table 1.

In terms of chromosomal assembly completeness, we measured, how much of the consensus sequence is contained in the top n largest scaffolds, where n is the haploid chromosome number. All CSA assemblies placed more than 94% of the consensus into the top n scaffolds (Hs = 97.5%; Tg = 94.2%; Sc = 99.3%). The contig N50 length was 25.9 Mbp, 27.7 Mbp and 16.5 Mbp for Hs, Tg and Sc, respectively. These values outperform the current reference contig N50 for Tg (VGP assembly) and Sc (own results), which are based on the same long-read input data but included different genome maps and curation steps to improve the assembly. For Hs we compared contig N50 to the so far best assembly from Pacbio (Acc: GCA_003634875.1) data and found that CSA produced similar values, although we used an older dataset (P4C6 chemistry from RSII sequencer) for our tests. For Tg, we could improve contig N50 by 2.3-fold over the VGP assembly. The contig N50 of Sc improved 1.35-fold over our sinChu7 assembly.

Finally, we compared CSA assemblies versus the references to visually inspect assembly errors by dot plots (Figure 2 left) and counted larger scale synteny (gene order) breaks (rearranged genomic blocks >300 kbp) by custom scripts. The CSA assemblies exhibited only few structural misassemblies (**f** = interchr. Fusion/fission, **t** = intrachr. translocation, **i** = inversion: **Hs:** f: 0; t: 6; i: 2 / **Tg:** f: 0; t: 1; i: 4

/ **Sc:** f: 0; t: 1; i: 5). For the teleost assembly, CSA even polished two misassemblies in the current reference genome (1 fusion and one 1 inversion).

These results show that under our best case scenario the pipeline performed very well and CSA appears as a valuable tool to improve existing reference genomes by complete re-assembly as soon as improved sequence data is available.

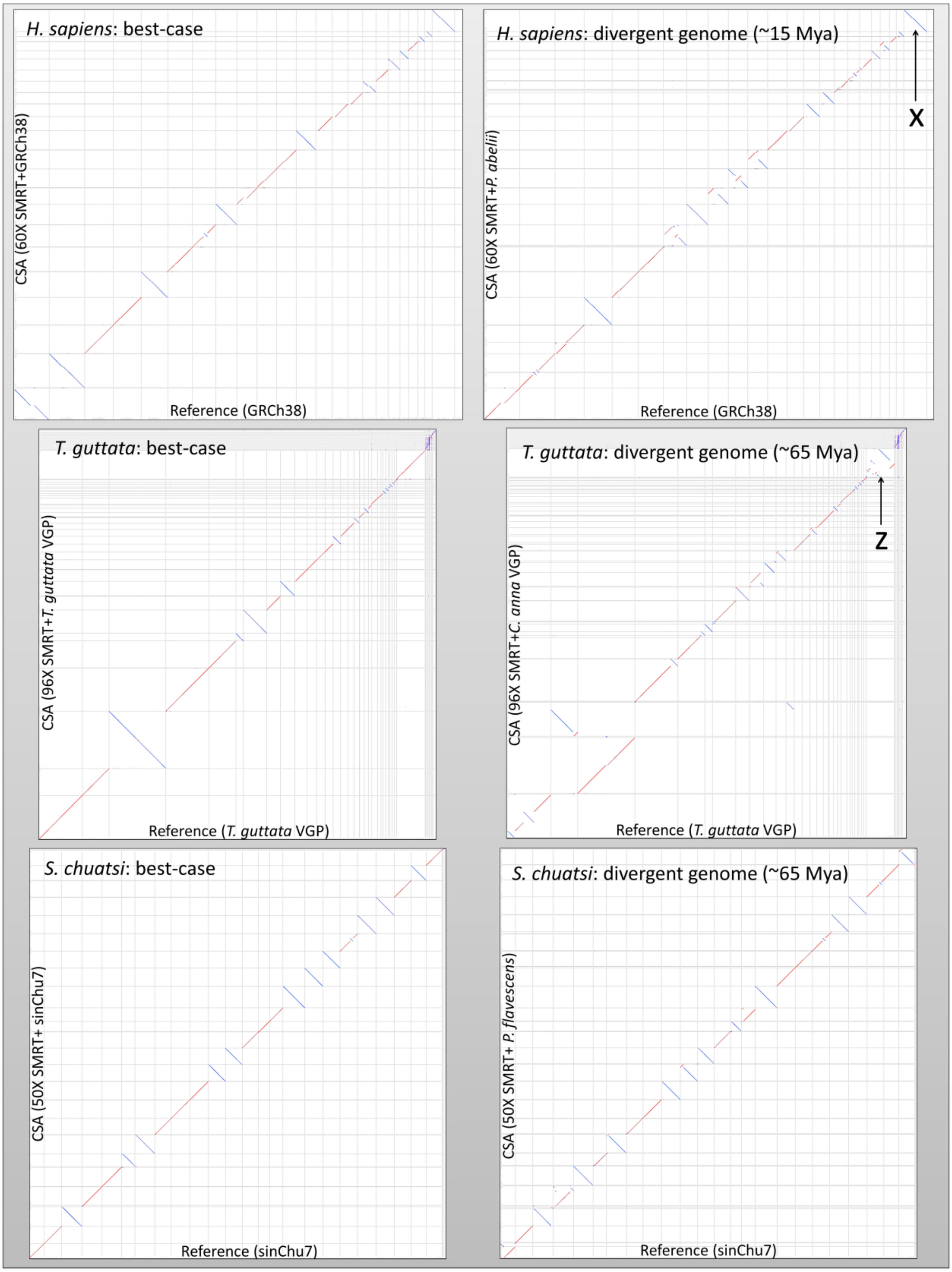

Figure 2: Dot plots of CSA results against reference genomes under best-case (left) and diverged reference scenarios (right) for mammal, bird and fish genomes. Thin vertical lines separate chromosomes of the reference assembly; thin horizontal lines separate CSA-scaffolds. Red and blue colours depict forward or reverse orientation of the alignments. Lines that are not placed on diagonals or sub-diagonals indicate rearrangements between reference and CSA-assemblies. Note, that a single blue line match per reference chromosome does not mean a large inversion is present, but that CSA just outputted the corresponding chromosomal scaffold as reverse complement orientation. X chromosomes in *H. sapiens* and Z chromosomes *T. guttata* are marked in the plots. In *T. guttata*, the Z chromosome shows a higher number of rearrangements than the autosomes.

Benchmark scenario 2: CSA using divergent genomes as reference, allows chromosomal scale assemblies from long reads only

Although this approach can be limited by complex evolutionary scenarios involving major re-arrangements of genomes, in principle, the mapping steps in CSA have been designed to allow for incorporation of highly diverged genomes as references. Nowadays (and in the future even more) one will find suitable, perhaps distantly related reference genomes for most vertebrate species in databases and this gives us the opportunity to obtain high-quality chromosome-scale assemblies from long-read data alone – potentially even without having other mapping data at hand (e.g. Hi-C, optical maps, linkage maps). We tested CSA on the long-read data from above using high-quality reference genomes of species that diverged between 10-240 Mya as references. Representative CSA-assemblies, using diverged references, are shown in table 1 and compared to state-of-the-art reference assemblies, the detailed results for all benchmarks are shown in suppl. table 2.

Table 1: CSA enables chromosomal-scale genome assemblies of mammal, bird and fish by using just long-read data and a diverged reference genome. In most cases, CSA improved the contig N50 length over current state-of-the-art reference genomes. Completeness of chromosomal assembly was in the range of 92.1% - 94.7%, only slightly below what can be obtained by using Hi-C data (97.8% – 99.0%). Structural discrepancies (>300 kbp) with the reference genomes were low, especially for the contig-level and, in case of the human genome, even lower than a comparable assembly, which used Hi-C mapping (GCA_003634875.1). ULR = ultra-long read; SMRT = single molecule real-time sequencing; f = interchr. fusion/fission; t = intrachr. translocation; i = inversion.

organism (assembly)	assembly strategy	total assembled bp length without gaps [bp]	contig N50 length	% bp placed in top n scaffolds	structural discrepancies in scaffolds vs. reference	structural discrepancies in contigs vs. reference	assembly time frame
Homo sapiens (GRCh38) **REFERENCE**	clone based, extensive manual curation	2.937.639.113	50.761.348	98,64% (n=23)	-	-	years
Homo sapiens (GCA_003634875.1)	SMRT + Hi-C	2.893.114.812	26.292.878	97,82% (n=23)	f:25; t:58; i:14	f:5; t:0; i:4	days/weeks
Homo sapiens **(CSA)**	SMRT + diverged genome 10-20Mya *(P.abelii)*	2.849.099.702	29.334.513	93,80% (n=23)	f:0; t:58; i:8	f:0; t:4; i:6	hours/days
Homo sapiens **(CSA)** (see benchmark scenario 6)	ONT ULRs + diverged genome 10-20Mya *(P.abelii)*	2.884.341.430	45.943.940	92,90% (n=23)	f:2; t:53; i:8	f:1; t:5; i:6	hours/days
Taeniopygia guttata (VGP) **REFERENCE**	SMRT + Optical map + Hi-C + curation	1.054.772.052	11.998.827	98,98% (n=40)	-	-	days/weeks
Taeniopygia guttata **(CSA)**	SMRT + diverged genome 65Mya *(C.anas)*	1.096.370.479	18.882.724	92,13% (n=40)	f:1; t:33; i:20	f:0; t:1; i:6	hours
Siniperca chuatsi (sinChu7) **REFERENCE**	SMRT + high density linkage map + curation	753.983.610	12.191.788	96,68% (n=24)	-	-	days/weeks
Siniperca chuatsi **(CSA)**	SMRT + diverged genome 65Mya *(P. flavescens)*	721.014.191	16.688.192	94,73% (n=24)	f:1; t:13; i:16	f:0; t:0; i:5	hours
Perca fluviatilis (PFLU1.1) **REFERENCE**	ONT + Hi-C + curation	950.435.818	2.593.362	99,00% (n=24)	-	-	days/weeks
Perca fluviatilis **(CSA)**	ONT + diverged genomes 10-20Mya and 65Mya *(P. flavescens + S. chuatsi)*	928.809.152	7.745.610	93,79% (n=24)	f:0; t:19; i:15	f:0; t:0; i:3	hours

Overall, the fraction of consensus sequences assigned to the top n scaffolds was slightly lower than under the best-case scenario, but it was well above 90% for the less diverged references. The loss of placed sequence typically occurs in the subtelomeric regions that diverge faster than the other chromosomal regions. In most cases, using more diverged reference genomes, CSA still allowed to place more than 92% of the assembly in the top n scaffolds.

Improvements of contig N50 were still observed at a similar scale as in the best-case scenario and introduced assembly errors due to divergent reference genomes were low on the contig-level (suppl. table 2; row: "Errors ctg"). Our main focus of this benchmark was to analyse large-scale misassemblies that are introduced by using diverged genomes as references in the chromosomal scaffold assembly (Figure 2 right, additional plots suppl. figure 1 and 2) and how these develop with increasing divergence time. As expected, here we saw clear differences between mammals, birds and teleosts.

Chromosomal gene order is highly conserved in birds[39] and among vertebrates, bird genomes have the lowest fraction of repetitive sequences (< 20%)[56]. This possibly explains why CSA works very well for most of autosomes when using diverged bird genomes (up to 90 Mya) as reference. Nevertheless, here we found few chromosomal fusion errors that were related to known differences in bird karyotypes (e.g. fusion/fission of chr1/chr1A; chr4/chr4A etc.) and a clear enrichment of inversion and translocation errors on the Z-chromosome (*Gallus gallus*: 32% and *Calypte anna*: 35% of t and i errors on Z), possibly a result of fast evolution of the Z/W sex chromosomes, which has been decribed earlier[57]. Error profiles were f: 1; t: 33; i: 20 and f: 2; t: 26; i: 20 when using *C. anna* (~65 Mya) and *G. gallus* (~90 Mya) as reference respectively. Finally, CSA still worked reasonably well using the scaffold-level *Alligator mississipiensis* draft genome as reference which has diverged about 240 Mya (f: 2; t: 22; i: 18).

In mammals, assembly errors were distributed more evenly over autosomes and for the X-chromosome we did not find an enrichment of errors, like in the bird Z chromosome. The three-times larger and more repetitive (> 30%) mammal genomes[56] were more prone to misassemblies with

increasing divergence time of the reference than bird genomes. Still, CSA results were good for references that diverged 10-20 Mya ago (in our example *Pongo abelii:* f: 0; t: 58; i: 8).

In teleosts, we did not observe chromosome specific assembly issues and the increase of misassemblies with divergence of the reference was not as harsh as in mammals, possibly due to the more compact genomes of most teleosts. Thus, CSA performed well when using fish reference genomes with divergence times smaller than 65 Mya (here *Perca flavescens*: f: 1; t: 13; i: 16).

Our results show that long-read data and well-chosen, order-level state-of-the-art reference genomes enable CSA to calculate high-quality assemblies for most chromosomes, but in some cases clade-specific problems have to be resolved by manual curation. As a rule of thumb, if choosing from reference genomes that have similar divergence times to support assembly of a new genome, those with the same haploid chromosome number and slowest evolution (as depicted by small branch length in phylogenetic trees or a high fraction of alignable sequence between assembled and reference genome) should be preferred. Nevertheless, considering the contig-level, the usage of distant genomes as references in CSA is quite safe and produces only few errors (supplementary table 2, compare “Errors scf” against “Errors ctg”), but still is able to highly improve contig N50.

Benchmark scenario 3: CSA on a fish genome, integrating Oxford Nanopore reads, 10X Genomics scaffolds and diverged reference genomes

The previous benchmarks did use SMRT long-read data and a single reference genome. In the following, we ran CSA using long reads generated by Oxford Nanopore (ONT) sequencing from genomic DNA of *Perca fluviatilis* and supported the assembly by a 10X Genomics assembly of the same species and two diverged reference genomes.

A high-quality chromosomal-scale genome assembly of *P. fluviatilis* assembled from the same ONT long reads and Hi-C sequencing has recently become available (BioProject Acc: PRJNA549142). The high-quality draft genome assembled from 10X Genomics sequence data has been published earlier (68X Illumina short-read coverage, N50 contig / scaffold length 18.3 kbp / 6.3 Mbp according to [58]). As a close relative (genus-level) of *P. fluviatilis*, a chromosomal-scale reference genome for *P.*

flavescens is available[59]; both *Perca* diverged about 8.2-17.5 Mya. As a more distantly related reference (div. time ~65 Mya), we used the chromosomal-scale *Siniperca chuatsi* genome (BioProject Acc: PRJNA513951).

To test, whether CSA could reach a similar assembly quality as for the *P. fluviatilis* reference genome, we ran CSA on *P. fluviatilis* ONT long reads, supported by 10X Genomics scaffolds, *P. flavescens* and *S. chuatsi* genomes (see suppl. table 3).

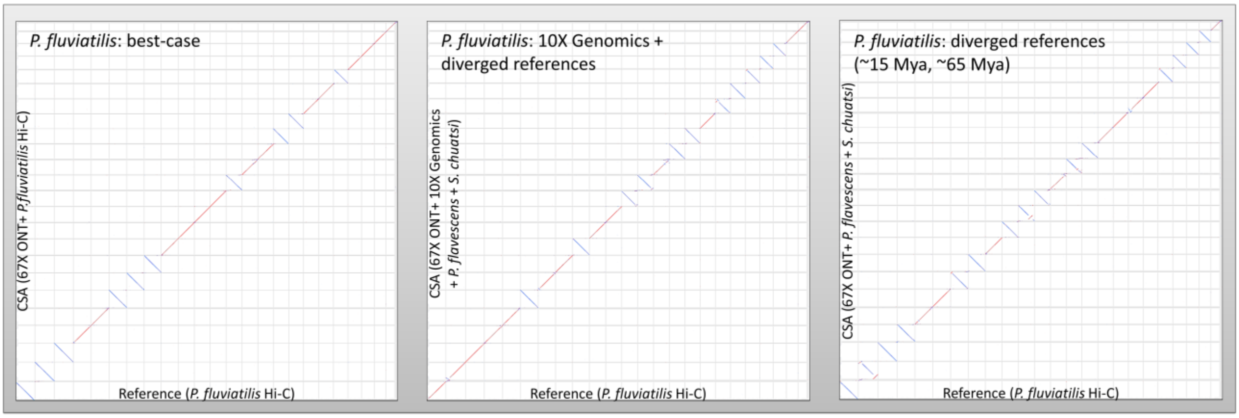

Figure 3: Dot plots of CSA results against reference genome for assembly of *Perca fluviatilis* Oxford Nanopore data, sequentially supported by 10X Genomics and two diverged references genomes. For explanation of the dot plot properties, see Figure 2.

CSA managed to place 94.3% of the assembled sequence into 24 large scaffolds, which corresponded to the reference chromosomes. Only few differences (f: 0; t: 12; i: 13) in chromosomal structure were apparent in the dot plot (Figure 3). CSA increased the contig N50 nearly 3.1-fold compared to the reference genome. The total time to complete the chromosomal assembly was 5h:30min, when using 80 CPU threads on a HPC server or 12 h on a high end desktop computer (12 CPU threads, 128 GB RAM). We polished the CSA assembly using MEDAKA (by ONT: https://github.com/nanoporetech/medaka) and PILON[60] and performed BUSCO[61], which confirmed that the assembly was highly complete on the gene level (Actinopterygii dataset, complete genes: 95.9%, fragmented genes: 2.1%, missing genes: 2.0%, number of tested genes: 4584).

Next, we performed the same runs and omitted the 10X Genomics scaffold data. This only resulted in a slight loss of sequences placed in chromosomes (now 93.8%; loss 0.5%) and a few more intrachr. translocations and inversions (f: 0; t: 19; i: 15).

Finally, using only the most diverged reference still resulted in 86.3% of sequence placed in the top 24 scaffolds, but more intra-chromosomal translocations (f: 0; t: 57; i: 16). Yet, improvement of contig N50 was still 2.7-fold and structural errors in contigs were low (f: 0; t: 0; i: 4).

Thus, CSA was able to compute chromosome-scale assemblies by sequentially using species-, genus- and order-level references together with ONT long-read data. The species-level reference (10X Genomics, Supernova assembly) was only slightly contributing to the final assembly due to its lower N50 scaffold length of 6.3 Mbp. We have observed that ONT long-read datasets of comparable N50 read length and coverage produce less contiguous assemblies than SMRT datasets, possibly due to coverage bias of genomic sequences that interfere with ONT sequencing. According to our results the two gap closure steps performed by CSA were highly efficient to improve contig N50 in such a situation.

Benchmark scenario 4: CSA using draft assemblies as reference; contig-level assemblies of diverged species may be highly complementary

Under scenario 2 we already found that draft assemblies of other species could be used to improve genome assemblies (*T. guttata* / *A. mississipiensis* results). So we asked the question, if a diverged, low N50 contig-level assembly could still support CSA to result in improved assemblies.

Thus, we assembled the *S. chuatsi* genome using *P. fluviatilis* contigs (from scenario 3 CSA step 1: N50 = 2.8Mbp) as reference. Although the reference contig N50 was relatively low, it was improving the *S. chuatsi* assembly significantly (suppl. table 2 last column). The *S. chuatsi* assembly continuity doubled from a N50 11.6 Mbp (primary contigs) to 23.4 Mbp (final scaffolds). The top 24 scaffolds consisted of 77.26% and the top 48 scaffolds consisted of 89.9% of the assembled sequence, thus chromosomal assembly was less complete, but possibly most chromosome arms were well assembled (Figure 4). Interestingly, the improvement of contig N50 (1.37-fold) due to gap closure was similar to the tests performed with high-quality reference genomes in scenario2 and the number of assembly errors was low (scaffolds: f: 0; t: 2; i: 9 / contigs: f: 0; t: 0; i: 5).

Thus, CSA is able to use even low continuous contig assemblies of diverged species to improve genome assemblies. This opens up new strategies in projects, where many species of a certain clade are sequenced and might complement the assemblies of each other already at draft state.

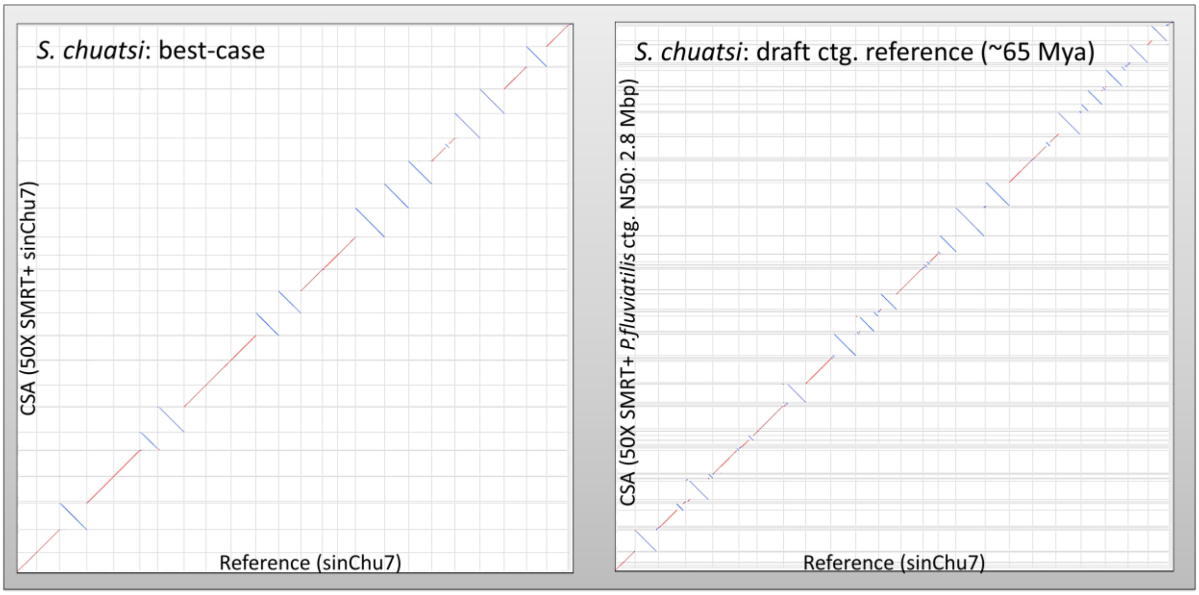

Figure 4: Dot plots of CSA results against reference genome for assembly of *Siniperca chuatsi* supported by contigs of a diverged draft genome assembly in comparison to the best case CSA assembly. For explanation of the dot plot properties, see Figure 2.

Benchmark scenario 5: Benchmarking influence of long-read sequencing coverage

On primary assemblies of lower contig N50 length, CSA can play its strength in gap closure. As this was already observed in scenario 3, we now asked the question how long-read sequencing coverage does influence the results of CSA assembly. We randomly subsampled reads from the *H. sapiens* 60x SMRT sequencing dataset, to obtain subsets of 15x, 20x, 30x and 40x sequencing coverage. We observed only slight changes of the final results for 60x, 40x and 30x sequencing coverage. Although contig N50 of the primary assembly started to drop below 30x, the CSA gap closures in step 3 and 4 still enabled a final contig N50, similar to what was obtained from the 40x and 60x datasets (suppl. table 4). The 20x and 15x data had significantly lower contig N50, here the improvement by the CSA gap closure was clearly the highest (3.8-fold for 20x and 4.5-fold for 15x), but assembly errors (especially fusion errors) started to increase (Figure 5). Similar results were observed, if using the diverged *P. abelii* genome as reference. It seems worth to mention that contig N50 length of the primary assembly (CSA step1) is an important factor and should be at least in the mega base range,

as low contiguity of the contigs increases the chance of wrongly resolving rearrangements between query and reference genomes.

Thus, when running CSA, 30x sequencing coverage is sufficient and even lower coverage may lead to respectable results. Particularly, low coverage assemblies take profit from gap closure steps, and CSA can improve contig N50s by several hundred percent.

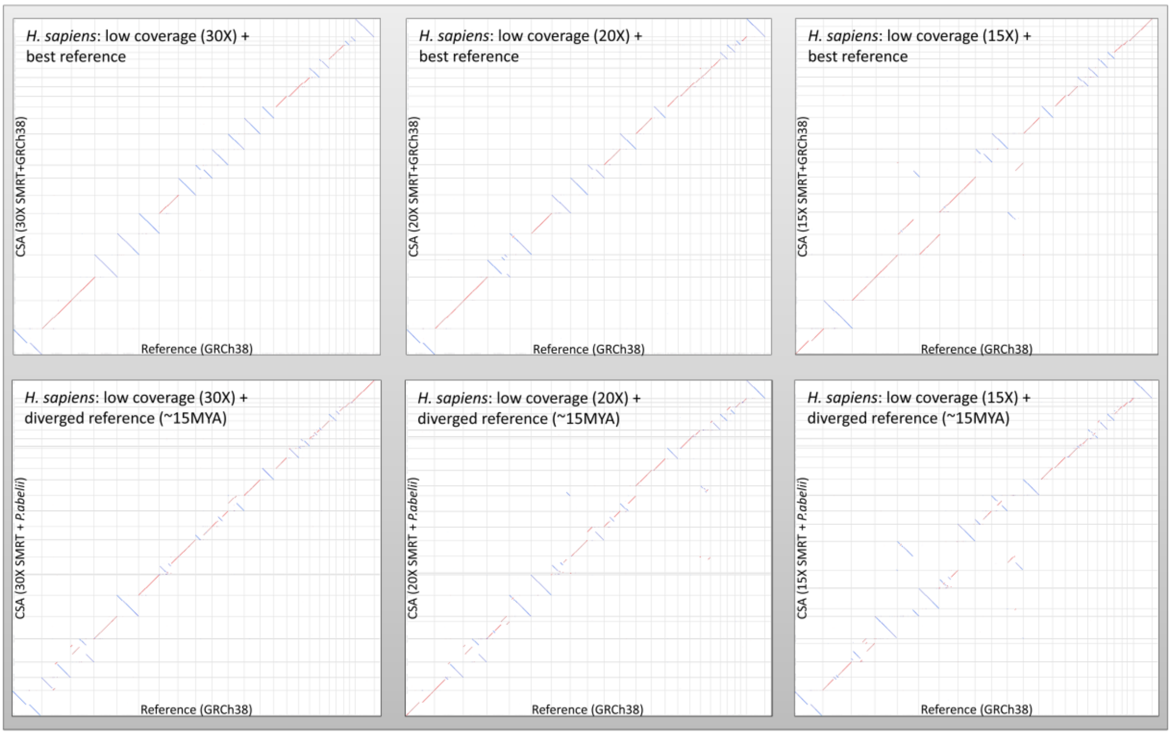

Figure 5: Dot plots of CSA results against reference genome for reduced coverage data (*Homo sapiens*), using either the best reference or a diverged reference to support CSA. For explanation of the dot plot properties, see Figure 2.

Benchmark scenario 6: Ultra long-read assembly

Ultra-long reads (ULR, N50 read length > 50 kbp) are currently gaining importance in the sequencing community and will possibly be available to many researchers soon. CSA default parameters have been optimized for current long-read data (N50 read length < 30 kbp). It has been reported recently that WTDBG2 performs relatively poor on ULR data compared to the SHASTA assembler, which was designed for ULR assembly[62]. We found that optimization of some parameters of WTDBG2 did overcome these issues (increasing minimum read length cut-off to about N50 read length, while maintaining sequence coverage >25X and increasing minimum overlap cut-off to about 30% of the N50 read length). We found that CSA was running more slowly due to its several read re-mapping

steps, which are computationally less efficient when using ULR data. Still, the assembly finished within 24 h on our compute-server. Our CSA ULR assembly (best-case, using GRCh38 as reference; details: suppl. table 5) did compete well in terms of contig N50 (48.4 Mbp vs. 46.0 Mbp) with the SHASTA assembler, producing significantly less contigs (1,526 vs. 1,925) and more complete total consensus length (2.9 Mbp vs. 2.8 Mbp, before sequence polishing). The number of structural misassemblies in CSA scaffolds, if compared against GRCh38 human reference chromosomes, was similar to using SMRT reads in scenario 1 (f:0; t:9; i:2, dot plot in suppl. figure 1). On the contig-level, we could compare CSA and SHASTA assemblies, which were both nearly free of large structural errors (CSA: f:0; t:0; i:2 / SHASTA: f:0; t:0; i:0). This picture only slightly changed, if we used a diverged reference during the CSA assembly (table 1, suppl. Table 5 and suppl. Figure 3). In this case, contig N50 of SHASTA and CSA were similar (both about 46.0 Mbp) and CSA error-rates were slightly higher (scaffold-level: f2; t:53; i:8 / contig-level: f:1; t:5; i:6).

Conclusions

Considering the scenarios tested, we have shown that CSA is a reliable tool that goes far beyond the contig-level assembly of long reads and enables automated chromosome-scale assemblies. Nevertheless, well-known assembly issues, like genomes exhibiting high heterozygosity, higher ploidies or extreme repeat content and genome size may still result in assemblies of lower contiguity. For example, the few available high-quality amphibian genomes, are currently posing challenges to CSA for this vertebrate class, as long as no high-quality Hi-C scaffolds from at least a relatively closely related species are available to support the assembly (model genomes from each systematic amphibian family would pose a great progress).

Yet, considering mammals, birds, fishes and possibly reptiles, CSA allows for lower sequencing coverage in genome projects and reduces the need for computational resources. Thus, CSA can contribute to save significant human, time and financial resources and thus cost-reduction in small- and large-scale genome projects. Furthermore, CSA enables beginners to genome assembly to perform chromosomal-level assemblies, even on datasets that would be considered suboptimal,

when using other assembly tools. We are confident that CSA presents another important step towards the democratization of genome sequencing and assembly.

Acknowledgements

We thank Yann Guiguen for granting early access to *P. fluviatilis* long-read data. We also thank the authors of various tools that make up the CSA pipeline, especially: Jue Ruan (WTDBG2), Heng Li (MINIMAP2), Mikhail Kolmogorov (RAGOUT) and Martin C. Frith (LAST). We thank the Vertebrate Genomes Project (VGP) and the Telomere-to-Telomere (T2T) consortium for making reference genome assemblies and read-data publicly available for benchmarking purposes.

Availability of supporting data

Code snapshots are available in the manuscript supplements. Test data is publicly available at NCBI.

Availability and requirements

Project name: CSA – Chromosome-scale Assembler

Project home page: https://github.com/HMPNK/CSA2.6

Operating system(s): Linux

Programming language: PERL, AWK and BASH scripting

Other requirements: CSA was tested on Ubuntu 18.04/19.04 , Red Hat 8, OpenSuse Leap 15.1, CentOS 7

License: MIT

RRID: SCR_017960

biotoolsID: biotools:CSA2.6

Additional files

Additional file 1: Supplementary tables.

Additional file 2: High-resolution figures.

Additional file 3: Code snapshot used for benchmarks 1-5.

Additional file 4: Code snapshot used for benchmarks 6.

Abbreviations

CPU: Central Processing Unit; CSA: Chromosome-Scale Assembler; ctg: contig; f: chromosomal fusion/fission; i: inversion; interchr.: inter-chromosomal; intrachr.: intra-chromosomal; Hs: Homo sapiens; Mya: Million years ago; ONT: Oxford Nanopore Technologies; PacBio: Pacific Biosciences; RAM: random access memory; scf: scaffold; Sc: *Siniperca chuatsi*; SMRT: Single Molecule Real-Time; t: intra-chromosomal translocation; Tg: *Taeniopygia guttata*; WGS: Whole Genome Shotgun;

Competing interests

The authors declare that they have no competing interests.

Funding information

This work was funded by the German Research Foundation (DFG) “Eigene Stelle” grant within the project “Reference genomes of the Chinese perch (*Siniperca chuatsi*), the Eurasian perch (*Perca fluviatilis*) and three related fish species of the family Sinipercidae for comparative genomics and marker assisted breeding in aquaculture” KU 3596/1-1; project number: 324050651.

Authors contributions

HK designed, programmed and benchmarked the CSA pipeline. **CK** performed independent tests of CSA. **LL**, **XF** and **CK** provided long-read data. **HK** wrote the manuscript with contributions from **SK**, **MS** and **CK**.

Methods

CSA Github project

CSA2.6 and future updates can be downloaded from "https://github.com/HMPNK/CSA2.6". All tools needed to run the pipeline will be installed by a script in the folder "CSA2.6/INSTALL". Simply run "bash INSTALL.bash" and follow the instructions. Some system specific installation issues are mentioned on GitHub. We have tested CSA2.6 on fresh server installations of Red Hat 8 and Ubuntu 18.04/19.04, OpenSuse LEAP 15.1 and CentOS 7 as well as older Red Hat and Ubuntu versions.

Due to ongoing development of the CSA pipeline we provide the code that has been used to benchmark scenarios 1 - 5 and 6 (see supplementary files: "CSA2.6c_benchmarks1-5.tar.gz" and "CSA2.6c_tweaked_ for_ULRs.tar.gz") with this manuscript.

CSA default parameters are currently tweaked for Pacbio RSII and ONT reads (30-60X, N50 readlength 10-30 kbp) We have found that some SEQUEL datasets behave quite different, here adding custom parameters for WTDBG2 will help: -l "-p 0 -k 15 -L5000 -S 2 -A" .

Benchmark scenario 1 – Data and CSA parameters

For the best-case scenario we downloaded reference genomes for *H. sapiens* (GRCh38.p12; RefSeq assembly accession: GCF_000001405.38, here we kept only the chromosomes and removed alternative loci) and *T. guttata* (bTaeGut1_v1.p; RefSeq assembly accession: GCF_003957565.1). For *S. chuatsi* we used our new reference genome sinChu7 (BioProject accession: PRJNA513951). SMRT long-read data for *H. sapiens* was downloaded from the SRA accession: SRP044331. SMRT long-read data for *T. guttata* was downloaded from SRA using the accessions: SRR5224495 - SRR5224503. The SMRT data for *S. chuatsi* will also be available through the BioProject accession: PRJNA513951. All SMRT data were selected for longest subreads and converted to gzip compressed fasta files.

CSA assemblies were run by the following commands:

```
CSA2.6c.pl -r homSap_longest_subreads.fa.gz  -g GRCh38.p12.CHR.fa.gz \
```

```
-t 80 -d HS-GRCh38-2_6C -o HS-GRCh38-2_6C > HS-GRCh38-2_6C.bash
nohup bash HS-GRCh38-2_6C.bash > HS-GRCh38-2_6C.log 2>&1 &

CSA2.6c.pl -r taeGut_SMRT.fa.gz -g bTaeGut1_v1.p.fasta.gz -t 80 \
-o TG-TG-VGP-2_6C -d TG-TG-VGP-2_6C > TG-TG-VGP-2_6C.bash
nohup bash TG-TG-VGP-2_6C.bash > TG-TG-VGP-2_6C.log 2>&1 &

CSA2.6c.pl -r PACBIO-READS-RAW.fa.gz -g sinChu7.fasta -o SC-SC-2_6C \
-d SC-SC-2_6C -t 80 > SC-SC-2_6C.bash
bash SC-SC-2_6C.bash > SC-SC-2_6C.log 2>&1 &
```

Benchmark scenario 2 – Data and CSA parameters

For our diverged reference scenario we downloaded the following genome assemblies.

Mammals: *P. abelii* (Acc: GCF_002880775.1); *C. jacchus* (Acc: GCA_002754865.1); *L. canadensis* (Acc: GCF_007474595.1); *O. anatinus* (Acc: GCF_004115215.1).

Birds: *C. anna* (Acc: GCF_003957555.1); *G. gallus* (Acc: GCF_000002315.6).

Reptile: *A. mississipiensis* (Acc: GCF_000281125.3).

Fish: *P. flavescens* (Acc: GCF_004354835.1).

CSA assemblies were run as above, but omitting the primary assembly step. As the primary assemblies were already calculated under scenario 1 (CSA step1 is a pure *de novo* assembly without support by reference), we can just add the fasta contigs using the parameter –C to save computing time (this procedure would also allow using primary assemblies from other assembly tools than WTDBG2):

H. sapiens

```
CSA2.6c.pl -C HS-GRCh38-2_6C.step1.fa -r homSap_longest_subreads.fa.gz \
 -g GCF_002880775.1_Susie_PABv2_genomic.fna.gz -t 80 -d HS-PA-2_6C \
-o HS-PA-2_6C > HS-PA-2_6C.bash
nohup bash HS-PA-2_6C.bash > HS-PA-2_6C.log 2>&1 &
```

```
CSA2.6c.pl -C HS-GRCh38-2_6C.step1.fa -r homSap_longest_subreads.fa.gz  \
-g GCA_002754865.1_ASM275486v1_genomic.fna.gz -t 80 -d HS-CJ-2_6C \
-o HS-CJ-2_6C > HS-CJ-2_6C.bash
nohup bash HS-CJ-2_6C.bash > HS-CJ-2_6C.log 2>&1 &

CSA2.6c.pl -C HS-GRCh38-2_6C.step1.fa -r homSap_longest_subreads.fa.gz  \
-g mLynCan4_s2.fasta.gz -t 80 -d HS-LC-2_6C -o HS-LC-2_6C > HS-LC-2_6C.bash
nohup bash HS-LC-2_6C.bash > HS-LC-2_6C.log 2>&1 &

CSA2.6c.pl -C HS-GRCh38-2_6C.step1.fa -r homSap_longest_subreads.fa.gz  \
-g GCF_004115215.1_mOrnAna1.p.v1_genomic.fna.gz -t 80 -d HS-OA-2_6C -o HS-
OA-2_6C > HS-OA-2_6C.bash
nohup bash HS-OA-2_6C.bash > HS-OA-2_6C.log 2>&1 &
```

T. guttata

```
CSA2.6c.pl -C TG-TG-VGP-2_6C.step1.fa -r taeGut_SMRT.fa.gz \
-g bCalAnn1_v1.p.fasta.gz -t 80 -o TG-CA-VGP-2_6C \
-d TG-CA-VGP-2_6C > TG-CA-VGP-2_6C.bash
nohup bash TG-CA-VGP-2_6C.bash > TG-CA-VGP-2_6C.log 2>&1 &

CSA2.6c.pl -C TG-TG-VGP-2_6C.step1.fa -r taeGut_SMRT.fa.gz \
-g GCF_000002315.6_GRCg6a_genomic.fna.gz -t 80 -o TG-GG-2_6C \
-d TG-GG-2_6C > TG-GG-2_6C.bash
nohup bash TG-GG-2_6C.bash > TG-GG-2_6C.log 2>&1 &



CSA2.6c.pl -C TG-TG-VGP-2_6C.step1.fa -r taeGut_SMRT.fa.gz \
-g GCF_000281125.3_ASM28112v4_genomic.fna.gz -t 80 -o TG-AM-2_6C \
-d TG-AM-2_6C > TG-AM-2_6C.bash
```

```
nohup bash TG-AM-2_6C.bash > TG-AM-2_6C.log 2>&1 &
```

S. chuatsi

```
CSA2.6c.pl -C SC-SC-2_6C.step1.fa -r PACBIO-READS-RAW.fa.gz  \
-g GCF_004354835.1_PFLA_1.0_genomic.fna.gz -o SC-PFLA-2_6C \
-d SC-PFLA-2_6C -t 80 > SC-PFLA-2_6C.bash
nohup bash SC-PFLA-2_6C.bash > SC-PFLA-2_6C.log 2>&1 &
```

Benchmark scenario 3 – Data and CSA parameters

To assemble the *Perca fluviatilis* genome by CSA we used the *P. fluviatilis* reference genome (BioProject Acc: PRJNA549142) for the best case scenario. A 10X Genomics Supernova assembly (Acc: GCA_003412525.1) of *P. fluviatilis* as well as the *P. flavescens* and the *S. chuatsi* genomes from above were used to benchmark CSA using multiple references sequentially. Oxford Nanopore long-read data for *P. fluviatilis* was obtained from (BioProject Acc: PRJNA549142). CSA parameters for the best case scenario were:

```
CSA2.6c.pl -r perFlu_ONT_ALL.fa.gz \
-g Perca_fluviatilis.PFLU1.1.dna.toplevel.fa.gz -t 80 -o PF-PF-HiC-2_6C \
-d PF-PF-HiC-2_6C > PF-PF-HiC-2_6C.bash
nohup bash PF-PF-HiC-2_6C.bash > PF-PF-HiC-2_6C.log 2>&1 &
```

Again for the assembly using multiple references, we used the primary contigs from above (-C), now adding the reference sequences for sequential improvement as a comma separated list (e.g. -g closest.fa,less_diverged.fa,most_diverged.fa):

```
CSA2.6c.pl -C PF-PF-HiC-2_6C.step1.fa -r perFlu_ONT_ALL.fa.gz \
-g GCA_003412525.1_UTU_Pfluv_1.1_genomic.fna.gz,\
Perca_flavescens.PFLA1.1.dna.toplevel.fa.gz,sinChu7.fasta -t 80 \
-o PF-10X-PFLA-SC-2_6C -d PF-10X-PFLA-SC-2_6C > PF-10X-PFLA-SC-2_6C.bash
nohup bash PF-10X-PFLA-SC-2_6C.bash > PF-10X-PFLA-SC-2_6C.log 2>&1 &
```

```
CSA2.6c.pl -C PF-PF-HiC-2_6C.step1.fa -r perFlu_ONT_ALL.fa.gz \
```

```
-g Perca_flavescens.PFLA1.1.dna.toplevel.fa.gz,sinChu7.fasta -t 80 \
-o PF-PFLA-SC-2_6C -d PF-PFLA-SC-2_6C > PF-PFLA-SC-2_6C.bash
nohup bash PF-PFLA-SC-2_6C.bash > PF-PFLA-SC-2_6C.log 2>&1 &

CSA2.6c.pl -C PF-PF-HiC-2_6C/01_WTDBG/PF-PF-HiC-2_6C.step1.fa \
-r ../DATA/perFlu/perFlu_ONT_ALL.fa.gz -g ../REFERENCES/sinChu7.fasta \
-t 80 -o PF-SC-2_6C -d PF-SC-2_6C > PF-SC-2_6C.bash
nohup bash PF-SC-2_6C.bash > PF-SC-2_6C.log 2>&1
```

Benchmark scenario 4 – Data and CSA parameters

Here we used the relatively low N50 contig length primary assembly of *P. fluviatilis* from scenario 3 to assemble the *S. chuatsi* SMRT data from above:

```
CSA2.6c.pl -C SC-SC-2_6C.step1.fa -r PACBIO-READS-RAW.fa.gz  \
-g PF-PF-HiC-2_6C.step1.fa -o SC-PFdraft-2_6C -d SC-PFdraft-2_6C \
-t 80 > SC-PFdraft-2_6C.bash
nohup bash SC-PFdraft-2_6C.bash > SC-PFdraft-2_6C.log 2>&1 &
```

Benchmark scenario 5 – Data and CSA parameters

To get subsets of the *H. sapiens* SMRT data we used SEQTK to randomly subsample reads from the full dataset in a way that we obtained about 40x, 30x, 20x and 15x sequencing coverage. We ran CSA with these read sets using either the GRCh38 genome (best-case) or the *P. abelii* genome (diverged reference) as reference.

Best-case:

```
CSA2.6c.pl -r hs15x.fa.gz  -g GRCh38.p12.CHR.fa.gz -t 80 \
-d HS25-GRCh38-2_6C -o HS25-GRCh38-2_6C > HS25-GRCh38-2_6C.bash
nohup bash HS25-GRCh38-2_6C.bash > HS25-GRCh38-2_6C.log 2>&1 &

CSA2.6c.pl -r hs20x.gz  -g GRCh38.p12.CHR.fa.gz -t 80 -d HS33-GRCh38-2_6C \
-o HS33-GRCh38-2_6C > HS33-GRCh38-2_6C.bash
nohup bash HS33-GRCh38-2_6C.bash > HS33-GRCh38-2_6C.log 2>&1 &
```

```
CSA2.6c.pl -r hs30x.fa.gz  -g GRCh38.p12.CHR.fa.gz -t 80 \
-d HS50-GRCh38-2_6C -o HS50-GRCh38-2_6C > HS50-GRCh38-2_6C.bash
nohup bash HS50-GRCh38-2_6C.bash > HS50-GRCh38-2_6C.log 2>&1 &

CSA2.6c.pl -r hs40x.fa.gz  -g GRCh38.p12.CHR.fa.gz -t 80 \
-d HS67-GRCh38-2_6C -o HS67-GRCh38-2_6C > HS67-GRCh38-2_6C.bash
nohup bash HS67-GRCh38-2_6C.bash > HS67-GRCh38-2_6C.log 2>&1 &
```

Diverged reference:

```
CSA2.6c.pl -C HS25-GRCh38-2_6C.step1.fa -r hs 15x.fa.gz \
-g GCF_002880775.1_Susie_PABv2_genomic.fna.gz -t 80 -d HS25-PA-2_6C \
-o HS25-PA-2_6C > HS25-PA-2_6C.bash
nohup bash HS25-PA-2_6C.bash > HS25-PA-2_6C.log 2>&1 &

CSA2.6c.pl -C HS33-GRCh38-2_6C.step1.fa -r hs 20x.fa.gz \
-g GCF_002880775.1_Susie_PABv2_genomic.fna.gz -t 80 -d HS33-PA-2_6C \
-o HS33-PA-2_6C > HS33-PA-2_6C.bash
nohup bash HS33-PA-2_6C.bash > HS33-PA-2_6C.log 2>&1 &

CSA2.6c.pl -C HS50-GRCh38-2_6C.step1.fa -r hs 30x.fa.gz \
-g GCF_002880775.1_Susie_PABv2_genomic.fna.gz -t 80 -d HS50-PA-2_6C \
-o HS50-PA-2_6C > HS50-PA-2_6C.bash
nohup bash HS50-PA-2_6C.bash > HS50-PA-2_6C.log 2>&1 &

CSA2.6c.pl -C HS67-GRCh38-2_6C.step1.fa -r hs 40x.fa.gz \
-g GCF_002880775.1_Susie_PABv2_genomic.fna.gz -t 80 -d HS67-PA-2_6C \
-o HS67-PA-2_6C > HS67-PA-2_6C.bash
nohup bash HS67-PA-2_6C.bash > HS67-PA-2_6C.log 2>&1 &
```

Benchmark scenario 6 – CSA using ultra-long reads

Ultra-long reads (ULR, N50 readlength > 50 kbp) from Oxford Nanopore sequencing will be available to many researchers soon. CSA default parameters are currently tweaked for common SMRT or ONT long-read data (N50<30 kbp). Nevertheless, two CSA parameters may be set to improve ultra-long-read assembly:

A) Set '-p 2' to circumvent issues with the wtdbg-cns tool that might otherwise crash on ultra-long reads. CSA uses the "-p" option to set the WTDBG2 consensus caller (0 = wtdbg-cons (default); 1 = wtpoa-cons; consensus calculation in step 1; 2 = wtdbg-cons -S 0; wtpoa-cns is slower but a bit more accurate; wtdbg-cns with option -S 0 is more stable on very long reads.

B) Set '-l "-L 70000 --aln-min-length 25000 --keep-multiple-alignment-parts 1 –A' to vastly improve contig N50 on ultra-long read datasets. Make sure you still have enough coverage (e.g. ~30x) left when skipping reads with length below 70000bp, otherwise try –L 60000 or –L 50000 and so on. CSA uses the –l "…" parameter to pass on detailed parameters to the wtdbg2 assembler. Parameters provided by –l "…" may overrule other wtdbg2 parameters set by CSA (e.g. -k, -s, -e or -m).

We downloaded ULR data (CHM13 cell line) for benchmarking from the Telomere-to-Telomere (T2T) consortium:

https://s3.amazonaws.com/nanopore-human-wgs/chm13/nanopore/rel2/rel2.fastq.gz

We also downloaded the SHASTA genome assembly derived from this data for comparisons:

https://s3-us-west-2.amazonaws.com/human-pangenomics/assemblies/raw/shasta/CHM13.shasta.fasta

CSA was run with the following parameters:

```
CSA2.6c.pl -r rel2.fa.gz  -g GRCh38.p12.CHR.fa.gz -t 72 -d HS-ULR-2_6C \
-o HS-ULR-2_6C -p 2 -l "-L 70000 --aln-min-length 25000 \
--keep-multiple-alignment-parts 1 -A" > HS-ULR-2_6C.bash
nohup bash HS-ULR-2_6C.bash > HS-ULR-2_6C.log 2>&1
```

Dot plots and assembly comparisons

All CSA assemblies were compared to state-of-the-art reference genomes of the same species by MINIMAP2 using parameters for slightly diverged assembly-to-reference mapping (-x asm20, as we are dealing with unpolished consensus sequences here). PAF output files were filtered for MQ 60 (most unique) alignments and plotted by MINIDOT. PAF files were also analysed by custom scripts to combine splitted neighbouring alignments and count large scale (>300 kb) fusions (or inter-chromosomal translocations), intra-chromosomal transloctions and inversions.

Using CSA to close gaps in an existing scaffolded assembly by long reads

Users might want to use CSA only for gap closing their existing scaffolded assemblies. They may split their scaffolds in contigs and then parametrize CSA with these contigs, while using the scaffolded contigs as reference (e.g. "…. -C contigs_from_scaffolds.fa -g scaffolds.fa –r longreads.fa.gz….").

It is also possible to use the last gap closure step (in CSA-step4), which looks for neighbouring contig overlaps in scaffolds as a stand-alone procedure:

```
bash /your_path/CSA2.6/INSTALL/../script/STITCH.sh scaffolds.fa \
/your_path/CSA2.6/INSTALL/.. > scaffolds_with_joined_overlapping_contigs.fa
```

References

1. Gordon D, Huddleston J, Chaisson MJ, Hill CM, Kronenberg ZN, Munson KM, et al. Long-read sequence assembly of the gorilla genome. Science. 2016;352 6281:aae0344. doi:10.1126/science.aae0344.
2. Vij S, Kuhl H, Kuznetsova IS, Komissarov A, Yurchenko AA, Van Heusden P, et al. Chromosomal-Level Assembly of the Asian Seabass Genome Using Long Sequence Reads and Multi-layered Scaffolding. PLoS Genet. 2016;12 4:e1005954. doi:10.1371/journal.pgen.1005954.
3. Korlach J, Gedman G, Kingan SB, Chin CS, Howard JT, Audet JN, et al. De novo PacBio long-read and phased avian genome assemblies correct and add to reference genes generated with intermediate and short reads. Gigascience. 2017;6 10:1-16. doi:10.1093/gigascience/gix085.
4. Myers EW, Sutton GG, Delcher AL, Dew IM, Fasulo DP, Flanigan MJ, et al. A whole-genome assembly of *Drosophila*. Science. 2000;287 5461:2196-204. doi:10.1126/science.287.5461.2196.
5. Batzoglou S, Jaffe DB, Stanley K, Butler J, Gnerre S, Mauceli E, et al. ARACHNE: a whole-genome shotgun assembler. Genome Res. 2002;12 1:177-89. doi:10.1101/gr.208902.
6. Huang X, Wang J, Aluru S, Yang SP and Hillier L. PCAP: a whole-genome assembly program. Genome Res. 2003;13 9:2164-70. doi:10.1101/gr.1390403.
7. Margulies M, Egholm M, Altman WE, Attiya S, Bader JS, Bemben LA, et al. Genome sequencing in microfabricated high-density picolitre reactors. Nature. 2005;437 7057:376-80. doi:10.1038/nature03959.
8. Gnerre S, Maccallum I, Przybylski D, Ribeiro FJ, Burton JN, Walker BJ, et al. High-quality draft assemblies of mammalian genomes from massively parallel sequence data. Proc Natl Acad Sci U S A. 2011;108 4:1513-8. doi:10.1073/pnas.1017351108.
9. Luo R, Liu B, Xie Y, Li Z, Huang W, Yuan J, et al. SOAPdenovo2: an empirically improved memory-efficient short-read de novo assembler. Gigascience. 2012;1 1:18. doi:10.1186/2047-217X-1-18.
10. Chin CS, Peluso P, Sedlazeck FJ, Nattestad M, Concepcion GT, Clum A, et al. Phased diploid genome assembly with single-molecule real-time sequencing. Nat Methods. 2016;13 12:1050-4. doi:10.1038/nmeth.4035.
11. Koren S, Walenz BP, Berlin K, Miller JR, Bergman NH and Phillippy AM. Canu: scalable and accurate long-read assembly via adaptive k-mer weighting and repeat separation. Genome Res. 2017;27 5:722-36. doi:10.1101/gr.215087.116.
12. Kolmogorov M, Yuan J, Lin Y and Pevzner PA. Assembly of long, error-prone reads using repeat graphs. Nat Biotechnol. 2019;37 5:540-6. doi:10.1038/s41587-019-0072-8.
13. Li H. Minimap and miniasm: fast mapping and de novo assembly for noisy long sequences. Bioinformatics. 2016;32 14:2103-10. doi:10.1093/bioinformatics/btw152.
14. Ruan J and Li H. Fast and accurate long-read assembly with wtdbg2. bioRxiv. 2019.
15. Burton JN, Adey A, Patwardhan RP, Qiu R, Kitzman JO and Shendure J. Chromosome-scale scaffolding of de novo genome assemblies based on chromatin interactions. Nat Biotechnol. 2013;31 12:1119-25. doi:10.1038/nbt.2727.
16. Ghurye J, Pop M, Koren S, Bickhart D and Chin CS. Scaffolding of long read assemblies using long range contact information. BMC Genomics. 2017;18 1:527. doi:10.1186/s12864-017-3879-z.
17. Ghurye J and Pop M. Modern technologies and algorithms for scaffolding assembled genomes. PLoS Comput Biol. 2019;15 6:e1006994. doi:10.1371/journal.pcbi.1006994.
18. Howe K and Wood JM. Using optical mapping data for the improvement of vertebrate genome assemblies. Gigascience. 2015;4:10. doi:10.1186/s13742-015-0052-y.

19. Fierst JL. Using linkage maps to correct and scaffold de novo genome assemblies: methods, challenges, and computational tools. Front Genet. 2015;6:220. doi:10.3389/fgene.2015.00220.
20. Meyer A and Van de Peer Y. From 2R to 3R: evidence for a fish-specific genome duplication (FSGD). Bioessays. 2005;27 9:937-45. doi:10.1002/bies.20293.
21. Sacerdot C, Louis A, Bon C, Berthelot C and Roest Crollius H. Chromosome evolution at the origin of the ancestral vertebrate genome. Genome Biol. 2018;19 1:166. doi:10.1186/s13059-018-1559-1.
22. Gregory TR. Synergy between sequence and size in large-scale genomics. Nat Rev Genet. 2005;6 9:699-708. doi:10.1038/nrg1674.
23. Voss SR, Kump DK, Putta S, Pauly N, Reynolds A, Henry RJ, et al. Origin of amphibian and avian chromosomes by fission, fusion, and retention of ancestral chromosomes. Genome Res. 2011;21 8:1306-12. doi:10.1101/gr.116491.110.
24. Ruiz-Herrera A, Farre M and Robinson TJ. Molecular cytogenetic and genomic insights into chromosomal evolution. Heredity (Edinb). 2012;108 1:28-36. doi:10.1038/hdy.2011.102.
25. Irimia M, Tena JJ, Alexis MS, Fernandez-Minan A, Maeso I, Bogdanovic O, et al. Extensive conservation of ancient microsynteny across metazoans due to cis-regulatory constraints. Genome Res. 2012;22 12:2356-67. doi:10.1101/gr.139725.112.
26. Zimmermann B, Robert NSM, Technau U and Simakov O. Ancient animal genome architecture reflects cell type identities. Nat Ecol Evol. 2019;3 9:1289-93. doi:10.1038/s41559-019-0946-7.
27. Braasch I, Gehrke AR, Smith JJ, Kawasaki K, Manousaki T, Pasquier J, et al. The spotted gar genome illuminates vertebrate evolution and facilitates human-teleost comparisons. Nat Genet. 2016;48 4:427-37. doi:10.1038/ng.3526.
28. Ravi V and Venkatesh B. The Divergent Genomes of Teleosts. Annu Rev Anim Biosci. 2018;6:47-68. doi:10.1146/annurev-animal-030117-014821.
29. Nowoshilow S, Schloissnig S, Fei JF, Dahl A, Pang AWC, Pippel M, et al. The axolotl genome and the evolution of key tissue formation regulators. Nature. 2018;554 7690:50-5. doi:10.1038/nature25458.
30. Smith JJ, Timoshevskaya N, Timoshevskiy VA, Keinath MC, Hardy D and Voss SR. A chromosome-scale assembly of the axolotl genome. Genome Res. 2019;29 2:317-24. doi:10.1101/gr.241901.118.
31. Hellsten U, Harland RM, Gilchrist MJ, Hendrix D, Jurka J, Kapitonov V, et al. The genome of the Western clawed frog *Xenopus tropicalis*. Science. 2010;328 5978:633-6. doi:10.1126/science.1183670.
32. Sun YB, Xiong ZJ, Xiang XY, Liu SP, Zhou WW, Tu XL, et al. Whole-genome sequence of the Tibetan frog *Nanorana parkeri* and the comparative evolution of tetrapod genomes. Proc Natl Acad Sci U S A. 2015;112 11:E1257-62. doi:10.1073/pnas.1501764112.
33. Pokorna M, Giovannotti M, Kratochvil L, Caputo V, Olmo E, Ferguson-Smith MA, et al. Conservation of chromosomes syntenic with avian autosomes in squamate reptiles revealed by comparative chromosome painting. Chromosoma. 2012;121 4:409-18. doi:10.1007/s00412-012-0371-z.
34. Deakin JE and Ezaz T. Understanding the Evolution of Reptile Chromosomes through Applications of Combined Cytogenetics and Genomics Approaches. Cytogenet Genome Res. 2019;157 1-2:7-20. doi:10.1159/000495974.
35. Farre M, Kim J, Proskuryakova AA, Zhang Y, Kulemzina AI, Li Q, et al. Evolution of gene regulation in ruminants differs between evolutionary breakpoint regions and homologous synteny blocks. Genome Res. 2019;29 4:576-89. doi:10.1101/gr.239863.118.
36. Nanda I, Shan Z, Schartl M, Burt DW, Koehler M, Nothwang H, et al. 300 million years of conserved synteny between chicken Z and human chromosome 9. Nat Genet. 1999;21 3:258-9. doi:10.1038/6769.

37. Catchen JM, Conery JS and Postlethwait JH. Automated identification of conserved synteny after whole-genome duplication. Genome Res. 2009;19 8:1497-505. doi:10.1101/gr.090480.108.
38. Zhao T and Schranz ME. Network-based microsynteny analysis identifies major differences and genomic outliers in mammalian and angiosperm genomes. Proc Natl Acad Sci U S A. 2019;116 6:2165-74. doi:10.1073/pnas.1801757116.
39. Zhang G. The bird's-eye view on chromosome evolution. Genome Biol. 2018;19 1:201. doi:10.1186/s13059-018-1585-z.
40. Kim J, Larkin DM, Cai Q, Asan, Zhang Y, Ge RL, et al. Reference-assisted chromosome assembly. Proc Natl Acad Sci U S A. 2013;110 5:1785-90. doi:10.1073/pnas.1220349110.
41. Kolmogorov M, Armstrong J, Raney BJ, Streeter I, Dunn M, Yang F, et al. Chromosome assembly of large and complex genomes using multiple references. Genome Res. 2018;28 11:1720-32. doi:10.1101/gr.236273.118.
42. Bhutkar A, Russo S, Smith TF and Gelbart WM. Techniques for multi-genome synteny analysis to overcome assembly limitations. Genome Inform. 2006;17 2:152-61.
43. Anselmetti Y, Berry V, Chauve C, Chateau A, Tannier E and Berard S. Ancestral gene synteny reconstruction improves extant species scaffolding. BMC Genomics. 2015;16 Suppl 10:S11. doi:10.1186/1471-2164-16-S10-S11.
44. Song G, Lee J, Kim J, Kang S, Lee H, Kwon D, et al. Integrative Meta-Assembly Pipeline (IMAP): Chromosome-level genome assembler combining multiple de novo assemblies. PLoS One. 2019;14 8:e0221858. doi:10.1371/journal.pone.0221858.
45. Koepfli KP, Paten B, Genome KCoS and O'Brien SJ. The Genome 10K Project: a way forward. Annu Rev Anim Biosci. 2015;3:57-111. doi:10.1146/annurev-animal-090414-014900.
46. Lewin HA, Robinson GE, Kress WJ, Baker WJ, Coddington J, Crandall KA, et al. Earth BioGenome Project: Sequencing life for the future of life. Proc Natl Acad Sci U S A. 2018;115 17:4325-33. doi:10.1073/pnas.1720115115.
47. Li H. Minimap2: pairwise alignment for nucleotide sequences. Bioinformatics. 2018;34 18:3094-100. doi:10.1093/bioinformatics/bty191.
48. Frith MC and Kawaguchi R. Split-alignment of genomes finds orthologies more accurately. Genome Biol. 2015;16:106. doi:10.1186/s13059-015-0670-9.
49. Kolmogorov M, Raney B, Paten B and Pham S. Ragout-a reference-assisted assembly tool for bacterial genomes. Bioinformatics. 2014;30 12:i302-9. doi:10.1093/bioinformatics/btu280.
50. Zhu S, Chen DZ and Emrich SJ. Single molecule sequencing-guided scaffolding and correction of draft assemblies. BMC Genomics. 2017;18 Suppl 10:879. doi:10.1186/s12864-017-4271-8.
51. Lam KK, Hall R, Clum A and Rao S. BIGMAC : breaking inaccurate genomes and merging assembled contigs for long read metagenomic assembly. BMC Bioinformatics. 2016;17 1:435. doi:10.1186/s12859-016-1288-y.
52. Bosi E, Donati B, Galardini M, Brunetti S, Sagot MF, Lio P, et al. MeDuSa: a multi-draft based scaffolder. Bioinformatics. 2015;31 15:2443-51. doi:10.1093/bioinformatics/btv171.
53. Kurtz S, Phillippy A, Delcher AL, Smoot M, Shumway M, Antonescu C, et al. Versatile and open software for comparing large genomes. Genome Biol. 2004;5 2:R12. doi:10.1186/gb-2004-5-2-r12.
54. English AC, Richards S, Han Y, Wang M, Vee V, Qu J, et al. Mind the gap: upgrading genomes with Pacific Biosciences RS long-read sequencing technology. PLoS One. 2012;7 11:e47768. doi:10.1371/journal.pone.0047768.
55. Xu GC, Xu TJ, Zhu R, Zhang Y, Li SQ, Wang HW, et al. LR_Gapcloser: a tiling path-based gap closer that uses long reads to complete genome assembly. Gigascience. 2019;8 1 doi:10.1093/gigascience/giy157.
56. Kapusta A, Suh A and Feschotte C. Dynamics of genome size evolution in birds and mammals. Proc Natl Acad Sci U S A. 2017;114 8:E1460-E9. doi:10.1073/pnas.1616702114.
57. Wang Z, Zhang J, Yang W, An N, Zhang P, Zhang G, et al. Temporal genomic evolution of bird sex chromosomes. BMC Evol Biol. 2014;14:250. doi:10.1186/s12862-014-0250-8.

58. Ozerov MY, Ahmad F, Gross R, Pukk L, Kahar S, Kisand V, et al. Highly Continuous Genome Assembly of Eurasian Perch (*Perca fluviatilis*) Using Linked-Read Sequencing. G3 (Bethesda). 2018;8 12:3737-43. doi:10.1534/g3.118.200768.
59. Feron R, Zahm M, Cabau C, Klopp C, Roques C, Bouchez O, et al. Characterization of a Y-specific duplication/insertion of the anti-Mullerian hormone type II receptor gene based on a chromosome-scale genome assembly of yellow perch, *Perca flavescens*. bioRxiv. 2019.
60. Walker BJ, Abeel T, Shea T, Priest M, Abouelliel A, Sakthikumar S, et al. Pilon: an integrated tool for comprehensive microbial variant detection and genome assembly improvement. PLoS One. 2014;9 11:e112963. doi:10.1371/journal.pone.0112963.
61. Simao FA, Waterhouse RM, Ioannidis P, Kriventseva EV and Zdobnov EM. BUSCO: assessing genome assembly and annotation completeness with single-copy orthologs. Bioinformatics. 2015;31 19:3210-2. doi:10.1093/bioinformatics/btv351.
62. Shafin K, Pesout T, Lorig-Roach R, Haukness M, Olsen HE, Bosworth C, et al. Efficient *de novo* assembly of eleven human genomes using PromethION sequencing and a novel nanopore toolkit. bioRxiv. 2019.

Supplementary tables

Supplementary Table 1: CSA results of the best case scenario, for representative genomes of mammals, birds and fish.

	vertebrate clade	Mammalia	Aves	Teleostei
CSA setup	species	***Homo sapiens***	***Taeniopygia guttata***	***Siniperca chuatsi***
	species haploid chr count=n	23	40	24
	input data type: seq. coverage; N50 read length	SMRT: 60-fold; N50:20 kb	SMRT: 96-fold; N50:19 kb	SMRT: 50-fold; N50:12 kb
	benchmark scenario	best case	best case	best case
	reference	*H.sapiens GRCh38*	*T.guttata*	*S. chuatsi*
	reference divergence time	0.0	0.0	0.0
	reference haploid chr count	23	~40	24
CSA step1	total contig length	2,846,783,372	1,099,596,476	721,123,858
	contig N50	15,637,873	17,162,677	11,615,497
	max. contig length	103,078,150	65,902,548	30,768,821
CSA step2	placed in top n chr	97.44%	93.99%	98.40%
	scaffold N50	151,200,468	71,399,975	30,139,544
	max. scaffold length	234,201,754	151,322,278	38,172,652
CSA step3	total contig length	2,849,859,767	1,096,751,084	721,496,232
	contig N50	25,894,807	26,171,859	13,428,662
	max. contig length	109,927,675	72,222,397	30,765,277
CSA final	total scaffold length	2,866,335,788	1,099,655,660	726,281,108
	total contig length	2,849,495,757	1,096,712,419	715,472,342
	placed in top n chr	97.50%	94.24%	99.33%
	scaffold N50	150,569,357	71,372,070	30,013,467
	contig N50	25,894,807	27,655,297	16,495,661
	max. scaffold length	233,785,065	151,378,290	38,160,875
	max. contig length	109,927,675	72,222,397	30,765,277
	runtime server (80 threads E7-8890v4@2.20GHz)	16h	5h:45m	2h:30m
	contig N50 improvement over CSA step1 [x-fold]	1.66	1.61	1.42
	contig N50 impr. over best published SMRT assembly [x-fold]	0.98	2.30	1.35
Errors scf	fusions; intra-chr. translocations; inversions (blocks >300kbp)	f:0; t:6; i:2;	f:0; t:1; i:4	f:0(1); t:1; i:5(6)
Errors ctg		f:0; t:2; i:0	f:0; t:1; i:4	f:0(1); t:0; i:3(4)

Supplementary Table 2: CSA results using divergent reference genomes

CSA setup	**species**	*Homo sapiens*	*Homo sapiens*	*Homo sapiens*	*Homo sapiens*	*Taeniopygia guttata*	*Taeniopygia guttata*	*Taeniopygia guttata*	*Siniperca chuatsi*	*Siniperca chuatsi*
	**species haploid chr count=n**	23	23	23	23	40	40	40	24	24
	**input data type: seq. coverage; N50 read length**	SMRT: 60-fold; N50:20 kb	SMRT: 60-fold; N50:20 kb	SMRT: 60-fold; N50:20 kb	SMRT: 60-fold; N50:20 kb	SMRT: 96-fold; N50:19 kb	SMRT: 96-fold; N50:19 kb	SMRT: 96-fold; N50:19 kb	SMRT: 50-fold; N50:12 kb	SMRT: 50-fold; N50:12 kb
	**benchmark scenario**	diverged ref.	diverged ref.	diverged ref.	diverged ref.	diverged reference	diverged reference	diverged draft assembly	diverged reference	draft contig reference
	**reference**	*P. abelii*	*C. jacchus*	*L. canadensis*	*O. anatinus*	*C. anna*	*G. gallus*	*A. mississipiensis*	*P. flavescens*	*P. fluviatilis* (CSA step1 contigs; N50: 2.8Mbp)
	**reference divergence time**	15.8	42.9	94.0	180.0	65.0	80.0	240.0	65.0	65.0
	**reference haploid chr count**	24	22	19	27	>40	~39	16	24	24
CSA step1	**total contig length**	2,846,783,372	2,846,783,372	2,846,783,372	2,846,783,372	1,099,596,476	1,099,596,476	1,099,596,476	721,123,858	721,123,858
	**contig N50**	15,637,873	15,637,873	15,637,873	15,637,873	17,162,677	17,162,677	17,162,677	11,615,497	11,615,497
	**max. contig length**	103,078,150	103,078,150	103,078,150	103,078,150	65,902,548	65,902,548	65,902,548	30,768,821	30,768,821
CSA step2	**placed in top n chr**	93.76%	96.00%	95.83%	92.51%	92.04%	92.77%	90.48%	93.92%	76.04%
	**scaffold N50**	128,353,462	129,800,600	167,706,686	127,367,631	55,664,037	74,226,408	53,874,258	29,495,621	22,961,239
	**max. scaffold length**	219,829,795	210,205,854	392,920,739	307,297,870	146,901,896	186,662,268	115,106,231	38,170,751	36,597,929
CSA step3	**total contig length**	2,849,302,170	2,848,630,351	2,848,648,159	2,849,085,066	1,096,419,901	1,096,584,403	1,096,413,559	721,051,678	720,977,241
	**contig N50**	25,506,235	24,655,207	25,316,480	24,550,351	18,624,647	19,226,526	18,624,651	14,142,781	13,428,662
	**max. contig length**	106,332,896	106,583,100	104,396,992	108,663,965	65,887,603	65,887,602	65,887,602	30,764,502	30,764,502
CSA final	**total scaffold length**	2,858,013,933	2,856,072,174	2,856,023,627	2,857,274,643	1,097,447,182	1,097,814,908	1,098,232,426	724,064,917	721,968,017
	**total contig length**	2,849,099,702	2,848,553,253	2,848,553,748	2,849,048,046	1,096,370,479	1,096,530,772	1,096,361,835	721,014,191	720,954,856
	**placed in top n chr**	93.84%	96.07%	96.10%	91.62%	92.13%	93.11%	90.56%	94.73%	77.26%
	**scaffold N50**	127,999,133	129,879,741	167,642,951	127,324,216	55,672,797	74,887,648	53,831,915	29,407,777	23,439,636
	**contig N50**	29,334,513	24,749,570	28,128,276	25,651,180	18,882,724	19,248,331	19,226,897	16,688,192	16,688,192
	**max. scaffold length**	219,825,495	210,340,950	393,325,976	307,230,484	146,917,470	186,491,133	114,959,208	38,156,639	36,593,613
	**max. contig length**	109,935,533	106,583,100	104,396,992	108,663,965	65,887,603	65,887,602	65,887,602	35,806,844	35,807,018
	**runtime server (80 threads E7-8890v4@2.20GHz)**	15h30m	13h:30m	13h	12h:40m	5h:41m	5h:46m	5h:52m	2h:30m	2h:20m
	**contig N50 improvement over CSA step1 [x-fold]**	1.88	1.58	1.80	1.64	1.10	1.12	1.12	1.44	1.44
	**contig N50 impr. over best published SMRT assembly [x-fold]**	1.12	0.94	1.07	0.98	1.57	1.60	1.60	1.37	1.37
Errors scf	**fusions; intra-chr. translocations; inversions (blocks >300kbp)**	f:0; t:58; i:8	f:11; t:72; i:12	f:15; t:78; i:12	f:47; t:87; i:23	f:1; t:33; i:20	f:2; t:26; i:20	f:2; t:22; i:18	f:1(2); t:13; i:16(17)	f:0(1); t:2; i:9(10)
Errors ctg		f:0; t:4; i:6	f:0; t:4; i:7	f:0; t:5; i:10	f:1; t:5; i:12	f:0; t:1; i:6	f:0; t:1; i:8	f:0; t:1; i:6	f:0(1); t:0; i:5(6)	f:0(1); t:0; i:5(6)

Supplementary Table 3: ONT read assembly supported by 10X Genomics, genus-level and order-level references.

	vertebrate clade	Teleostei	Teleostei	Teleostei	Teleostei
CSA setup	species	*Perca fluviatilis*	*Perca fluviatilis*	*Perca fluviatilis*	*Perca fluviatilis*
	species haploid chr count=n	24	24	24	24
	input data type: seq. coverage; N50 read length	ONT: 67-fold; N50: 12 kb	ONT: 67-fold; N50: 12 kb	ONT: 67-fold; N50: 12 kb	ONT: 67-fold; N50: 12 kb
	benchmark scenario	best case	10X genomics + two diverged references	two diverged references	diverged reference
	reference	*Perca fluviatilis HiC*	*10X P.fluviatilis; P. flavescens; S.chuatsi*	*P. flavescens; S.chuatsi*	*S.chuatsi*
	reference divergence time	0.0	0,0; 10-20; 65;	10-20; 65;	65.0
	reference haploid chr count	24	24	24	24
CSA step1	total contig length	929,045,493	929,045,493	929,045,493	929,045,493
	contig N50	2,816,703	2,816,703	2,816,703	2,816,703
	max. contig length	15,906,396	15,906,396	15,906,396	15,906,396
CSA step2	placed in top n chr	95.89%	93.89%	93.10%	86.12%
	scaffold N50	38,622,337	37,697,155	37,043,440	33,865,843
	max. scaffold length	47,653,006	47,519,747	46,927,054	46,802,501
CSA step3	total contig length	929,510,545	929,018,531	929,069,033	928,502,336
	contig N50	5,237,589	4,684,879	5,007,526	4,699,930
	max. contig length	24,199,483	24,200,308	24,198,405	24,792,848
CSA final	total scaffold length	944,861,078	943,065,049	942,130,566	936,090,143
	total contig length	929,241,819	928,740,496	928,809,152	928,301,535
	placed in top n chr	96.15%	94.28%	93.79%	86.34%
	scaffold N50	38,362,302	37,309,771	36,955,123	33,805,383
	contig N50	7,574,134	8,008,293	7,745,610	7,050,314
	max. scaffold length	47,026,637	46,017,986	46,666,870	46,417,396
	max. contig length	27,361,245	27,581,946	27,582,002	27,361,483
	runtime server (80 threads E7-8890v4@2.20GHz)	5h:02m	5h:30m	5h:15m	5h:15m
	contig N50 improvement over CSA step1 [x-fold]	2.69	2.84	2.75	2.50
	contig N50 impr. over best published ONT assembly [x-fold]	2.92	3.09	2.99	2.72
Errors scf	fusions; intra-chr. translocations; inversions (blocks >300kbp)	f:0; t:0; i:3	f:0; t:12; i:13	f:0; t:19; i:15	f:0; t:57; i:16
Errors ctg		f:0; t:0; i:1	f:0; t:0; i:4	f:0; t:0; i:3	f:0; t:0; i:4

Supplementary Table 4: Influence of sequencing coverage on *H. sapiens* CSA assemblies.

	vertebrate clade	Mammalia	Mammalia	Mammalia	Mammalia	Mammalia
CSA setup	species	*Homo sapiens*	*Homo sapiens*	*Homo sapiens*	*Homo sapiens*	*Homo sapiens*
	species haploid chr count=n	23	23	23	23	23
	input data type: seq. coverage; N50 read length	SMRT: 60-fold; N50:20 kb	SMRT: 40-fold; N50:20 kb	SMRT: 30-fold; N50:20 kb	SMRT: 20-fold; N50:20 kb	SMRT: 15-fold; N50:20 kb
	benchmark scenario	best case	lower coverage	lower coverage	lower coverage	lower coverage
	reference	*H.sapiens GRCh38*	*H.sapiens GRCh38*	*H.sapiens GRCh38*	*H.sapiens GRCh38*	*H.sapiens GRCh38*
	reference divergence time	0.0	0	0.0	0.0	0.0
	reference haploid chr count	23	23	23	23	23
CSA step1	total contig length	2,846,783,372	2,840,640,553	2,841,463,516	2,847,406,287	2,826,278,103
	contig N50	15,637,873	16,341,163	12,497,249	4,383,406	1,299,535
	max. contig length	103,078,150	88,948,008	82,332,068	27,279,889	11,252,181
CSA step2	placed in top n chr	97.44%	97.60%	97.96%	96.85%	96.80%
	scaffold N50	151,200,468	151,424,426	151,231,434	142,490,860	143,351,851
	max. scaffold length	234,201,754	235,703,142	234,220,726	235,695,545	236,751,258
CSA step3	total contig length	2,849,859,767	2,843,263,701	2,841,991,360	2,851,115,509	2,847,893,417
	contig N50	25,894,807	25,692,063	24,108,925	13,125,096	4,226,428
	max. contig length	109,927,675	94,304,511	105,741,764	77,076,239	23,200,772
CSA final	total scaffold length	2,866,335,788	2,857,919,940	2,858,673,516	2,869,385,805	2,879,298,236
	total contig length	2,849,495,757	2,842,762,436	2,841,530,625	2,850,195,964	2,846,708,724
	placed in top n chr	97.50%	97.66%	97.96%	97.00%	96.83%
	scaffold N50	150,569,357	150,914,039	151,221,074	142,388,744	143,182,554
	contig N50	25,894,807	29,383,620	26,652,963	16,727,580	5,840,632
	max. scaffold length	233,785,065	235,400,774	233,997,714	235,967,303	236,759,292
	max. contig length	109,927,675	94,304,511	105,741,764	110,922,859	36,670,033
	runtime server (80 threads E7-8890v4@2.20GHz)	16:00h	11h:26m	10h:45	7h:49m	7h:04m
	contig N50 improvement over CSA step1 [x-fold]	1.66	1.80	2.13	3.82	4.49
	contig N50 impr. over best published SMRT assembly [x-fold]	0.98	1.12	1.01	0.64	0.22
Errors scf	fusions; intra-chr. translocations; inversions (blocks >300kbp)	f:0; t:6; i:2	f:0; t:14; i:1	f:0; t:6; i:0	f:2; t:14; i:2	f:4; t:7; i:1
Errors ctg		f:0; t:2; i:0	f:0; t:1; i:3	f:0; t:0; i:0	f:2; t:4; i:3	f:2; t:4; i:3

	vertebrate clade	Mammalia	Mammalia	Mammalia	Mammalia	Mammalia
CSA setup	species	*Homo sapiens*	*Homo sapiens*	*Homo sapiens*	*Homo sapiens*	*Homo sapiens*
	species haploid chr count=n	23	23	23	23	23
	input data type: seq. coverage; N50 read length	SMRT: 60-fold; N50:20 kb	SMRT: 40-fold; N50:20 kb	SMRT: 30-fold; N50:20 kb	SMRT: 20-fold; N50:20 kb	SMRT: 15-fold; N50:20 kb
	benchmark scenario	diverged ref	lower coverage / div.ref	lower coverage / div.ref	lower coverage / div.ref	lower coverage / div.ref
	reference	*P. abelii*	*P. abelii*	*P. abelii*	*P. abelii*	*P. abelii*
	reference divergence time	15.8	15.76	15.8	15.8	15.8
	reference haploid chr count	24	24	24	24	24
CSA step1	total contig length	2,846,783,372	2,840,640,553	2,841,463,516	2,847,406,287	2,826,278,103
	contig N50	15,637,873	16,341,163	12,497,249	4,383,406	1,299,535
	max. contig length	103,078,150	88,948,008	82,332,068	27,279,889	11,252,181
CSA step2	placed in top n chr	93.76%	95.17%	95.33%	94.29%	94.40%
	scaffold N50	128,353,462	130,971,511	131,070,274	131,625,032	132,314,504
	max. scaffold length	219,829,795	218,626,822	220,361,819	222,667,500	246,425,011
CSA step3	total contig length	2,849,302,170	2,843,047,974	2,841,357,350	2,850,745,354	2,846,782,940
	contig N50	25,506,235	25,690,650	19,077,358	12,527,629	4,187,987
	max. contig length	106,332,896	110,075,087	105,740,926	77,028,376	29,369,898
CSA final	total scaffold length	2,858,013,933	2,851,752,214	2,850,589,323	2,861,981,207	2,873,174,020
	total contig length	2,849,099,702	2,842,870,217	2,841,215,149	2,850,328,062	2,845,954,290
	placed in top n chr	93.84%	95.34%	95.45%	94.17%	94.52%
	scaffold N50	127,999,133	130,814,023	130,998,612	131,456,531	132,067,157
	contig N50	29,334,513	25,696,829	25,502,124	16,322,611	5,493,827
	max. scaffold length	219,825,495	218,621,187	219,807,659	222,045,937	244,800,533
	max. contig length	109,935,533	110,075,087	105,740,926	110,874,709	41,099,382
	runtime server (80 threads E7-8890v4@2.20GHz)	15h30m	15h:03m	13h:28m	12h:41m	12h:15m
	contig N50 improvement over CSA step1 [x-fold]	1.88	1.57	2.04	3.72	4.23
	contig N50 impr. over best published SMRT assembly [x-fold]	1.12	0.98	0.97	0.62	0.21
Errors scf	fusions; intra-chr. translocations; inversions (blocks >300kbp)	f:0; t:58; i:8	f:1; t:56; i:12	f:0; t:65; i:8	f:6; t:68; i:7	f:4; t:67; i:13
Errors ctg		f:0; t:4; i:6	f:0; t:5; i:11	f:0; t:8; i:7	f:4; t:8; i:8	f:2; t:6; i:6

Supplementary Table 5: *H. sapiens* CSA assembly by ultra-long reads.

	**vertebrate clade**	Mammalia	Mammalia
CSA setup	**species**	*Homo sapiens*	*Homo sapiens*
	**species haploid chr count=n**	23	23
	**input data type: seq. coverage; N50 read length**	ONT: 50-fold; N50:70 kb	ONT: 50-fold; N50:70 kb
	**benchmark scenario**	best case	diverged reference
	**reference**	*H. sapiens*	*P. abelii*
	**reference divergence time**	0,0	15,8
	**reference haploid chr count**	23	24
CSA step1	**total contig length**	2.890.906.026	2.890.906.026
	**contig N50**	38.151.541	38.151.541
	**max. contig length**	109.306.044	109.306.044
CSA step2	**placed in top n chr**	96,58%	91,20%
	**scaffold N50**	150.526.033	126.341.499
	**max. scaffold length**	235.656.620	220.727.909
CSA step3	**total contig length**	2.883.898.281	2.884.552.645
	**contig N50**	39.712.896	39.524.723
	**max. contig length**	109.194.274	109.206.036
CSA final	**total scaffold length**	2.891.827.299	2.888.829.127
	**total contig length**	2.883.411.943	2.884.341.430
	**placed in top n chr**	96,69%	92,90%
	**scaffold N50**	150.158.625	128.460.016
	**contig N50**	48.445.356	45.943.940
	**max. scaffold length**	236.106.895	219.961.958
	**max. contig length**	109.194.274	118.964.814
	**runtime server (80 threads E7-8890v4@2.20GHz)**	23h:43m	
	**contig N50 improvement over CSA step1 [x-fold]**	1,27	1,20
	**contig N50 impr. over best published SMRT assembly [x-fold]**	1,84	1,75
Errors scf	**fusions; intra-chr. translocations; inversions (blocks >300kbp)**	f:0; t:9; i:2	f:2; t:53; i:8
Errors ctg		f:0; t:0; i:2	f:1; t:5; i:6

Supplementary figures:

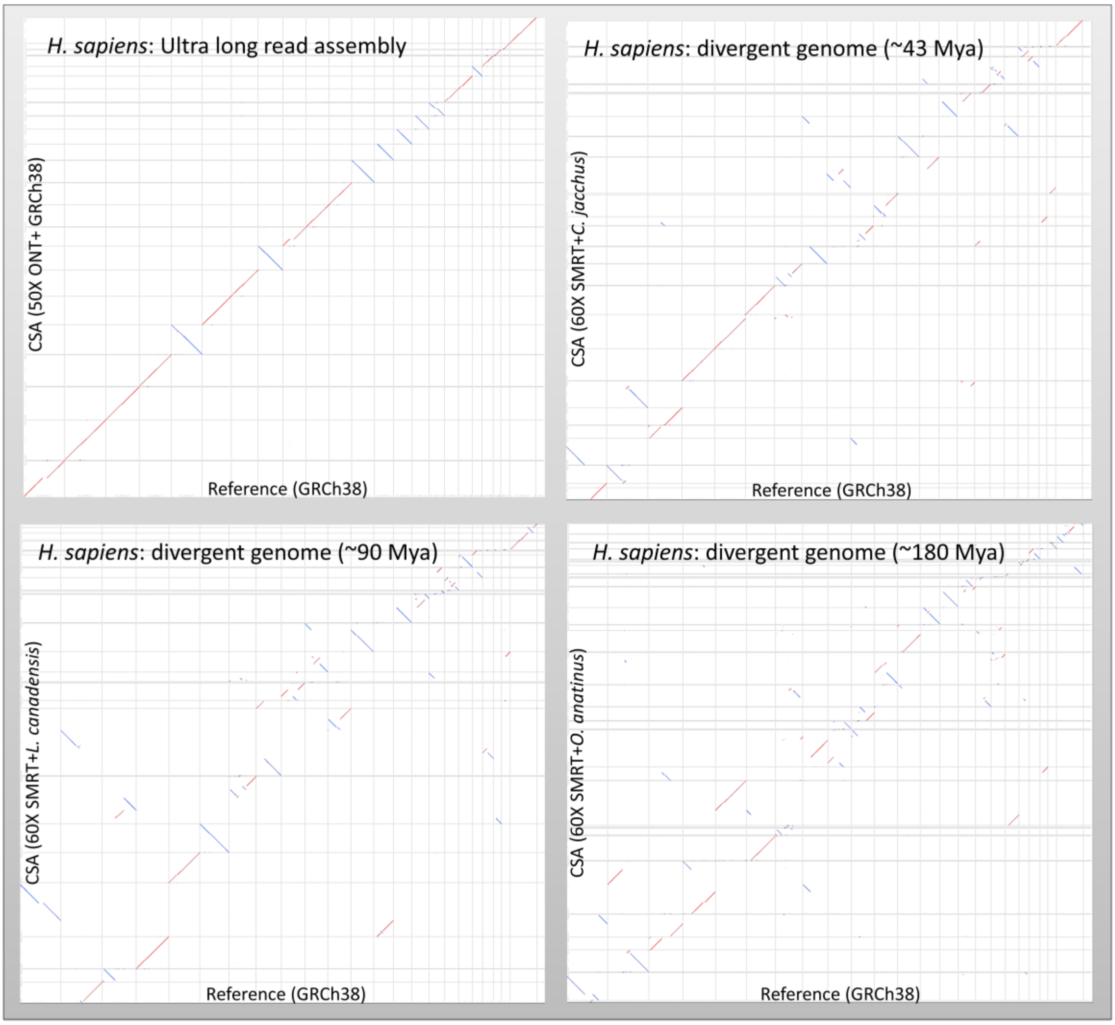

Supplementary Figure 1: Additional dot plots for *H. sapiens* CSA assemblies using ONT ultra long reads, or SMRT reads and more diverged reference genomes.

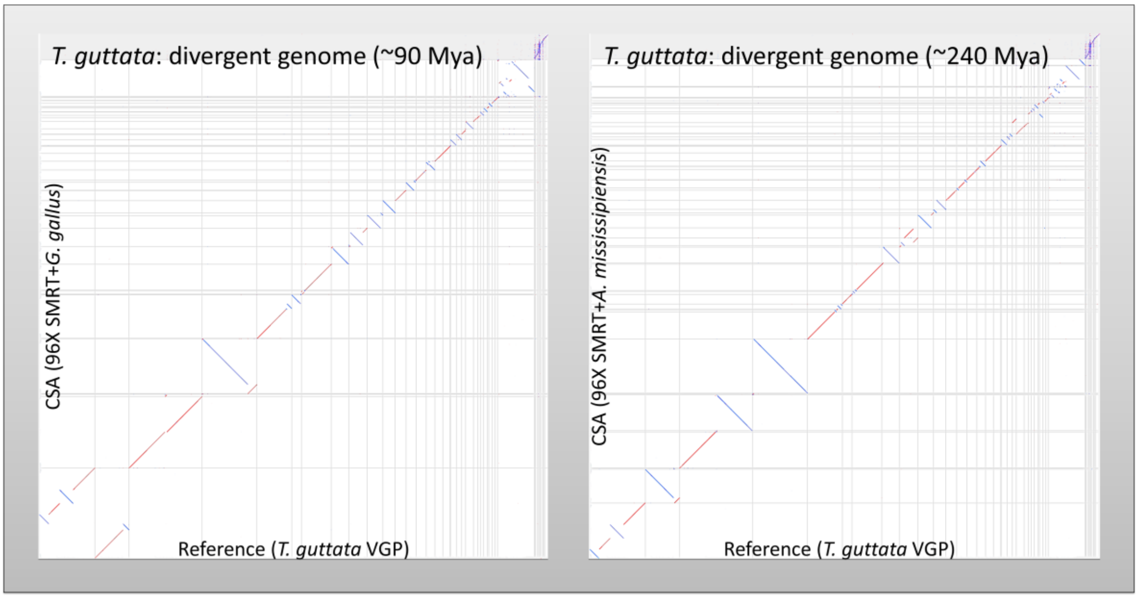

Supplementary Figure 2: Additional dot plots for *T. guttata* CSA assemblies using SMRT reads and more diverged reference genomes.

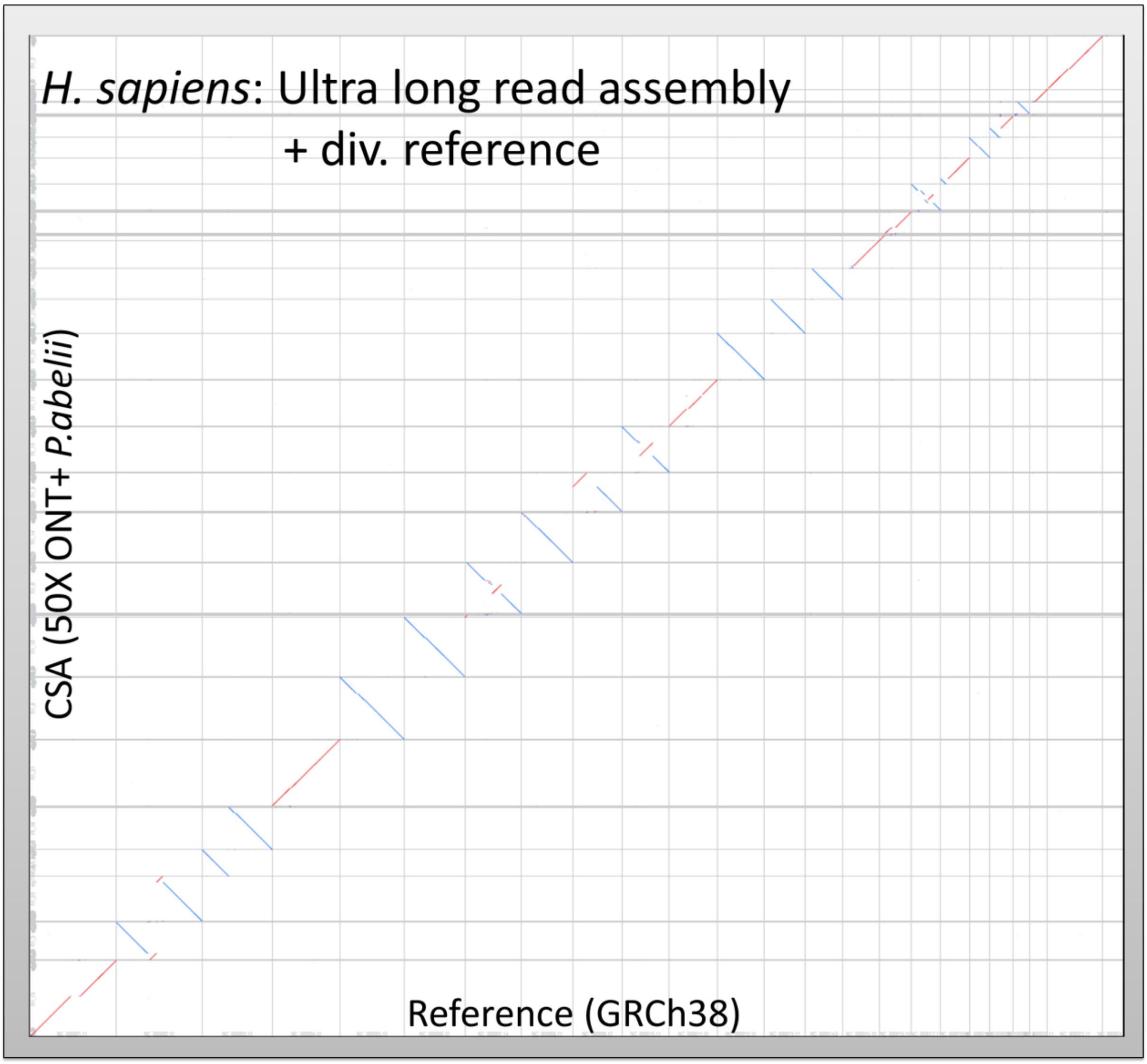

Supplementary Figure 3: Additional dot plot for *H. sapiens* CSA assembly using ONT ultra long reads and a diverged reference genome (*P. abelii*).

Additional file 3 + 4 download site

Click here to access/download
Supplementary Material
CSA2.6-DOWNLOAD.pdf

Click here to access/download
Supplementary Material
CSA-Eval table_version7.xlsx

Click here to access/download
Supplementary Material
CSA-Figures_HiRes_R1.pptx
