# Supplementary material for: CSA: A high-throughput chromosome-scale assembly pipeline for vertebrate genomes
Source: Gigascience. 2020 May 25;9(5):giaa034. doi: 10.1093/gigascience/giaa034 (PMC7247394; doi:10.1093/gigascience/giaa034)
Supplement: giaa034_Supplemental_Files [file giaa034_supplemental_files.zip › CSA-Figures_HiRes_R2.pptx]

## Slide 1
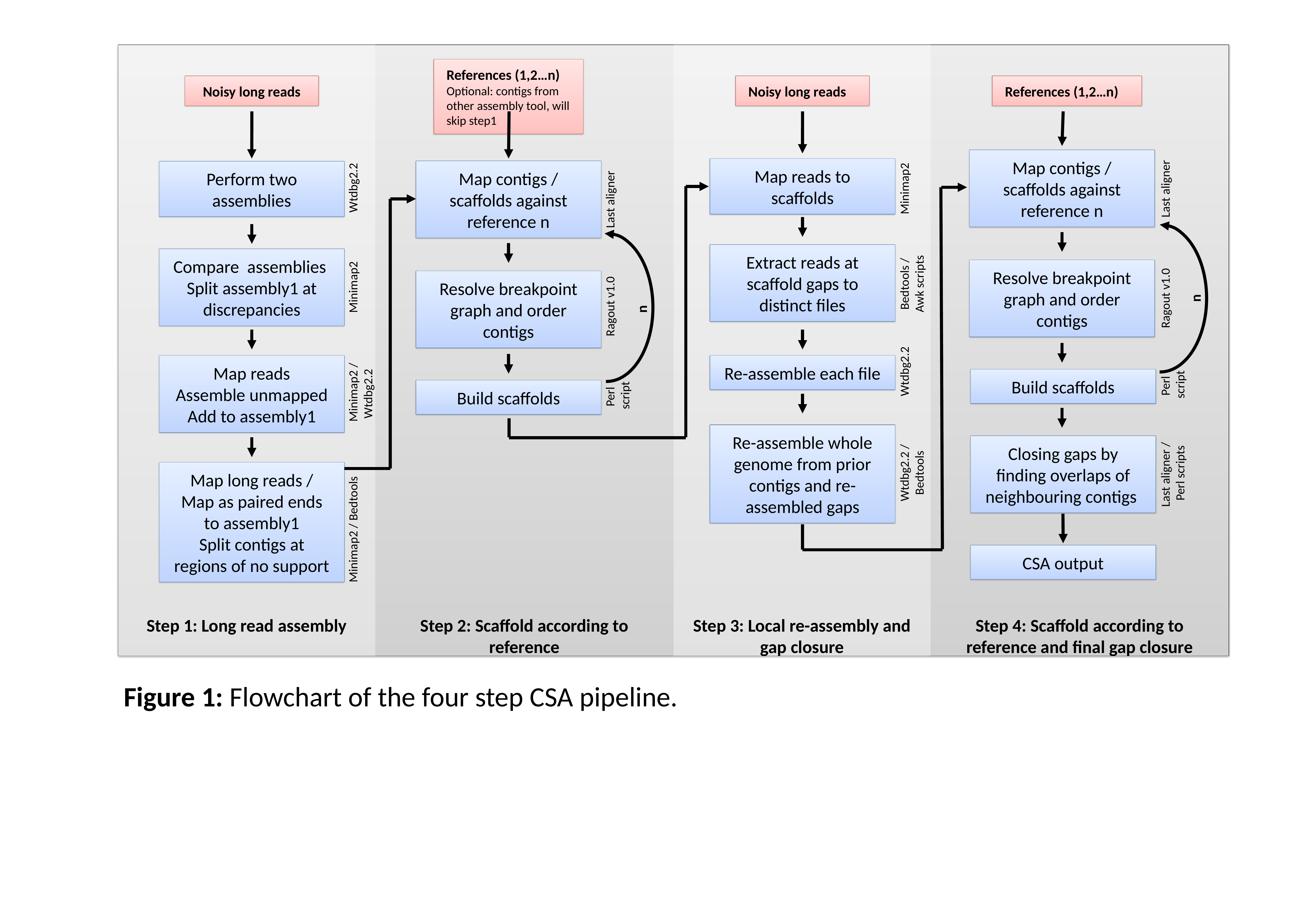

References (1,2…n)
Optional: contigs from other assembly tool, will skip step1
Noisy long reads
Noisy long reads
References (1,2…n)
Map contigs / scaffolds against reference n
Map reads to scaffolds
Map contigs / scaffolds against reference n
Perform two assemblies
Minimap2
Wtdbg2.2
Last aligner
Last aligner
Extract reads at scaffold gaps to distinct files
Compare assemblies
Split assembly1 at discrepancies
Resolve breakpoint graph and order contigs
Bedtools / Awk scripts
Resolve breakpoint graph and order contigs
Minimap2
n
Ragout v1.0
n
Ragout v1.0
Re-assemble each file
Map reads
Assemble unmapped
Add to assembly1
Wtdbg2.2
Perl
 script
Build scaffolds
Minimap2 / Wtdbg2.2
Perl
 script
Build scaffolds
Re-assemble whole genome from prior contigs and re-assembled gaps
Closing gaps by finding overlaps of neighbouring contigs
Wtdbg2.2 / Bedtools
Last aligner / Perl scripts
Map long reads /
Map as paired ends
to assembly1
Split contigs at regions of no support
Minimap2 / Bedtools
CSA output
Step 2: Scaffold according to reference
Step 3: Local re-assembly and gap closure
Step 4: Scaffold according to reference and final gap closure
Step 1: Long read assembly
Figure 1: Flowchart of the four step CSA pipeline.

## Slide 2
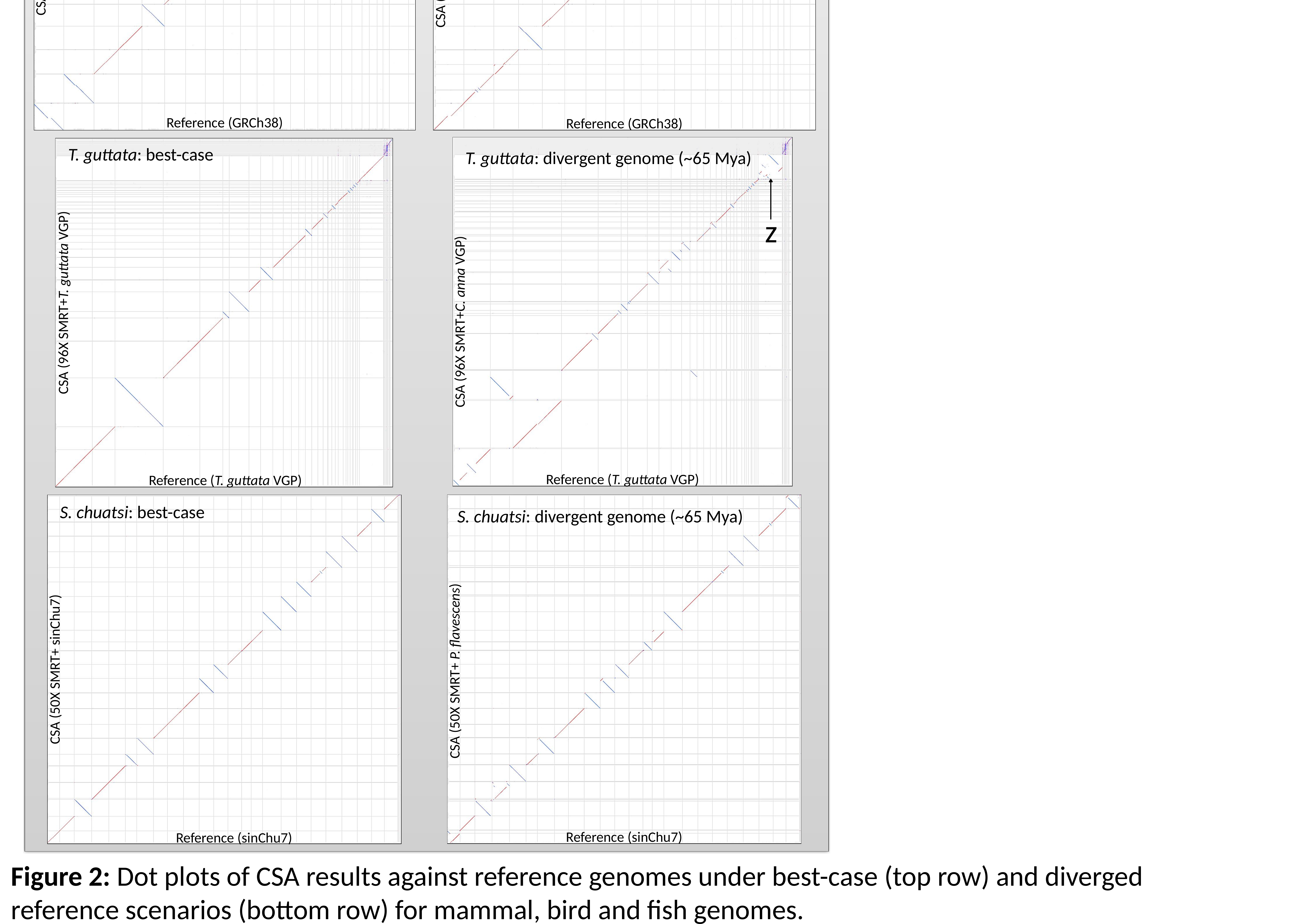

H. sapiens: best-case
CSA (60X SMRT+GRCh38)
Reference (GRCh38)
H. sapiens: divergent genome (~15 Mya)
CSA (60X SMRT+P. abelii)
Reference (GRCh38)
x
T. guttata: best-case
CSA (96X SMRT+T. guttata VGP)
Reference (T. guttata VGP)
T. guttata: divergent genome (~65 Mya)
CSA (96X SMRT+C. anna VGP)
Reference (T. guttata VGP)
z
S. chuatsi: best-case
CSA (50X SMRT+ sinChu7)
Reference (sinChu7)
S. chuatsi: divergent genome (~65 Mya)
CSA (50X SMRT+ P. flavescens)
Reference (sinChu7)
Figure 2: Dot plots of CSA results against reference genomes under best-case (top row) and diverged reference scenarios (bottom row) for mammal, bird and fish genomes.

## Slide 3
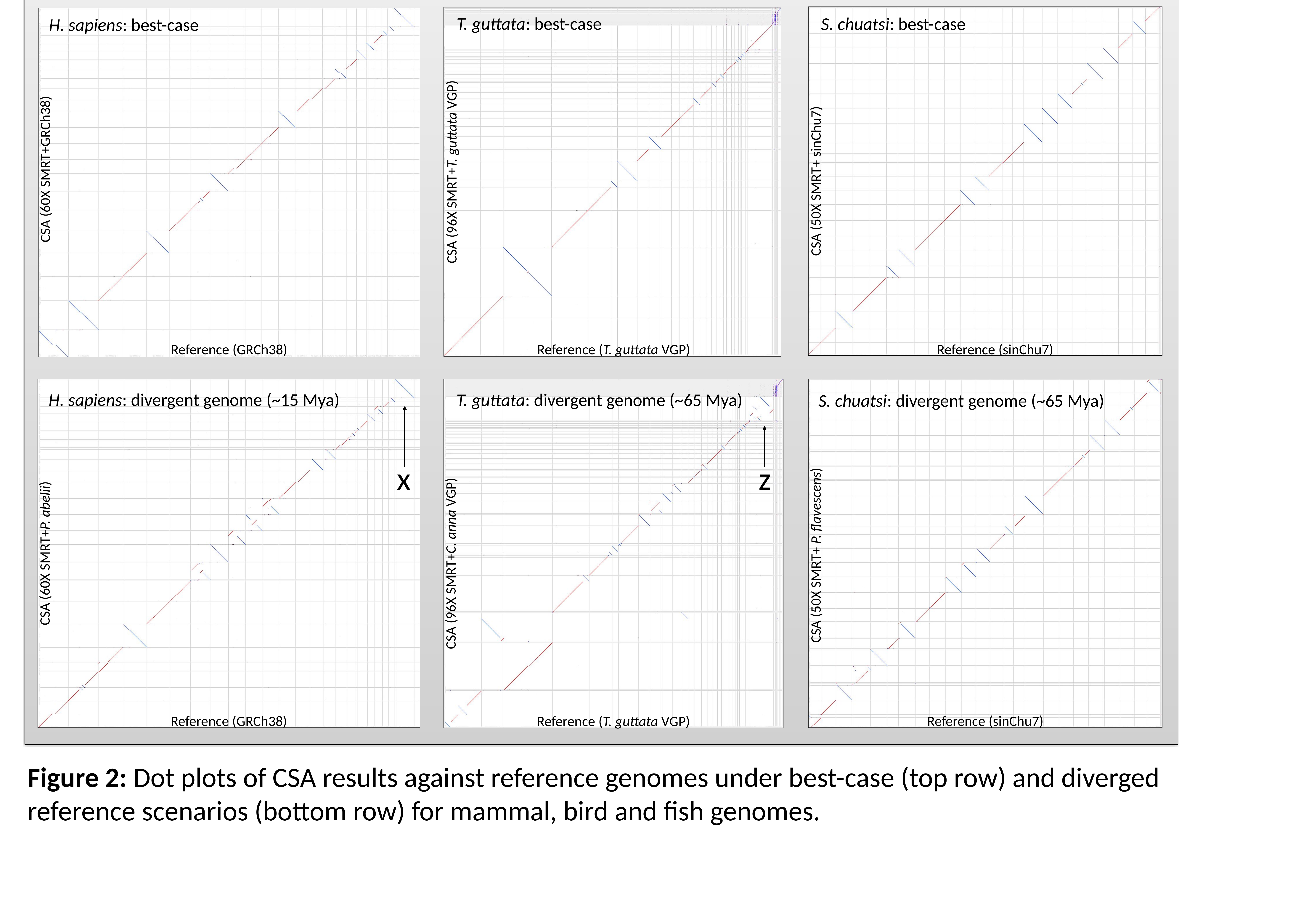

T. guttata: best-case
CSA (96X SMRT+T. guttata VGP)
Reference (T. guttata VGP)
S. chuatsi: best-case
CSA (50X SMRT+ sinChu7)
Reference (sinChu7)
H. sapiens: best-case
CSA (60X SMRT+GRCh38)
Reference (GRCh38)
H. sapiens: divergent genome (~15 Mya)
CSA (60X SMRT+P. abelii)
Reference (GRCh38)
S. chuatsi: divergent genome (~65 Mya)
CSA (50X SMRT+ P. flavescens)
Reference (sinChu7)
T. guttata: divergent genome (~65 Mya)
CSA (96X SMRT+C. anna VGP)
Reference (T. guttata VGP)
Figure 2: Dot plots of CSA results against reference genomes under best-case (top row) and diverged reference scenarios (bottom row) for mammal, bird and fish genomes.
x
z

## Slide 4
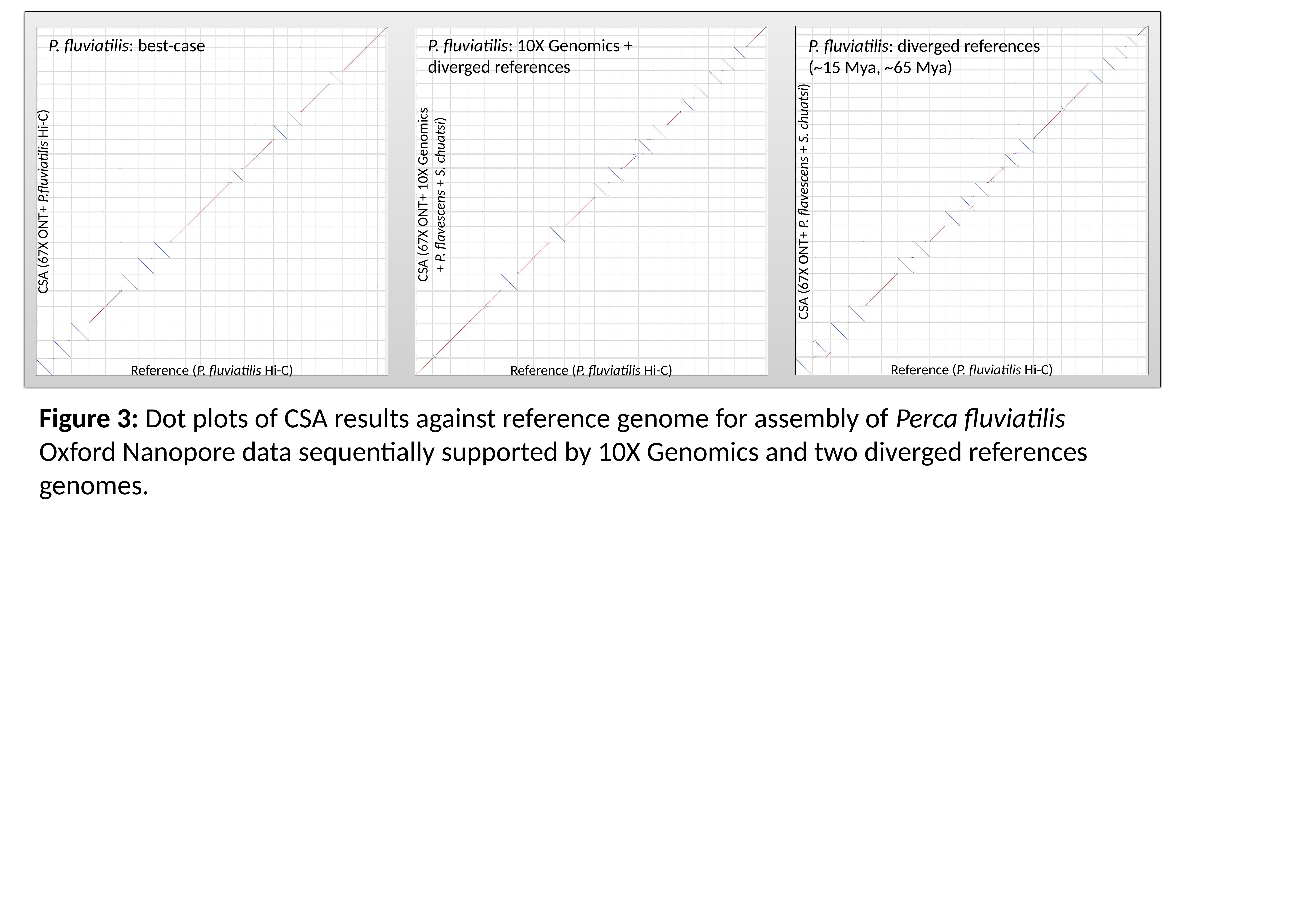

P. fluviatilis: diverged references (~15 Mya, ~65 Mya)
CSA (67X ONT+ P. flavescens + S. chuatsi)
Reference (P. fluviatilis Hi-C)
P. fluviatilis: 10X Genomics + diverged references
CSA (67X ONT+ 10X Genomics + P. flavescens + S. chuatsi)
Reference (P. fluviatilis Hi-C)
P. fluviatilis: best-case
CSA (67X ONT+ P.fluviatilis Hi-C)
Reference (P. fluviatilis Hi-C)
Figure 3: Dot plots of CSA results against reference genome for assembly of Perca fluviatilis Oxford Nanopore data sequentially supported by 10X Genomics and two diverged references genomes.

## Slide 5
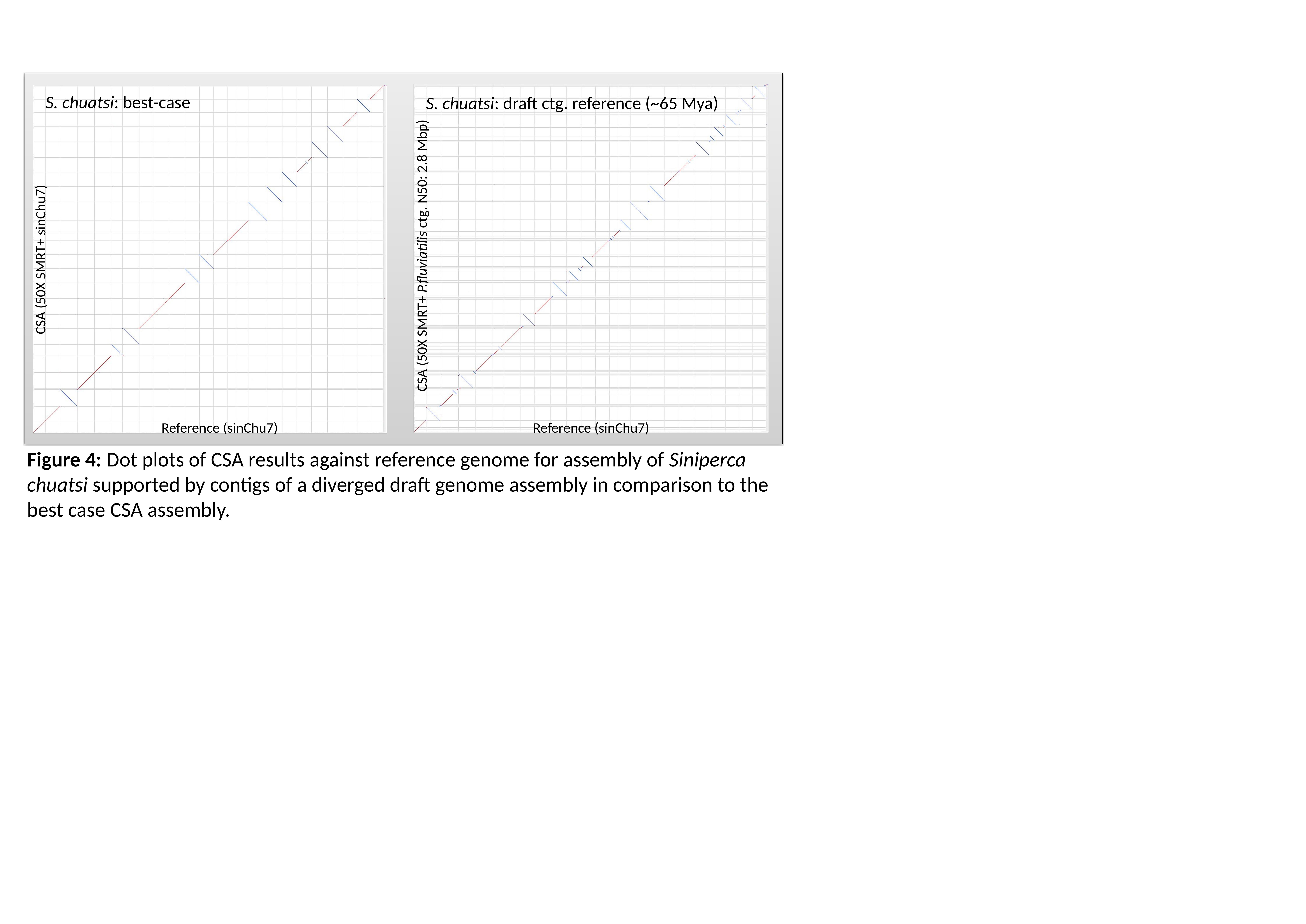

S. chuatsi: draft ctg. reference (~65 Mya)
CSA (50X SMRT+ P.fluviatilis ctg. N50: 2.8 Mbp)
Reference (sinChu7)
S. chuatsi: best-case
CSA (50X SMRT+ sinChu7)
Reference (sinChu7)
Figure 4: Dot plots of CSA results against reference genome for assembly of Siniperca chuatsi supported by contigs of a diverged draft genome assembly in comparison to the best case CSA assembly.

## Slide 6
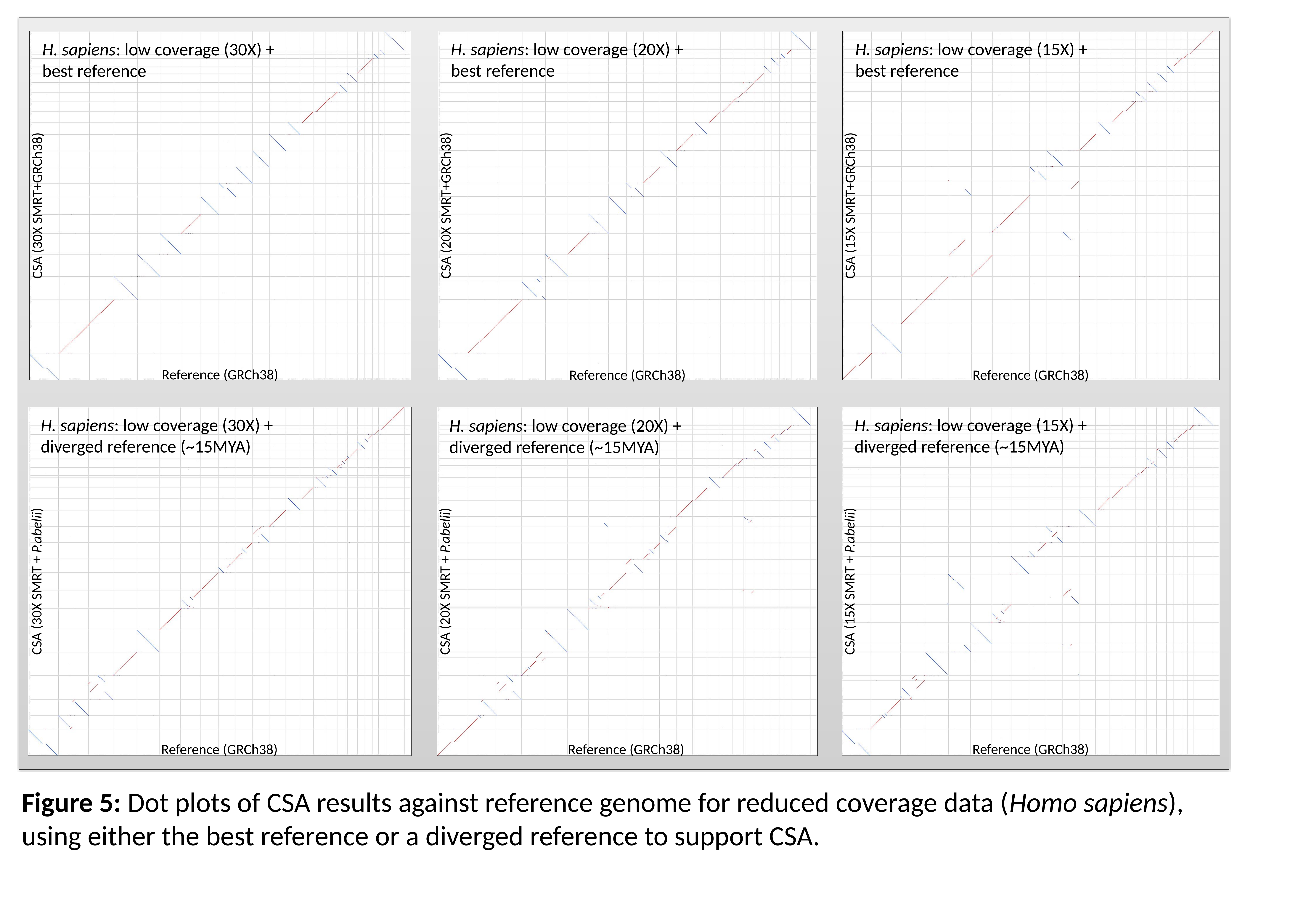

H. sapiens: low coverage (15X) +
best reference
CSA (15X SMRT+GRCh38)
Reference (GRCh38)
H. sapiens: low coverage (20X) +
best reference
CSA (20X SMRT+GRCh38)
Reference (GRCh38)
H. sapiens: low coverage (30X) +
best reference
CSA (30X SMRT+GRCh38)
Reference (GRCh38)
H. sapiens: low coverage (20X) + diverged reference (~15MYA)
CSA (20X SMRT + P.abelii)
Reference (GRCh38)
H. sapiens: low coverage (15X) + diverged reference (~15MYA)
Reference (GRCh38)
CSA (15X SMRT + P.abelii)
H. sapiens: low coverage (30X) + diverged reference (~15MYA)
CSA (30X SMRT + P.abelii)
Reference (GRCh38)
Figure 5: Dot plots of CSA results against reference genome for reduced coverage data (Homo sapiens), using either the best reference or a diverged reference to support CSA.

## Slide 7
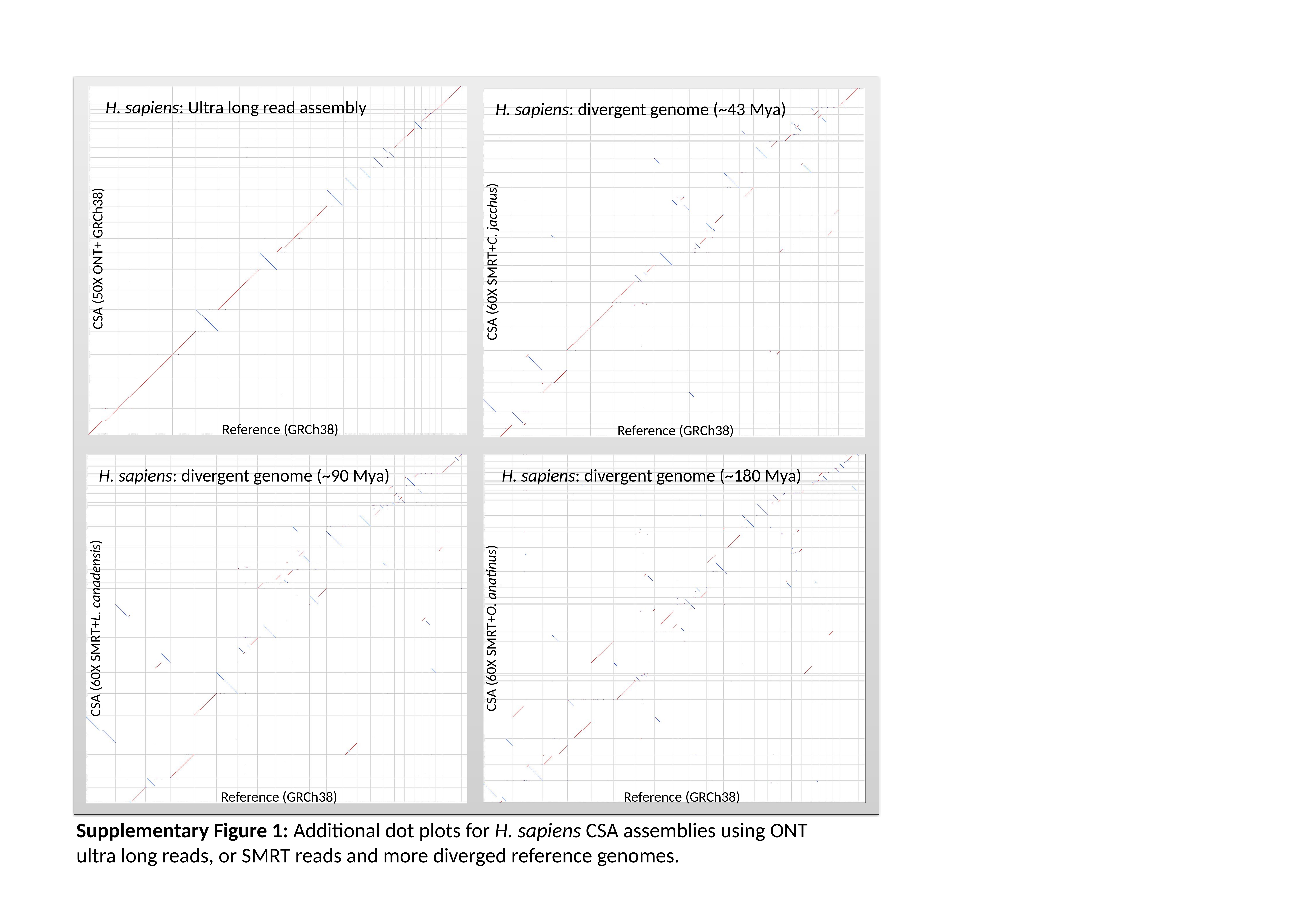

H. sapiens: Ultra long read assembly
CSA (50X ONT+ GRCh38)
Reference (GRCh38)
H. sapiens: divergent genome (~43 Mya)
CSA (60X SMRT+C. jacchus)
Reference (GRCh38)
H. sapiens: divergent genome (~180 Mya)
CSA (60X SMRT+O. anatinus)
Reference (GRCh38)
H. sapiens: divergent genome (~90 Mya)
CSA (60X SMRT+L. canadensis)
Reference (GRCh38)
Supplementary Figure 1: Additional dot plots for H. sapiens CSA assemblies using ONT ultra long reads, or SMRT reads and more diverged reference genomes.

## Slide 8
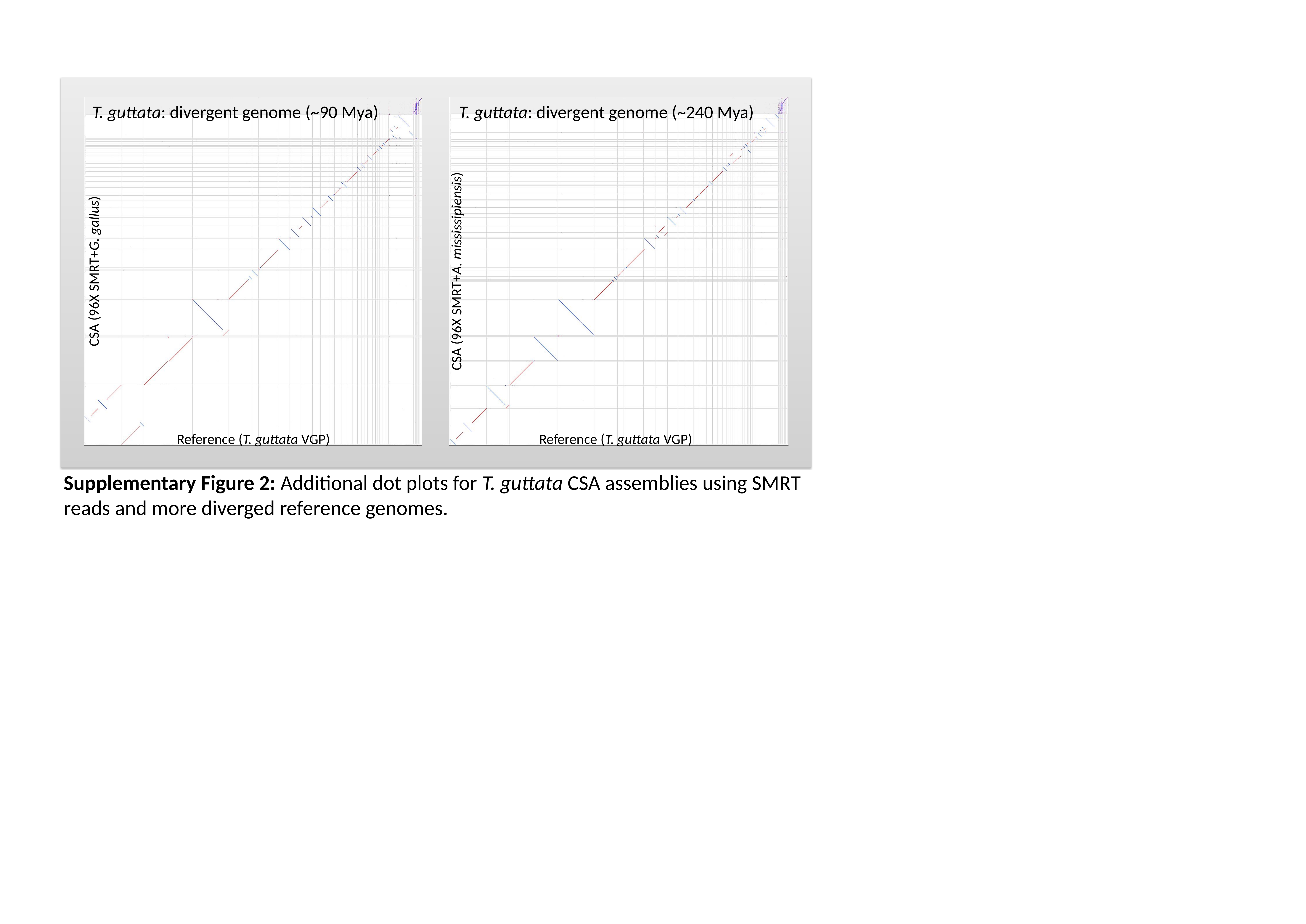

T. guttata: divergent genome (~90 Mya)
CSA (96X SMRT+G. gallus)
Reference (T. guttata VGP)
T. guttata: divergent genome (~240 Mya)
CSA (96X SMRT+A. mississipiensis)
Reference (T. guttata VGP)
Supplementary Figure 2: Additional dot plots for T. guttata CSA assemblies using SMRT reads and more diverged reference genomes.

## Slide 9
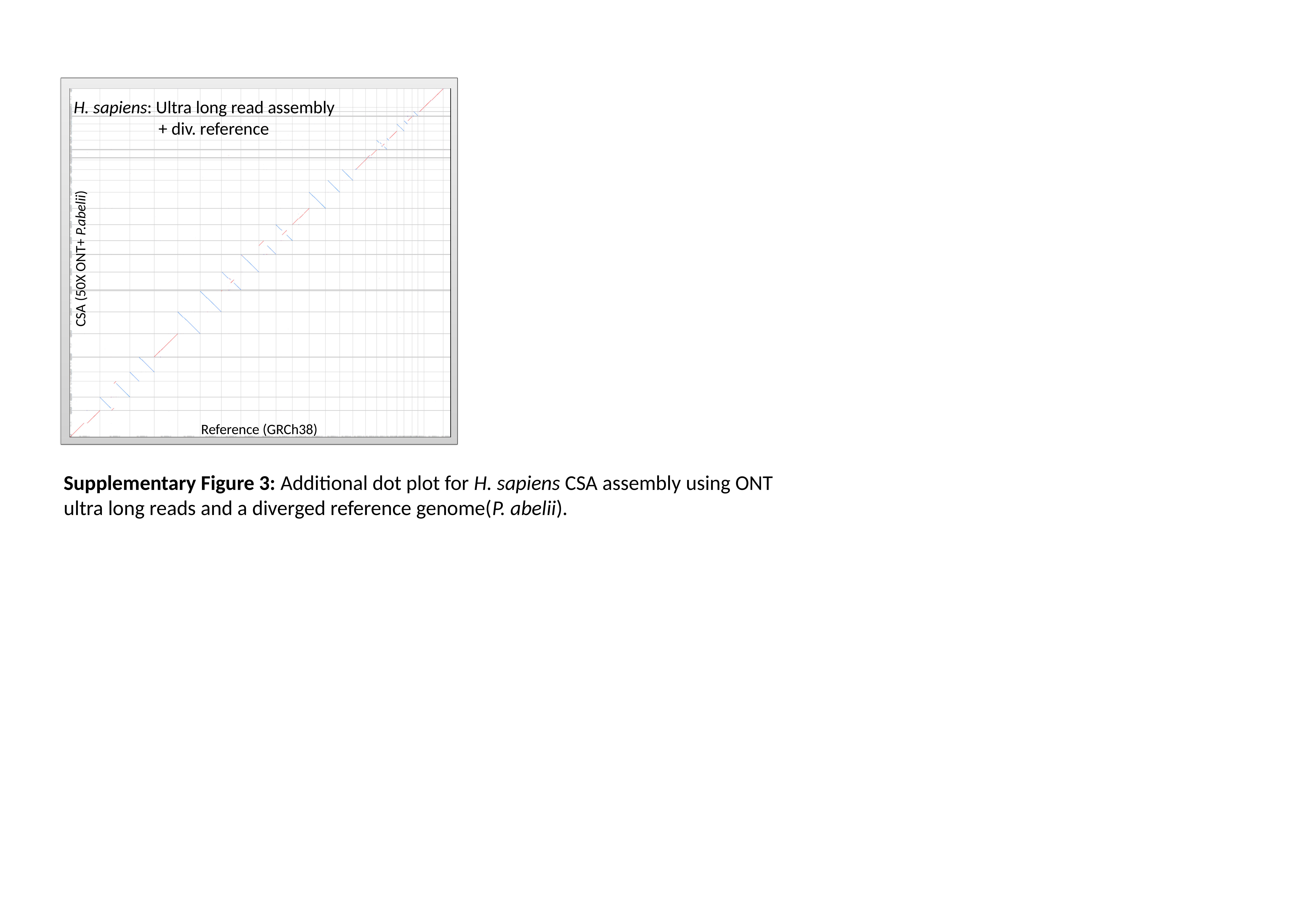

H. sapiens: Ultra long read assembly
 + div. reference
CSA (50X ONT+ P.abelii)
Reference (GRCh38)
Supplementary Figure 3: Additional dot plot for H. sapiens CSA assembly using ONT ultra long reads and a diverged reference genome(P. abelii).
