# Supplementary material for: CSA: A high-throughput chromosome-scale assembly pipeline for vertebrate genomes
Source: Gigascience. 2020 May 25;9(5):giaa034. doi: 10.1093/gigascience/giaa034 (PMC7247394; doi:10.1093/gigascience/giaa034)
Supplement: giaa034_Supplemental_Files [file giaa034_supplemental_files.zip › CSA2.6-DOWNLOAD.pdf]

CSA2.6 pipeline download for reviewers:

https://nimbus.igb-berlin.de/index.php/s/iZDrswbpEmsi9PB

passw: CSA-MBE2019
