# Supplementary material for: CSA: A high-throughput chromosome-scale assembly pipeline for vertebrate genomes
Source: Gigascience. 2020 May 25;9(5):giaa034. doi: 10.1093/gigascience/giaa034 (PMC7247394; doi:10.1093/gigascience/giaa034)
Supplement: giaa034_Response_to_Reviewer_Comments_Original_Submission [file giaa034_response_to_reviewer_comments_original_submission.pdf]

Author's Response To Reviewer Comments

Close

Dear Dr. Edmunds,

we herewith submit our revised manuscript "CSA: A high-throughput chromosome-scale assembly pipeline for vertebrate genomes" for your consideration.
We tried to answer all open questions and changed the manuscript accordingly. In this regard, we would like to thank the reviewers and editor for their valuable comments.
As requested CSA has now been registered to "bio.tools" and "SciCrunch.org" databases.

Regarding the benchmarking of CSA, we have discussed other options among the authors but would like to stay with comparing results of CSA to state-of-the-art reference genome assemblies, which represent the best assemblies currently available for the different species presented in the manuscript.

We would like to do so for mainly three reasons:

a) There is currently no pipeline for long-read assembly published that is comparable to CSA. Similar pipelines are only available for short-read assembly (e.g. IMAP proposed by Reviewer 1).

b) CSA is a bioinformatics pipeline that automates many steps (4 to 5 different tasks) necessary to arrive at chromosomal-scale assemblies. For each of these tasks many different software tools are available and testing all kinds of combinations of these tools in comparison to CSA would be out of scope of the manuscript and very puzzling to the reader.

c) It would be easy to choose a set of programs which chained would perform less well than CSA and such a benchmark would be meaningless, while comparison with reference genomes is much more reliable.
We are convinced that our benchmarking system clearly shows that CSA produces results that are very close to state-of-the-art reference genomes and at the same time reduces costs for input data types, computational resources and manual curation time.

We hope you may consider our manuscript to be published in Gigascience as a Technical Note.

Yours sincerely,

Dr. Heiner Kuhl
On behalf of all authors

The authors' answers to reviewer reports are marked by "R:"

Reviewer #1: The paper presents a new pipeline for genome assembly. This pipeline uses reference genome, gap filling and assembly correction, for the production of the final assembly. The pipeline includes tools with a low memory footprint (relatively to genome assembly tools).
The paper is clearly written, and 6 use cases, which provide metrics on several scenarios, are presented.
This work should be useful for many lab around the world.
I have tried the example available on the GitHub repository. However, due to limited time to review this article (2 weeks), I did not have time to redo the analyses done in the article.
The package is easy to install and I could run seamlessly the small example.
However, I have major comments on the manuscript.
I feel that a description of the state of the art is missing.
When reading the paper, the user may wonder whether:

1. the pipeline deliver better results that other methods,

R:

We understand that the editor and both reviewers would like to see comparisons to existing pipelines that do a similar job as CSA. However, to our knowledge, if such pipelines do already exist, they have not been published. Reviewer 1 proposed IMAP, but we found that this pipeline was created for short read assembly, while CSA was created for long read assembly.
We could, in principle, combine other existing tools that solve parts of the chromosome-level assembly problem for comparisons (see table below), but this would actually mean constructing another new pipeline from scratch. If understood like this, there would be many combinations of several kinds of tools… However, we strongly believe that benchmarking would be out of scope (as reviewer 1, already mentioned).

To solve these issues, we have compared CSA results to published current state-of-the-art reference genome assemblies (horizontal axis of each dot plot / contig N50 improvements mentioned in text and suppl. tables), which have in most cases been obtained from many different sequencing and mapping techniques and substantial manual curation efforts. This type of benchmarking shows that CSA can deliver similar or even better results (regarding contig N50) with much less costs, efforts and at high-throughput level.

We now do explain our choice of benchmarking in the manuscript as follows (L194 – L199):
"In the following, we tested CSA on different scenarios and benchmark its performance. CSA automatically chains many steps that traditionally require different software tools and laborious manual curation, for which similar pipelines are currently only available for short-read assembly (IMAP[44]). Therefore, we do not compare CSA-results with known contig-level genome assembly tools or other software packages solving only parts of chromosomal assemblies, but by re-assembling the currently best chromosomal-scale reference genomes in different vertebrate species."

To underline our benchmarking we have now added Table 1 to the manuscript, which directly compares important statistical values of the state-of-the-art reference genomes and "CSA + diverged genome" assemblies.

2. the selected features (gap filling, assembly correction, etc.) are the best mix (i.e. why did you choose these features over others?),

R:
We now have improved this and discuss our choices in the manuscript (see below for details regarding the different alternative tools that the reviewers proposed).

3. the selected tools (implementing the features) have been carefully selected.

R:
As above, we explain our choices now (see below).

Objective 1 has been partly addressed. However, I think that the other objectives deserve at least a discussion (an exhaustive benchmark may be out of scope).

Main questions:
- Concerning objective 1:
+ Please compare your approach/results with IMAP.

R: Thanks for hinting us at IMAP, which is a pipeline with similar features as CSA but it can only handle short reads. To address this point, we now cite IMAP in the Introduction (L117-L118) and mention it as being similar to CSA (L196 - L197). Unfortunately, IMAP it is not able to use long read data and thus cannot be directly compared with CSA.

- Concerning objective 2:
+ Why did you not include other tools, such as polishers? You mentionned MEDAKA and PILON in scenario 3. If you did not include them in the pipeline, please mention why.

R:
The reviewer is right; consensus polishing is a crucial step after assembling noisy long read data. A variety of methods are available. We think an in-depth analysis of consensus polishing methods would be a manuscript of its own and we would like to focus on chromosomal-level assembly in our manuscript. Nevertheless, we do now state more clearly that CSA currently does not perform polishing

(L191 - L193):
“The current version of CSA does not include methods for consensus polishing, yet, one iteration of consensus polishing using long-reads and two iterations using short-reads should be applied prior to sequence annotation efforts.”

- Concerning objective 3:
+ Please discuss the use of RAGOUT (by the way, why not RAGOUT 2?), compared to other tools, such as MeDuSa or RACA.

R:
We have long experience using RAGOUT1 for constructing chromosomal-scale assemblies using related reference genomes (see for example burrowing owl genome; PMID: 29769357). We have also tested RAGOUT2 within CSA, but found that it produced slightly less complete chromosomal reconstructions (few percent) in this setting. RAGOUT2 by default also breaks assemblies and fits them to the reference, although this may be helping to remove missassemblies when using closely related reference genomes, it may also remove true rearrangements between genomes, thus we decided to stay with RAGOUT1. RAGOUT1 or 2 support maf alignment format, which made it easy to combine it with the fast and at the same time sensitive LAST aligner. We now briefly discuss this issue in the new version of the manuscript.

We are convinced that we cannot use MeDuSa, as it relies on Mummer, which is not a sensitive and fast aligner for whole genome alignment of large, diverged genomes.

Likewise, we do not use RACA, because it is a very complex pipeline consisting of many scripts. It is not able to use multiple references in parallel and it is by default using slower alignment methods (here „LASTZ“, while CSA uses „LAST aligner“, a completely different tool despite similar name). Again, we now briefly discuss this point in the new version of the manuscript.

Taken together, we mention these tools now in the section “Implementation of the CSA pipeline” L180 - L183 and explain why we think it is more appropriate to stay with RAGOUT1.

+ Some tools have been developed for CSA: breaking contigs, joining contigs, closing gaps. Why did you implement these features, whereas solutions already exist: SMSC or BIGMAC for breaking contigs, LINKS for scaffolding, LR_Gapcloser or GAPPader for closing gaps? These tools have been published and benchmarked (to some extent). Do your tools perform better?

R:
SMSC: Has not been tested on vertebrate genomes, just E. coli and Yeast. Possibly performance issues may exist for vertebrate-sized genomes (as it is using outdated aligners like NUCMER or BLASR by default).
BIGMAC: this has been designed for metagenomes but not for genomes, running 2 h with 20 threads on small assemblies. BIGMAC on large genomes would possibly take longer than running the whole CSA pipeline. To address this, we do now discuss assembly reconciliation in L171 - L179.

LINKS: We have tested LINKS a while ago, but were not happy with its performance on large genomes. The alignment-free kmer analysis implemented in LINKS cannot be parallelized and is actually much slower than current multi-threaded mapping tools like minimap2 on large datasets.

LR_GAPCLOSER and also PBJelly: in many cases appears inefficient, as long neighboring contig overlaps remain unclosed, such unresolved repeats are typical for long read assemblies.
In response, we discuss this now in L188 - L191.

GAPPADER: This tool uses paired-end or mate-pair short reads for gap closure, it cannot be used for gap closure by long reads.

Other questions:
- Did you consider using Nextflow or Snakemake? This would probably help the users who work on a cluster for an easier interface with a job scheduler.

R:
We thank the reviewer for this suggestion and have discussed this among the authors. CSA, as most

current long-read assembly tools, depends on a single computing node with high amount of RAM and CPU power, running jobs distributed on a cluster would not result in much better performance as there is little room for parallelization of the pipeline. This lowers our interest of using a job scheduler system. Anyway, we will consider adapting the pipeline to improve usability (e.g. easy re-start of stopped processes etc.) in future versions.

- I do not exactly understand why, on Scenario 5, the assembly is not perfect, even with low coverage, given that the very same genome is given as reference (as far as I understand it). Why is not LAST+RAGOUT stitching contigs?

R:
The assembly is imperfect, because with lower coverage more miss-assemblies occur in the primary assembly and slip through the miss-assembly correction. CSA does not split the primary contigs according to the reference, because otherwise species-specific rearrangements would be removed from the assemblies.

The reviewer is right that RAGOUT can close gaps, but RAGOUT was designed to close gaps based on exact sequence matches between neighboring contig ends, as these occur in de bruijn graph short-read assemblies. With long-read read assemblies, neighboring contig overlaps are typically noisy (often even partly uncorrected reads), thus RAGOUT cannot close these gaps.
This is now explained in the main text (L184 - L187)

- Scenario 6, do you compare ULR+CSA+GRCh38 to ULR+SHASTA? If so, since you provide the "true" genome, is not the first method highly favored?

R:
This benchmark was mainly to show that wtdbg2 and thus CSA can be used to assemble ULRs, which was questioned by the SHASTA manuscript. We now do also provide information on an ULR assembly using not the “true” genome, but a diverged reference genome(L413 - L416).

- Could you please provide "meta-paramaters" to your tool? In minimap2, the author use the "-x" paramter. You could adopt something similar to handle Sequel reads.

R:
In the newest version of CSA parameter –I can be used to parametrize WTDBG2 in more detail, we give examples for sequel reads or ULRs in the Readme file. A preset for different input data will be implemented in future versions.

Minor questions and comments:
- Page 3, there is probably something missing in the sentence: "[...] huge genome sizes of amphibians pose strong exceptions: c-value [...]"

R:
We changed this to: "[...] in this regard the huge genome sizes of amphibians pose strong exceptions: c-value [...]"

- Page 3, what are "bird chromosomes"?

R:
To be clearer we have now changed this to:
“Nevertheless, ortholog genes that exhibit distinct order in bird chromosomes are also discretely ordered in the single well-assembled urodelan (Ambystoma mexicanum)”

- Figure 1, the tool names written in the right hand of the rectangles are too small. Please enlarge.

R:
We have doubled the font size.

- Figure 1, some rectangle texts start with a capital letter, some without. Why?

R:
We have unified the capital letters in the figure

- Figure 2, please present the figures in the caption. The reader may not be familiar with minidot outputs. Please explain the meaning of:
+ the vertical and horizontal thin lines,
+ the color of the diagonal lines.

R:
We now explain the plot properties in the caption of figure 2.

- Figure 2, please enlarge the dot plots, with at most two plots per row, preferably one.

R:
We have now adjusted the Figure 2 into two plots per row, which results in a full-page figure. All other options would take too much space. We think the full-sized plots could be better viewed downloading the supplement.

- Page 10, the commas seem misplaced in "Overall the fraction of consensus sequences assigned to the top n scaffolds, was slightly lower than [...]"

R:
We have removed the commas.

- Scenario 3, please briefly present the data used in the "10X Genomics assembly," such as sequencing technology and coverage.

R:
We added some basic values next to the citation: "….(68X Illumina short-read coverage, N50 contig / scaffold length 18.3 kbp / 6.3 Mbp according to [58])."

- Page 12, the comma seems superfluous in "To test, if CSA could reach [...]"

R:
We removed the comma.

- Page 12, please explain "C: 95.9%, F: 2.1%, M: 2.0%, n: 4584"

R:
We replaced the BUSCO shortcuts by full description:
"(Actinopterygii dataset, complete genes: 95.9%, fragmented genes: 2.1%, missing genes: 2.0%, number of tested genes: 4584)"

- Page 13, I do not understand the comma in "Benchmark scenario 4: CSA using draft assemblies as reference, contig-level assemblies of diverged species may be highly complementary." It is a semi-colon?

R:
As suggested we replaced the comma by a semicolon

- Other acronyms:
+ interchr.: inter-chromosomal
+ intrachr.: intra-chromosomal
+ f: inter-chromosomal fusion
+ t: intra-chromosomal translocation
+ i: inversion
+ Hs: Homo sapiens
+ Tg: taoniopygia guttat
+ Sc: Siniperca chuatsi

+ RAM: random access memory

R:
Thank you very much. We added these to the Abbreviation section.

- Page 17, in "Programming language", you should probably add "awk."

R:
We added AWK.

- Page 20 and after, please increase font for your commands.

R:
We have set the Font to Courier 10 now, line breaks are indicated now by "\" thus one could directly copy paste the commands into a linux terminal.

- Page 21, "comma separated list (e.g." -> "(e.g."

R:
We removed the whitespace letter.

- Page 23, please explain the "-p" and the "-l" parameters.

R:
We added the following two explanations:
"CSA uses the "-p" option to set the WTDBG2 consensus caller (0 = wtdbg-cons (default); 1 = wtpoa-cons; consensus calculation in step 1; 2 = wtdbg-cons -S 0; wtpoa-cns is slower but a bit more accurate; wtdbg-cns with option -S 0 is more stable on very long reads."

"CSA uses the –I "…" parameter to pass on detailed parameters to the wtdbg2 assembler. Parameters provided by –I "…" may overrule other wtdbg2 parameters which are set by CSA (e.g. -k, -s, -e or -m)."

Reviewer #2: Dear Colleagues,

I read with interest your manuscript on CSA, an assembly pipeline which uses synteny conservation across species. As you explain in the introduction, as massive vertebrate genome sequencing starts within the VGP project, not all genomes will get the same amount of attention, and such a pipeline would be useful in evening out the quality of assemblies.
I really liked the sequence of benchmarks, each with a clear setup. Overall the results look promising.

R:
Thank you very much!

A major concern I have however is the absence of comparison or reference to existing methods, as synteny-based scaffolding has been considered by other groups, e.g.:
https://www.jstage.jst.go.jp/article/gi1990/17/2/17_2_152/_pdf/-char/ja (reference 1)
https://bmcgenomics.biomedcentral.com/articles/10.1186/1471-2164-16-S10-S11 (ARt-DeCo)
https://genomebiology.biomedcentral.com/articles/10.1186/s13059-014-0573-1 (ALLMAPS)

R:
As reference 1 shows, the idea of using synteny in genome assembly is not new (we now do cite this reference among others proposed by reviewers#1 and #2). With our manuscript we do not suggest that we have invented synteny-based scaffolding, but want to make a strong example how useful this method is and especially will be in the future of large-scale animal genome projects. We also do not say CSA is the only way to assemble a chromosomal scale genome, but to our knowledge it is currently the computationally most efficient solution for long read data and provides a scheme of how future genome assembly tools could be structured. Regarding benchmarking issues, please, see also answers to reviewer #1.

ARt-DeCo: as the authors themselves state in their manuscript is lagging behind other methods as it does not use gene orientation.

ALLMAPS has been designed to integrate genetic linkage maps with draft genome assemblies. Although synteny data may be somehow fitted into ALLMAPS, other tools seem to be more tailored to do so (RACA or RAGOUT which is used in CSA).

Also, why do so many of the dot plots show local inversions? Is this a characteristic pattern of the assembler?

R:
We actually rather assume that as a characteristic of chromosome evolution. Many rearrangements between a diverged reference genome and the genome to be assembled may be inversions and sometimes (if the breakpoint region cannot be fully assembled) the true contig placement cannot be determined, in such a situation, the contig might be placed according to the reference genome and results in an inversion. This also explains that we obtain more inversions and translocation errors, if using more diverged reference genomes to assist CSA. A low contig N50 of the primary de novo assembly can have similar effects.

In response, we discuss this now in L384 - 387:

"It seems worth to mention that contig N50 length of the primary assembly (CSA step1) is an important factor and should be at least in the mega base range, as low contiguity of the contigs increases the chance of wrongly resolving rearrangements between query and reference genomes."

Close
