# Supplementary material for: CSA: A high-throughput chromosome-scale assembly pipeline for vertebrate genomes
Source: Gigascience. 2020 May 25;9(5):giaa034. doi: 10.1093/gigascience/giaa034 (PMC7247394; doi:10.1093/gigascience/giaa034)
Supplement: giaa034_Response_to_Reviewer_Comments_Revision_1 [file giaa034_response_to_reviewer_comments_revision_1.pdf]

Author's Response To Reviewer Comments

Close

Dear Dr. Edmunds,

we herewith submit our revised manuscript “CSA: A high-throughput chromosome-scale assembly pipeline for vertebrate genomes” for your consideration.
We tried to answer all open questions and changed the manuscript accordingly. In this regard, we would like to thank the reviewers and editor for their valuable comments.

We hope you may consider our manuscript to be published in Gigascience as a Technical Note.

Yours sincerely,

Dr. Heiner Kuhl
On behalf of all authors

Reviewer reports:
Reviewer #1: First, I would like to thank you for taking into consideration my suggestions.

Although I think that you have improved the quality of the article, I think that some questions have been addressed properly.

1. You did not discuss much the choice of wtdgb2. While I agree that it is a good tool, which is fast and memory efficient, you did not explain the reader why you chose it. You simply state (l. 141-2): "It emp loys the WGTDB2 (...) assembler as it among the most computationally efficient de novo genome assemblers to date." Again, I do agree, but if I were a naive reader, I would demand facts that support your choice.
Please cite papers that successfully used it, briefly present other methods (CANU, Flye, etc.), and state why you did not choose them. I agree that a full benchmark is out of the scope of this article (as previously said), but giving the reasons of your educated guess seems compulsory to me.

R:
We now comment on WTDBG2 speed improvements over competitors:

“It employs the WTDBG2 (version 2.2, 11 Dec. 2018) assembler as it is among the most computationally efficient de novo genome assemblers to date and according to [14] is 2-17 times as fast as its closest competitor.”

WTDBG2 claims to be the fastest assembly tool, by far outperforming CANU, FALCON and FLYE.
WTDBG2 may be challenged by SHASTA in the future. Currently, we tested SHASTA using its default parameters, but for us it worked only on Ultra long reads. WTDBG2 has just been published in Nature Methods and we have updated the reference.

2. I am still not convinced by the fact that you did not include a polisher. You stated in your response:
"A variety of methods are available. We think an in-depth analysis of consensus polishing methods would be a manuscript of its own and we would like to focus on chromosomal-level assembly in our manuscript". I never suggested a benchmark of the polishers. Just like the assembler, I am fine if you pick one tool, carefully explain why you chose it, and proceed with it.
Not using a polisher leads to half-true statements in your paper. For instance, in the Abstract > Background, you write: "Easy-to-use and fast bioinformatics pipelines for long-read assembly that go beyond the
contig-level to generate high-quality chromosome-scale genomes from raw data remain scarce". I understand what you mean by "high-quality chromosome-scale genomes": assembly, with possibly

many small scale error
s, but with a good genome contiguity. However, people usually think of genomes with few errors (including small errors) when they read "high-quality". Just Google "high-quality genome assembly", and you will mostly find articles with high Busco scores. Moreover, you used MEDAKA and PILON before providing the Busco score in Scenario 3. So I find that the description is somewhat misleading.

R:
We agree, by high-quality we actually mean high contiguity and completeness of chromosomal-sized scaffolds, and not necessarily high consensus quality. To make this clearer, we changed "high-quality" to "highly continuous" in L28 and L297. Nevertheless, high consensus quality is reached for CSA assemblies after polishing as we demonstrate in a single case, using Medaka and Pilon and the BUSCO analysis.

We agree and refer to optional polishing tools, now in L191-197.

"The current version of CSA does not include methods for consensus polishing, yet, a single iteration of consensus polishing using long-reads and two iterations using short-reads must be applied to obtain high quality consensus sequence and prior to sequence annotation efforts. We have made good experience using MEDAKA (by Oxford Nanopore Technologies: https://github.com/nanoporetech/medaka) and PILON in this regard (see benchmark scenario 3). Recently we have replaced MEDAKA by FLYE-POLISH [12] as it is able to use both SMRT (single molecule real time; Pacific Biosciences) and ONT (Oxford Nanopore Technologies) data."

We would like to emphasize furthermore that all of the widely used genome assembly tools like CANU, FALCON, FLYE and even SHASTA do not include polishing algorithms required for error rates below Q40 (i.e. for annotation without an excess of frameshift errors). In line with this common practice, we leave the choice of consensus polishing to the user. This is also, because choosing the right polishing pipeline for the different long-read technologies (SMRT or ONT) is a complex topic (e. g. involving different sequencing libraries and also depending on species properties) and in our opinion truly still requires some human decision making.

Last, I asked you in the first review why LAST and RAGOUT did not scaffold the contigs to yield a perfect assembly, when the original genome is given. You answered: "RAGOUT was designed to close gaps based on exact sequence matches between neighboring contig ends [...]. With long-read read assemblies, neighboring contig overlaps are typically noisy". If I understand well (correct me if I am wrong), polishing at least contig ends would correct them, and possibly help RAGOUT to scaffold them.

R:
Yes, polishing before RAGOUT would be possible, but may still not close all overlaps as it searches short overlaps (e.g. kmer-sized). We typically find overlaps of several thousand bp in long-read vertebrate assemblies between neighboring contigs in scaffolds. Another problem in diploid vertebrates are SNPs that could cause imperfect overlaps. Unfortunately, after any additional sequence improvement (e.g. gap closing) you would again need polishing the final output, and this would be computationally highly inefficient (as polishing nowadays requires more computational resources than assembly). In our experience polishing should always be the last step before annotation.

To sum up: Could you please give a reason why you did not include a polisher (the three lines you added in the text not explain your choice), or have them included in your pipe-line, since most of the assemblies do require some polishing.

R:
See above; now addressed in mscr.

Minor comments I did not see previously:

- Line 362-3, you state "but possibly most chromosome arms were well assembled". Please do not use weak sentences. How many chromosome arms well assembled? If so, what do you mean by "well assembled?" If you do not know, please remove the sentence.

R:
We removed "but possibly most chromosome arms were well assembled".

- Did you read the following assembly pipeline:
https://academic.oup.com/gigascience/article/8/4/giz014/5368071
The approach is different, but mentionning this paper could be interesting in the state-of-the-art section.

R:
Thanks, we added this as reference [15].

Details:
- Figure 1. After "Step N: ", the next character is sometimes in upper case, sometimes in lower case. Please be consistent.

R:
We corrected Figure 1.

- L. 364: You wrote "scenario2", without space between "scenario" and "2". However, line 376, you wrote "scenario 3", with a space. Please be consistent.

R:
We changed “scenario2” to “scenario 2”.

- Figures are sometimes called "Figure X", sometime "figure X". Same with tables, suppl. figures, and suppl. tables. Please be consistent here too.

R:
We use uppercase now for all “Figure” and “Table”.

Reviewer #2: Thank you for answering my questions.

R:
We thank both reviewers for their valuable comments!

Close
