# Supplementary material for: CSA: A high-throughput chromosome-scale assembly pipeline for vertebrate genomes
Source: Gigascience. 2020 May 25;9(5):giaa034. doi: 10.1093/gigascience/giaa034 (PMC7247394; doi:10.1093/gigascience/giaa034)
Supplement: giaa034_Reviewer_1_Report_Original_Submission — Matthias Zytnicki -- 12/9/2019 Reviewed [file giaa034_reviewer_1_report_original_submission.pdf]

Reviewer Report

Title: CSA: A high-throughput chromosome-scale assembly pipeline for vertebrate genomes

Version: Original Submission **Date:** 12/9/2019

Reviewer name: Matthias Zytnicki

Reviewer Comments to Author:

The paper presents a new pipeline for genome assembly. This pipeline uses reference genome, gap filling and assembly correction, for the production of the final assembly. The pipeline includes tools with a low memory footprint (relatively to genome assembly tools).
The paper is clearly written, and 6 use cases, which provide metrics on several scenarios, are presented.
This work should be useful for many lab around the world.
I have tried the example available on the GitHub repository. However, due to limited time to review this article (2 weeks), I did not have time to redo the analyses done in the article.
The package is easy to install and I could run seamlessly the small example.
However, I have major comments on the manuscript.
I feel that a description of the state of the art is missing.
When reading the paper, the user may wonder whether:
1. the pipeline deliver better results that other methods,
2. the selected features (gap filling, assembly correction, etc.) are the best mix (i.e. why did you choose these features over others?),
3. the selected tools (implementing the features) have been carefully selected.
Objective 1 has been partly addressed. However, I think that the other objectives deserve at least a discussion (an exhaustive benchmark may be out of scope).
Main questions:
- Concerning objective 1:
  + Please compare your approach/results with IMAP.
- Concerning objective 2:
  + Why did you not include other tools, such as polishers? You mentionned MEDAKA and PILON in scenario 3. If you did not include them in the pipeline, please mention why.
- Concerning objective 3:
  + Please discuss the use of RAGOUT (by the way, why not RAGOUT 2?), compared to other tools, such as MeDuSa or RACA.
  + Some tools have been developed for CSA: breaking contigs, joining contigs, closing gaps. Why did you implement these features, whereas solutions already exist: SMSC or BIGMAC for breaking contigs, LINKS for scaffolding, LR_Gapcloser or GAPPader for closing gaps? These tools have been published and benchmarked (to some extent). Do your tools perform better?
Other questions:
- Did you consider using Nextflow or Snakemake? This would probably help the users who work on a cluster for an easier interface with a job scheduler.

- I do not exactly understand why, on Scenario 5, the assembly is not perfect, even with low coverage, given that the very same genome is given as reference (as far as I understand it). Why is not LAST+RAGOUT stitching contigs?
- Scenario 6, do you compare ULR+CSA+GRCh38 to ULR+SHASTA? If so, since you provide the "true" genome, is not the first method highly favored?
- Could you please provide "meta-paramaters" to your tool? In minimap2, the author use the "-x" paramter. You could adopt something similar to handle Sequel reads.
Minor questions and comments:
- Page 3, there is probably something missing in the sentence: "[...] huge genome sizes of amphibians pose strong exceptions: c-value [...]"
- Page 3, what are "bird chromosomes"?
- Figure 1, the tool names written in the right hand of the rectangles are too small. Please enlarge.
- Figure 1, some rectangle texts start with a capital letter, some without. Why?
- Figure 2, please present the figures in the caption. The reader may not be familiar with minidot outputs. Please explain the meaning of:
  + the vertical and horizontal thin lines,
  + the color of the diagonal lines.
- Figure 2, please enlarge the dot plots, with at most two plots per row, preferably one.
- Page 10, the commas seem misplaced in "Overall the fraction of consensus sequences assigned to the top n scaffolds, was slightly lower than [...]"
- Scenario 3, please briefly present the data used in the "10X Genomics assembly," such as sequencing technology and coverage.
- Page 12, the comma seems superfluous in "To test, if CSA could reach [...]"
- Page 12, please explain "C: 95.9%, F: 2.1%, M: 2.0%, n: 4584"
- Page 13, I do not understand the comma in "Benchmark scenario 4: CSA using draft assemblies as reference, contig-level assemblies of diverged species may be highly complementary." It is a semi-colon?
- Other acronyms:
  + interchr.: inter-chromosomal
  + intrachr.: intra-chromosomal
  + f: inter-chromosomal fusion
  + t: intra-chromosomal translocation
  + i: inversion
  + Hs: Homo sapiens
  + Tg: taoniopygia guttat
  + Sc: Siniperca chuatsi
  + RAM: random access memory
- Page 17, in "Programming language", you should probably add "awk."
- Page 20 and after, please increase font for your commands.
- Page 21, "comma separated list (e.g." -> "(e.g."
- Page 23, please explain the "-p" and the "-l" parameters.

Level of Interest

Please indicate how interesting you found the manuscript: Choose an item.

Quality of Written English

Please indicate the quality of language in the manuscript: Choose an item.

Declaration of Competing Interests

Please complete a declaration of competing interests, considering the following questions:

- Have you in the past five years received reimbursements, fees, funding, or salary from an organisation that may in any way gain or lose financially from the publication of this manuscript, either now or in the future?
- Do you hold any stocks or shares in an organisation that may in any way gain or lose financially from the publication of this manuscript, either now or in the future?
- Do you hold or are you currently applying for any patents relating to the content of the manuscript?
- Have you received reimbursements, fees, funding, or salary from an organization that holds or has applied for patents relating to the content of the manuscript?
- Do you have any other financial competing interests?
- Do you have any non-financial competing interests in relation to this paper?

If you can answer no to all of the above, write 'I declare that I have no competing interests' below. If your reply is yes to any, please give details below.

I declare that I have no competing interests.

I agree to the open peer review policy of the journal. I understand that my name will be included on my report to the authors and, if the manuscript is accepted for publication, my named report including any attachments I upload will be posted on the website along with the authors' responses. I agree for my report to be made available under an Open Access Creative Commons CC-BY license (http://creativecommons.org/licenses/by/4.0/). I understand that any comments which I do not wish to be included in my named report can be included as confidential comments to the editors, which will not be published.

Choose an item.

To further support our reviewers, we have joined with Publons, where you can gain additional credit to further highlight your hard work (see: https://publons.com/journal/530/gigascience). On publication of

this paper, your review will be automatically added to Publons, you can then choose whether or not to claim your Publons credit. I understand this statement.

Yes Choose an item.
