# Supplementary material for: CSA: A high-throughput chromosome-scale assembly pipeline for vertebrate genomes
Source: Gigascience. 2020 May 25;9(5):giaa034. doi: 10.1093/gigascience/giaa034 (PMC7247394; doi:10.1093/gigascience/giaa034)
Supplement: giaa034_Reviewer_1_Report_Revision_1 — Matthias Zytnicki -- 2/18/2020 Reviewed [file giaa034_reviewer_1_report_revision_1.pdf]

Reviewer Report

Title: CSA: A high-throughput chromosome-scale assembly pipeline for vertebrate genomes

Version: Revision 1 **Date:** 2/18/2020

Reviewer name: Matthias Zytnicki

Reviewer Comments to Author:

First, I would like to thank you for taking into consideration my suggestions.
Although I think that you have improved the quality of the article, I think that some questions have been addressed properly.
1. You did not discuss much the choice of wtdgb2. While I agree that it is a good tool, which is fast and memory efficient, you did not explain the reader why you chose it. You simply state (l. 141-2): "It emp loys the WGTDB2 (...) assembler as it among the most computationally efficient de novo genome assemblers to date." Again, I do agree, but if I were a naive reader, I would demand facts that support your choice.
Please cite papers that successfully used it, briefly present other methods (CANU, Flye, etc.), and state why you did not choose them. I agree that a full benchmark is out of the scope of this article (as previously said), but giving the reasons of your educated guess seems compulsory to me.
2. I am still not convinced by the fact that you did not include a polisher. You stated in your response: "A variety of methods are available. We think an in-depth analysis of consensus polishing methods would be a manuscript of its own and we would like to focus on chromosomal-level assembly in our manuscript". I never suggested a benchmark of the polishers. Just like the assembler, I am fine if you pick one tool, carefully explain why you chose it, and proceed with it.
Not using a polisher leads to half-true statements in your paper. For instance, in the Abstract > Background, you write: "Easy-to-use and fast bioinformatics pipelines for long-read assembly that go beyond the
contig-level to generate high-quality chromosome-scale genomes from raw data remain scarce". I understand what you mean by "high-quality chromosome-scale genomes": assembly, with possibly many small scale error
s, but with a good genome contiguity. However, people usually think of genomes with few errors (including small errors) when they read "high-quality". Just Google "high-quality genome assembly", and you will mostly find articles with high Busco scores. Moreover, you used MEDAKA and PILON before providing the Busco score in Scenario 3. So I find that the description is somewhat misleading.
Last, I asked you in the first review why LAST and RAGOUT did not scaffold the contigs to yield a perfect assembly, when the original genome is given. You answered: "RAGOUT was designed to close gaps based on e
xact sequence matches between neighboring contig ends [...]. With long-read read assemblies, neighboring contig overlaps are typically noisy". If I understand well (correct me if I am wrong), polishing at leas
t contig ends would correct them, and possibly help RAGOUT to scaffold them.

To sum up: Could you please give a reason why you did not include a polisher (the three lines you added in the text not explain your choice), or have them included in your pipe-line, since most of the assemblies do require some polishing.
Minor comments I did not see previously:
- Line 362-3, you state "but possibly most chromosome arms were well assembled". Please do not use weak sentences. How many chromosome arms well assembled? If so, what do you mean by "well assembled?" If yo
u do not know, please remove the sentence.
- Did you read the following assembly pipe-line:
https://academic.oup.com/gigascience/article/8/4/giz014/5368071
The approach is different, but mentionning this paper could be interesting in the state-of-the-art section.
Details:
- Figure 1. After "Step N: ", the next character is sometimes in upper case, sometimes in lower case. Please be consistent.
- L. 364: You wrote "scenario2", without space between "scenario" and "2". However, line 376, you wrote "scenario 3", with a space. Please be consistent.
- Figures are sometimes called "Figure X", sometime "figure X". Same with tables, suppl. figures, and suppl. tables. Please be consistent here too.

Level of Interest

Please indicate how interesting you found the manuscript: Choose an item.

Quality of Written English

Please indicate the quality of language in the manuscript: Choose an item.

Declaration of Competing Interests

Please complete a declaration of competing interests, considering the following questions:

- Have you in the past five years received reimbursements, fees, funding, or salary from an organisation that may in any way gain or lose financially from the publication of this manuscript, either now or in the future?
- Do you hold any stocks or shares in an organisation that may in any way gain or lose financially from the publication of this manuscript, either now or in the future?
- Do you hold or are you currently applying for any patents relating to the content of the manuscript?
- Have you received reimbursements, fees, funding, or salary from an organization that holds or has applied for patents relating to the content of the manuscript?
- Do you have any other financial competing interests?

- Do you have any non-financial competing interests in relation to this paper?

If you can answer no to all of the above, write 'I declare that I have no competing interests' below. If your reply is yes to any, please give details below.

I declare that I have no competing interests.

I agree to the open peer review policy of the journal. I understand that my name will be included on my report to the authors and, if the manuscript is accepted for publication, my named report including any attachments I upload will be posted on the website along with the authors' responses. I agree for my report to be made available under an Open Access Creative Commons CC-BY license (http://creativecommons.org/licenses/by/4.0/). I understand that any comments which I do not wish to be included in my named report can be included as confidential comments to the editors, which will not be published.

Choose an item.

To further support our reviewers, we have joined with Publons, where you can gain additional credit to further highlight your hard work (see: https://publons.com/journal/530/gigascience). On publication of this paper, your review will be automatically added to Publons, you can then choose whether or not to claim your Publons credit. I understand this statement.

Yes Choose an item.
